# Clustering of host N-glycans by the microneme MIC1/4/6 complex licenses *Toxoplasma* rhoptry discharge

**DOI:** 10.1038/s44318-026-00911-z

**Published:** 2026-09-25

**Authors:** Dylan Valleau, Mudita Goyal, Justin M Roberts, Frances Male, Gary E Ward, Sebastian Lourido

**Affiliations:** 1 Whitehead Institute, Cambridge, MA USA; 2 Biology Department, Massachusetts Institute of Technology, Cambridge, MA USA; 3 Department of Microbiology and Molecular Genetics, University of Vermont, Larner College of Medicine, Burlington, VT USA

**Keywords:** Membranes & Trafficking, Microbiology, Virology & Host Pathogen Interaction

## Abstract

To initiate the process of invasion, apicomplexan parasites must discharge the contents of specialized organelles called rhoptries into host cells. This process requires prior recognition and binding of the host cell by proteins released from another set of parasite organelles, the micronemes. However, the host–parasite interactions required for rhoptry discharge are largely unknown. Here, we performed a host cell-directed genome-wide screen for factors that mediate rhoptry discharge from *Toxoplasma gondii*, the causative agent of toxoplasmosis. The screen identified host N-glycosylation and cholesterol biosynthesis as pathways required for normal rhoptry discharge. A trimeric microneme complex, MIC1/4/6, interfaces with both pathways by binding host N-glycans to cluster proteins in a process dependent on host plasma membrane cholesterol. The process can be inhibited by depletion of host cholesterol or competition with exogenous glycans. This clustering of host factors by MIC1/4/6 likely prepares the host membrane for rhoptry discharge, delineating a new step in the *Toxoplasma* invasion process.

## Synopsis

Host cell invasion by the apicomplexan parasite *Toxoplasma gondii* involves sequential protein discharge from specialized organelles, micronemes and rhoptries. This study reveals that the clustering of the host N-glycans into cholesterol-dependent microdomains by the microneme MIC1/4/6 complex promotes rhoptry discharge to mediate invasion.

- A host-directed genome-wide CRISPR screen reveals enrichment of mutants in N-glycosylation or cholesterol biosynthesis among cells refractory to *T.* *gondii* rhoptry discharge.
- Galactose-containing host N-glycans are specifically required for rhoptry discharge.
- The microneme MIC1/4/6 complex on the parasite membrane binds host N-glycans through dual recognition of capping sialic acid and galactose.
- MIC1/4/6 complex function is dependent on host cholesterol and operates independently of the known rhoptry discharge-promoting CLAMP and CRMP complexes.

## Abstract (web summary)

Initiation of cell invasion by *Toxoplasma gondii* requires clustering of the host N-glycans into cholesterol microdomains by the parasite’s MIC1/4/6 complex.

## Graphical abstract

**Figure:**
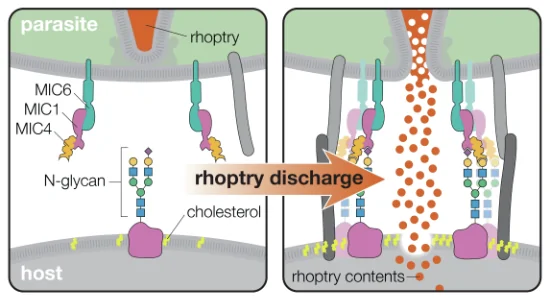

## Introduction

Invasion of host cells is required to establish the replicative niche of apicomplexan parasites. Studies of zoites from multiple stages of *Toxoplasma gondii*, *Plasmodium* spp., and *Cryptosporidium* spp. have identified common steps in invasion, including initial attachment, reorientation, penetration (or enveloping in the case of *Cryptosporidium*), and fission of the parasitophorous vacuole from the host plasma membrane (Weiss et al, 2015; Wetzel et al, 2005; Carruthers and Boothroyd, 2007). In *T. gondii*, the entire process is completed in less than a minute (Bichet et al, 2016). Progression through the sequence of events is highly regulated and depends on the discharge of micronemes and rhoptries, specialized organelles that secrete proteins from the apical end of parasites (Carruthers and Sibley, 1997; Bisio and Soldati-Favre, 2019). Micronemes are numerous, and their contents are released in waves to support egress, gliding motility, and invasion (Carruthers and Sibley, 1999). By contrast, zoites have limited numbers of the larger club-shaped rhoptries, ranging from a single one in *Cryptosporidium* spp. sporozoites to up to a dozen in *Toxoplasma* tachyzoites. The timing of rhoptry discharge is precisely regulated to deliver the arsenal of effector proteins that then drive invasion and host remodeling directly into host cells (Carruthers and Sibley, 1997; Cova et al, 2022; Cowman et al, 2017). Several groups have shown that prior secretion of certain microneme proteins is necessary, but not sufficient, for rhoptry discharge (Valleau et al, 2023; Singer et al, 2023; Sparvoli et al, 2022; Kessler et al, 2008).

Microneme protein secretion initiates during egress and continues throughout the extracellular phase of the parasite life cycle, up to the moment of invasion. Micronemes fuse with the apical plasma membrane in response to waves of Ca^2+^ release from both intracellular stores and the extracellular environment (Pace et al, 2014; Carruthers and Sibley, 1999). The signaling pathways that regulate microneme exocytosis include cGMP production and PKG upstream of the Ca^2+^ surge (Brown et al, 2016), and calcium-dependent protein kinases (CDPKs), notably CDPK1, in the transduction of the signals (Carruthers and Sibley, 1997; Bisio and Soldati-Favre, 2019). DOC2 and ferlins have also been implicated in the apical exocytosis of micronemes (Tagoe et al, 2021; Farrell et al, 2012; Tagoe et al, 2024), which releases luminal contents into the extracellular space and incorporates transmembrane proteins and associated complexes onto the parasite plasma membrane. Microneme proteins then perform several functions critical for invasion, including mediating adhesion to the host during gliding motility and licensing rhoptry discharge upon host cell contact (Frénal et al, 2017; Cova et al, 2022).

Several processes contribute to the overall efficiency of rhoptry discharge. Gliding motility and attachment are not strictly required but enhance rates of rhoptry discharge by promoting proper apposition of the parasite apical end and the host cell membrane. Indeed, the effects of gliding motility can be excluded from rhoptry discharge assays by blocking actin polymerization (e.g., Cytochalasin D or Mycalolide B) or disrupting key motors like MyoH (Graindorge et al, 2016). Analogously, the formation of the moving junction increases parasite attachment, which can be interpreted as increased rhoptry discharge when visually quantifying the release of rhoptry contents in proximity to attached parasites (i.e., evacuole formation) (Lamarque et al, 2014); however, impairment of the moving junction or adhesion does not affect rhoptry discharge when the process is evaluated directly (Male et al, 2025; Valleau et al, 2023; Lamarque et al, 2014). For example, the microneme-secreted adhesins MIC2 and AMA1 participate in parasite adhesion and moving junction formation, respectively (Huynh and Carruthers, 2006; Tyler and Boothroyd, 2011; Lamarque et al, 2011), but their disruption does not affect rhoptry discharge when the process is measured while blocking gliding motility (Valleau et al, 2023; Singer et al, 2023). It should be noted that current methods for measuring rhoptry discharge do not formally distinguish between the release of rhoptry proteins from the parasite and their translocation into host cells, so discharge will be used as a unified term for the ensemble. Contrasting with the adhesion pathways, several microneme proteins have been specifically linked to rhoptry discharge in *T. gondii* without generally affecting secretion of other microneme proteins, disrupting gliding motility, or interfering with organelle biogenesis. Such factors include MIC7, MIC8, and components of the CLAMP and CRMP complexes (Valleau et al, 2023; Singer et al, 2023; Sparvoli et al, 2022; Broncel et al, 2020). The CLAMP and CRMP complexes are the most conserved of these factors, as their core proteins are generally encoded by all apicomplexan genomes; however, their precise function in rhoptry discharge remains unknown.

In contrast to microneme exocytosis via plasma membrane fusion, rhoptry discharge occurs via an elaborate rhoptry secretion apparatus (RSA). Ultrastructural studies have shown that the RSA is constitutively embedded in the plasma membrane at the apical tip of the parasite, visualized as a rosette in *T. gondii* (Mageswaran et al, 2021; Aquilini et al, 2021). The rhoptries are docked to this apparatus via an apical vesicle (AV), so content release is thought to occur through two regulated steps: fusion of the rhoptry with the AV and AV fusion with the parasite plasma membrane at the RSA site (Martinez et al, 2022; Mageswaran et al, 2021; Segev-Zarko et al, 2022; Cova et al, 2022). Recent high-speed imaging and electrophysiology have uncovered that rhoptry discharge by *T. gondii* is associated with the transient perforation of the host cell plasma membrane (Male et al, 2025; Kegawa et al, 2025). The erythrocytic blood stages of *Plasmodium falciparum* also form a pore in the erythrocyte plasma membrane prior to invasion, suggesting that permeabilization is a common feature of apicomplexan invasion, though the identity of the perforating factors remains unclear (Weiss et al, 2015; Geoghegan et al, 2021).

Disruption of the host membrane provides a plausible mechanism for the translocation of rhoptry proteins directly into the host cytoplasm, following discharge through the RSA. Translocation of *T. gondii* rhoptry effectors can be used to measure rhoptry discharge directly or indirectly. Discharge of the rhoptry protein ROP16 leads to the phosphorylation and nuclear accumulation of the host transcription factor STAT6, which can be readily quantified by immunofluorescence (Ong et al, 2010). Alternatively, Cre recombinase or a β-lactamase fused to the rhoptry protein Toxofilin can be used to indirectly detect rhoptry discharge by measuring the activity of fused enzymes (Koshy et al, 2010, 2012; Lodoen et al, 2010). Notably, measurement of β-lactamase activity in host cells remains the most rapid and scalable method to quantify rhoptry discharge. After translocation, some rhoptry proteins, including the rhoptry neck (RON) complex, are embedded into the host plasma membrane. The C-terminal tail of RON2 acts as an anchor for the microneme protein AMA1, which in turn is tethered to the parasite plasma membrane and the parasite actomyosin system for force generation (Lamarque et al, 2011; Frénal et al, 2017; Lamarque et al, 2014; Haile et al, 2026). Together, this RON2–AMA1 interaction forms the core of the moving junction through which the parasite invades.

Several host factors contribute to invasion. *T. gondii* adhesion can be disrupted by blocking interactions between sulfated proteoglycans like heparin and MIC2 (Huynh and Carruthers, 2006; Harper et al, 2004; Gras et al, 2017; Carruthers et al, 2000). Host cholesterol also appears to be required for efficient invasion and is found at the site of invasion, but the interacting parasite factors remain unknown (Coppens and Joiner, 2003; Tahara et al, 2016; Besteiro et al, 2008). In *P. falciparum*, the clade-specific *Pf*RCR complex mediates binding to host basigin and α2,6-linked Neu5Ac (sialic acid) on red blood cells, and is thought to be involved in rhoptry discharge (Farrell et al, 2024; Day et al, 2024; Cowman et al, 2017). Many of the *T. gondii* rhoptry discharge factors noted above have adhesive domains that similarly suggest affinity for specific host cell ligands, although no specific host receptors have been identified.

We set out to identify the host factors specifically required for *T. gondii* rhoptry discharge, employing a host-directed genome-wide CRISPR/Cas9-based screen. The analysis revealed that host cholesterol and N-glycosylation are critical for rhoptry discharge. We further identify the microneme complex that intersects with these two host pathways to license rhoptry discharge and promote infection, establishing the MIC1/4/6 complex as a novel regulator of rhoptry discharge.

## Results

### A genome-wide knockout CRISPR screen reveals host determinants of rhoptry discharge

**Figure 1:**
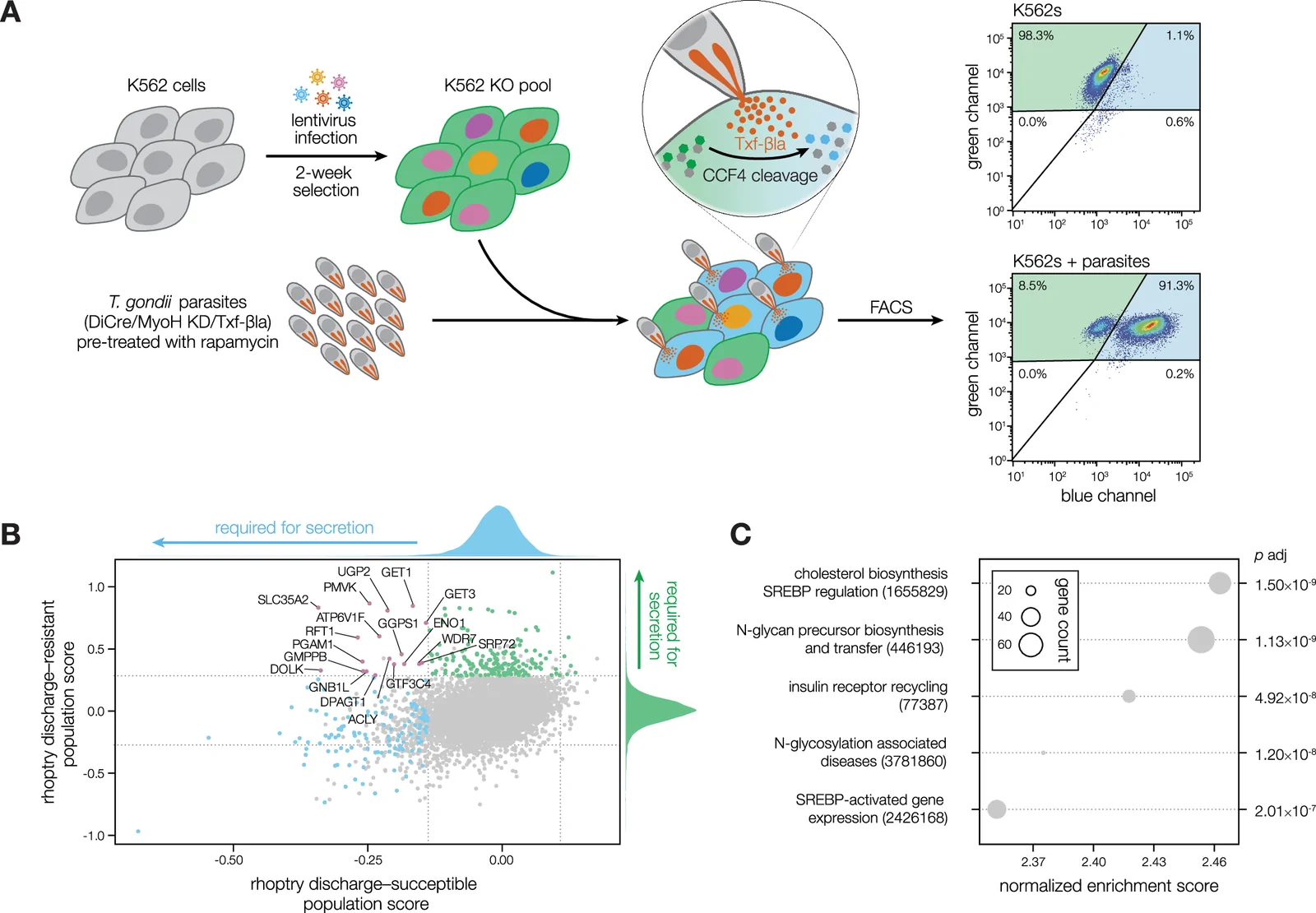
A host-directed screen reveals genes required for rhoptry discharge. (**A**) A genome-wide knockout pool was generated in the human leukemia K562 cell line using CRISPR-based gene disruption. The host cell pool was challenged with rapamycin-treated DiCre/MyoH cKD/Txf-βla *T. gondii* parasites, then loaded with CCF4-AM and sorted into populations refractory (green) or susceptible (blue) to rhoptry discharge. Sample plots show the relative signal of CCF4 with or without parasite-delivered Txf-βla. (**B**) MAGeCK MLE analysis of guide RNA abundance in sorted host cell populations. Beta-scores calculated by the MLE pipeline are plotted. The distribution of scores for each population is displayed along each axis. Genes are colored based on enrichment of targeting guide RNAs in the population refractory to rhoptry discharge (green), depletion from the susceptible population (blue), or presence in both groups (pink). Inclusion in each group was based on s.d. >3 and Wald test *P* value < 0.05. Dashed lines represent the s.d. equivalent to 3 for each distribution. (**C**) GSEA of the top human Reactome pathways required for rhoptry discharge into host cells.

To identify host determinants of *T. gondii* rhoptry discharge, we developed a screening approach using a previously established FRET-based assay (Lodoen et al, 2010). In this assay, rhoptry discharge is measured using parasites expressing the secreted rhoptry effector Toxofilin conjugated to β-lactamase (Txf-βla). After reporter discharge, host cells can be loaded with the coumarin-fluorescein FRET substrate CCF4-AM. CCF4 fluoresces green (520 nm) when excited at a 405 nm wavelength. After Txf-βla cleaves the β-lactam ring in CCF4, FRET uncoupling results in a shift to blue fluorescence (447 nm; Fig. 1A). We introduced Txf-βla into a *T. gondii* strain engineered for conditional knockdown of MyoH, a parasite myosin required for motility and invasion but dispensable for microneme and rhoptry discharge (Graindorge et al, 2016; Dos Santos Pacheco et al, 2022). Conditional knockdown of MyoH was achieved using a rapamycin-dimerizable Cre recombinase (DiCre), which causes post-transcriptional regulation of MyoH levels using the U1 system (DiCre/MyoH cKD/Txf-βla) (Hunt et al, 2019; Pieperhoff et al, 2015). This parasite background limits confounding effects from other steps of parasite invasion, like motility and actomyosin-driven invasion, and focuses the screen on factors required for rhoptry discharge. Other factors upstream of rhoptry discharge, such as those required for parasite attachment, may also be identified along with those directly involved in mediating rhoptry discharge.

To perform the screen, human erythroleukemia K562 cells were transduced with a pooled 5-guide lentivirus CRISPR-based library targeting most protein-coding genes for disruption (97,888 guides targeting 19,665 genes) (Inglis et al, 2023). On days 13 and 14 post transduction, the pools of K562 knockout host cells were allowed to interact with MyoH knockdown parasites. Parasites were added at a multiplicity of infection of 20, which achieved 90% CCF4 conversion in the K562 pool (Fig. 1A). After 1 h of interaction, CCF4-AM was added, and K562 cells were sorted into two populations: green (refractory to rhoptry discharge) and blue (susceptible to rhoptry discharge). We performed the screen in two independently transduced K562 pools, each challenged with parasites on two separate days, for a total of four experimental replicates. Integrated guide RNAs were amplified from each population, and their relative abundances in each sample were measured by next-generation sequencing.

**Figure EV1:**
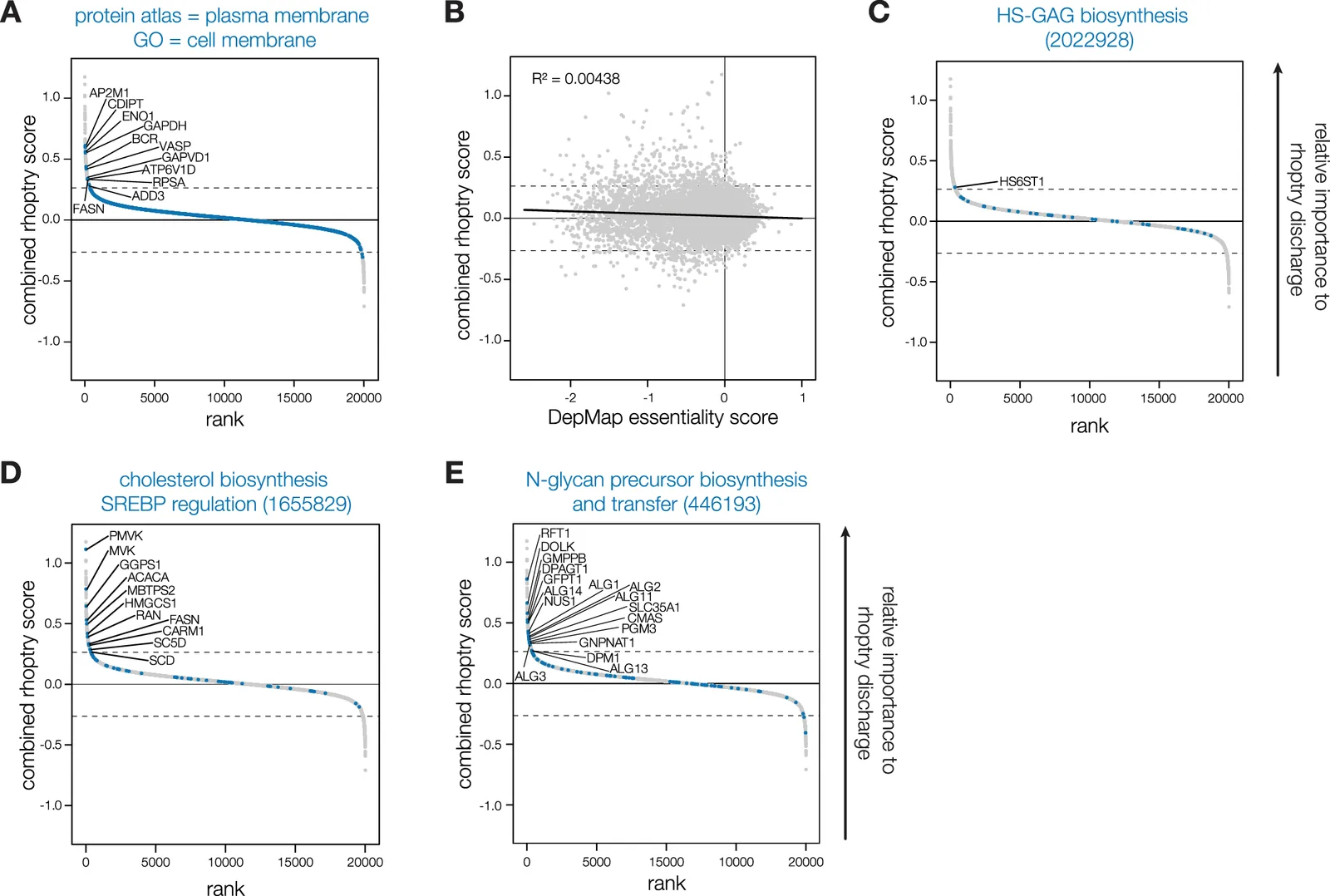
Involvement of relevant gene categories in rhoptry discharge. (**A**) Rank-ordered position of genes that are displayed in the plasma membrane or surface exposed as annotated by GO terms and the Human Protein Atlas in the rhoptry discharge screen. Dashed line indicates MLE estimation of s.d. = 3. All genes above this threshold are labeled. (**B**) Comparison of the DepMap K562 gene essentiality score to the combined rhoptry score. Regression analysis reveals nearly zero correlation between essentiality in cell culture and gene score in the rhoptry discharge screen. Dashed lines indicate MLE estimation of s.d. =  3. (**C**) Rank-ordered position of genes in the heparin sulfate GAG biosynthesis pathway in the rhoptry discharge screen. Genes displayed are from the Reactome pathway R-HSA-2022928. The dashed line indicates MLE estimation of s.d. = 3. All genes above this threshold are labeled. (**D**) Rank-ordered position of genes required for regulation of cholesterol biosynthesis in the rhoptry discharge screen. Genes displayed are from the Reactome pathway R-HSA-1655829. The dashed line indicates MLE estimation of s.d. = 3. All genes above this threshold are labeled. (**E**) Rank-ordered position of genes required for N-glycan dolichol precursor biosynthesis and transfer to a nascent protein in the rhoptry discharge screen. Genes displayed are from the Reactome pathway R-HSA-446193. The dashed line indicates MLE estimation of s.d. = 3. All genes above this threshold are labeled.

Using the maximum-likelihood estimation (MLE) MAGeCK pipeline, we compared sorted populations to their respective unsorted controls (Fig. 1B; Dataset EV1). Two hundred factors were deemed critical for rhoptry discharge based on either their significant enrichment in the discharge-resistant (green) population or their significant depletion from the discharge-sensitive (blue) populations, with 125 factors overlapping between the two categories—18 of which were both at least three standard deviations different from the mean and achieved significance in at least one population. Proteins localized to the plasma membrane were largely absent among putative rhoptry discharge factors (Fig. EV1A) (Ashburner et al, 2000; Gene Ontology Consortium et al, 2023; Thul et al, 2017), which may result from redundant extracellular proteins mediating rhoptry discharge. The minimal correlation between K562 gene essentiality scores and the rhoptry screen scores suggests our screen was not broadly biased by essential gene depletion (Fig. EV1B). Nevertheless, some mutants in essential genes may be too depleted to be captured in the screen. It is also possible that *T. gondii* does not depend on any single receptor for infection, perhaps explaining its broad tropism. Despite the absence of specific membrane proteins, several top-scoring genes included factors involved in membrane protein biogenesis or trafficking, including many components of the guided entry of tail-anchored proteins complex (GET), endoplasmic reticulum membrane protein complex (EMC), and signal recognition particle complex (SRP; Fig. 1B), revealing that the display of surface proteins is generally important for rhoptry discharge.

To categorize host genes important for rhoptry discharge, we combined the scores from refractory and susceptible populations and performed gene set enrichment analysis (GSEA) against the Reactome database (Milacic et al, 2024). Pathways previously found to be involved in parasite attachment—notably, the biosynthesis of heparin sulfate, which is bound by the adhesion-related microneme protein MIC2—are not strongly enriched for putative discharge factors (Fig. EV1C), consistent with our screen specifically targeting the step of rhoptry discharge (Carruthers et al, 2000; Harper et al, 2004). By contrast, pathways related to cholesterol and N-glycan biosynthesis appeared as required for rhoptry discharge (Figs. 1C and EV1D,E). Cholesterol-related genes required for rhoptry discharge included synthesis genes like MSMO1 (Sukhanova et al, 2013); mevalonate pathway genes (e.g., MVK and PMVK), which supply prenyl precursors for cholesterol biosynthesis, geranylgeranylation, and farnesylation (Fig. EV1D); and the transcriptional regulators of sterol production, SREBP/SERBF, along with some of their downstream genes (e.g., FASN, HMGCR) (Fidelito et al, 2025). In the N-glycan pathway, we detected a striking number of genes responsible for synthesizing the essential dolichol glycan precursors and shuttling them to the ER lumen, including DOLK, DPAGT1, RFT1, and DDOST, as well as genes required for synthesizing and trimming the core N-glycan structure, such as many ALG genes (Fig. EV1E) (Schjoldager et al, 2020). The requirement for synthesis of N-linked glycoproteins, which are predominantly displayed on the host cell surface, is consistent with the involvement of the aforementioned EMC and SRP complexes in rhoptry discharge. Interestingly, some of the rhoptry discharge determinants, such as NUS1, are important for the synthesis of the dolichol N-glycan precursor from mevalonate-pathway products, suggesting a possible integration of these two categories of rhoptry discharge factors (Mullen et al, 2016; Fidelito et al, 2025).

### Inhibition of host N-glycosylation and cholesterol biosynthesis impairs rhoptry discharge

**Figure 2:**
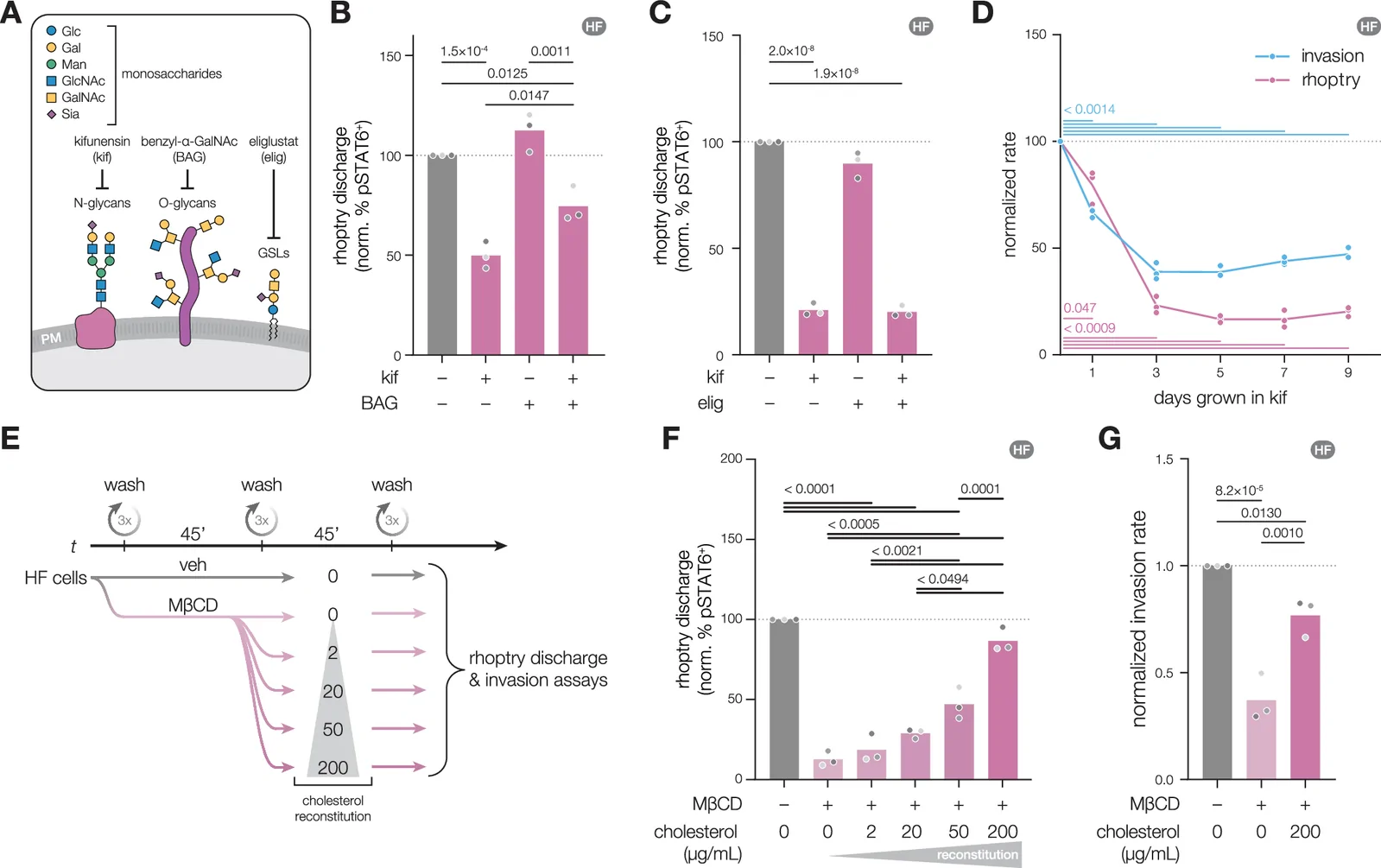
Host N-glycosylation and cholesterol are required for rhoptry discharge. (**A**) Schematic of major types of glycosylated molecules found on the mammalian plasma membrane, along with inhibitors that block their synthesis. (**B**, **C**) Human fibroblasts were treated with the N-glycosylation inhibitor kif (10 μM) alone or in combination with the O-glycosylation inhibitor BAG (2 mM) for 3 days (**B**), or the glycosphingolipid inhibitor elig (2 μM) for 5 days (**C**). Host cells were then challenged with wild-type parasites, and rhoptry discharge was assessed by the relative percent of pSTAT6-positive nuclei. Mean of *n* = 3 biological replicates is plotted; *P* values derived from one-way ANOVA with Tukey’s post hoc test. (**D**) Time-course of kif (10 μM) treatment on rhoptry discharge and parasite invasion. Dividing human fibroblasts were pre-treated for the indicated numbers of days, then all timepoints were challenged with dTomato-expressing wild-type parasites. Rhoptry discharge was assessed by the relative percent of pSTAT6–positive nuclei and invasion by counting the number of internalized parasites per host cell nucleus. Mean of *n* = 3 biological replicates is plotted. For invasion and rhoptry discharge experiments, *P* values are derived from one-sample *t* tests compared to the day 0 normalized value representing 100%. Exact *P* values are provided in the Source Data. (**E**) To deplete cholesterol, human fibroblasts were treated for 45 min with MβCD. Varying concentrations of water-soluble cholesterol were then used to reconstitute cholesterol into the fibroblasts for 45 min prior to parasite challenge. (**F**) Rhoptry discharge into human fibroblasts with varying levels of cholesterol. Host cells were challenged with wild-type parasites, and rhoptry discharge was measured by the relative percent of pSTAT6-positive nuclei. Mean of *n* = 3 biological replicates is plotted; *P* values derived from one-way ANOVA with Tukey’s post hoc test. Exact *P* values are provided in the Source Data. (**G**) Parasite invasion rates into human fibroblasts with depleted and reconstituted (200 µg/mL) levels of membrane cholesterol. Invasion rates were assessed by counting the number of internalized parasites per host cell nucleus. Mean of *n* = 3 biological replicates is plotted. *P* values derived from one-way ANOVA with Fisher’s LSD post-hoc test. Source data are available online for this figure.

N-glycosylation was the dominant glycosylation pathway required for rhoptry discharge identified by our screen. Enzymes specific to O-glycosylation and glycosphingolipid synthesis were largely absent among the top required factors, despite the presence of key genes that are shared with N-glycosylation, such as precursor biosynthesis and Golgi transport (Schjoldager et al, 2020). To confirm that N-glycans are the primary glycans involved in rhoptry discharge, we used pathway-selective inhibitors. We pre-treated human fibroblasts (HF) with kifunensine (kif), an inhibitor of alpha-mannosidases required for the formation of complex and hybrid N-glycans (Elbein et al, 1990; Chang et al, 2007), with benzyl-α-GalNAc (BAG), a competitive inhibitor of mucin-type O-glycan initiation (Wang et al, 2021; Kuan et al, 1989), or with eliglustat (elig), a competitive inhibitor of UGCG, the rate-limiting enzyme in glycosphingolipid biosynthesis (Dong et al, 2024; Stirnemann et al, 2017) (Fig. 2A). Rhoptry discharge was assessed by monitoring the percentage of host cells displaying phosphorylation and nuclear translocation of the transcription factor STAT6, which is induced by the secreted rhoptry effector ROP16 (Ong et al, 2010). To focus assays specifically on rhoptry discharge, parasites were treated with mycalolide B, an irreversible inhibitor of actin polymerization that blocks motility and invasion—analogous to our use of MyoH knockdown in the screens (Valleau et al, 2023).

**Figure EV2:**
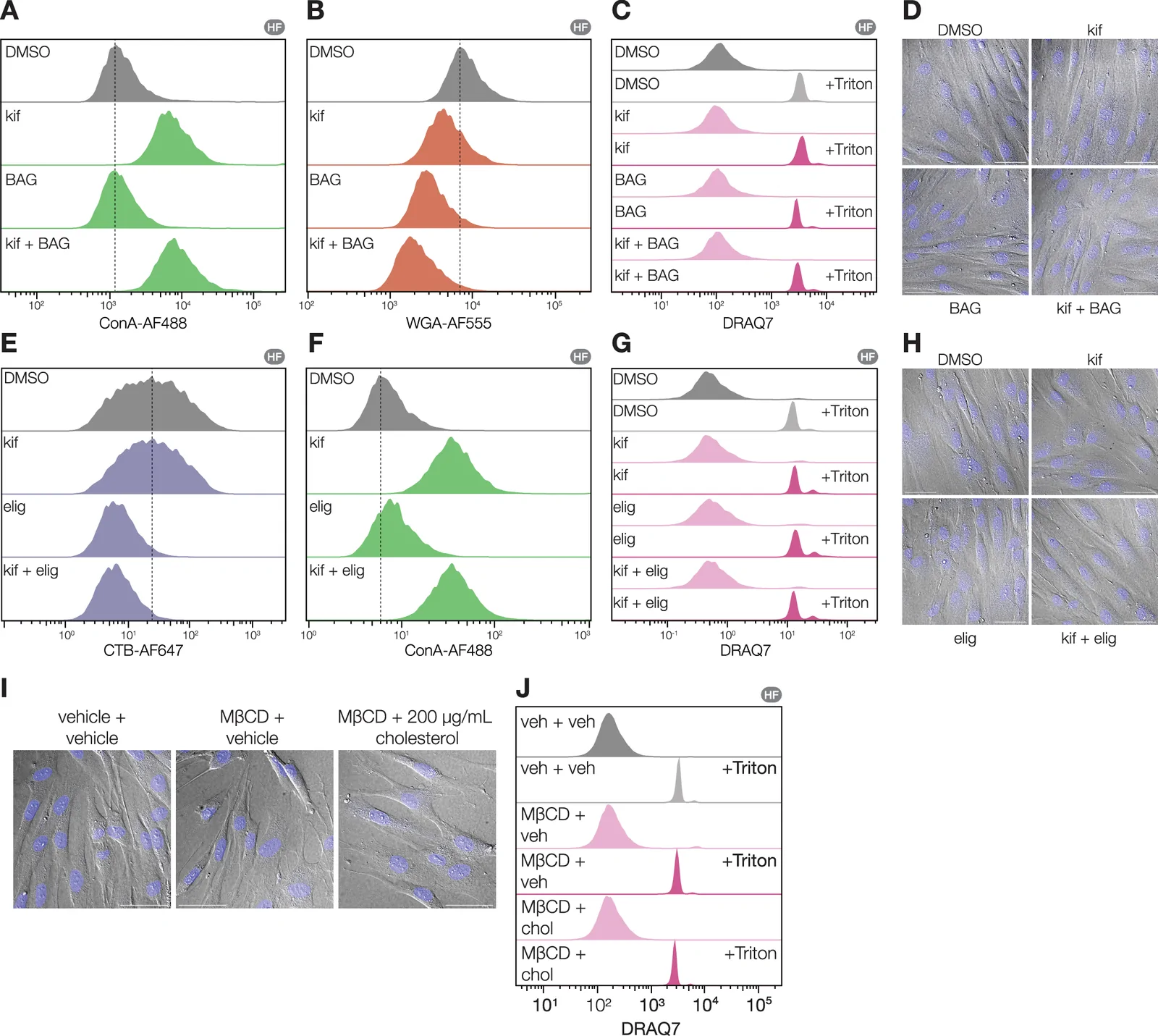
Host cell drug treatments and knockouts result in expected surface glycosylation patterns. (**A**, **B**) Flow cytometry analysis of human fibroblast surface glycosylation following a 3-day treatment with vehicle (DMSO), kif (10 μM), BAG (2 mM), or a combination of kif and BAG. Exposed mannose was stained using the lectin ConA conjugated to Alexa Fluor 488 (ConA-AF488; **A**), and exposed GlcNAc and Neu5Ac were stained using WGA conjugated to Alexa Fluor 555 (WGA-555; **B**). (**C**) Cell viability and membrane integrity of human fibroblasts following a 3-day combination of kif and BAG treatments, as evaluated by exclusion of the cell-impermeable far-red DNA dye DRAQ7. Triton X-100 (Triton; 0.02%) was added as a positive control for permeabilization. (**D**) Analysis of normal human fibroblast morphology following 3-day combinations of kif and BAG treatments, assessed by live DIC microscopy. Cell-permeable Hoechst 33342 was included to visualize nuclei (blue). A slice of a Z-stack is shown based on nuclear stain focus. White scale bars indicate 50 μm. (**E**, **F**) Flow cytometry analysis of human fibroblast surface glycosylation following a 5-day treatment with vehicle (DMSO), kif (10 μM), elig (2 μM), or a combination of kif and elig. The plasma membrane glycosphingolipid GM1 was visualized using Alexa Fluor 647-conjugated Cholera toxin B domain (CTBB-AF647; **E**), and exposed mannose was stained using the lectin ConA conjugated to Alexa Fluor 488 (ConA-AF488; **F**). (**G**) Cell viability and membrane integrity of human fibroblasts following a 5-day combination of kif and elig treatments, as evaluated by exclusion of the cell-impermeable far-red DNA dye DRAQ7. Triton X-100 (Triton; 0.02%) was added as a positive control for permeabilization. (**H**) Analysis of normal human fibroblast morphology following 5-day combinations of kif and elig treatments, assessed by live DIC microscopy. Cell-permeable Hoechst 33342 was included to visualize nuclei (blue). A slice of a Z-stack is shown based on nuclear stain focus. White scale bars indicate 50 μm. (**I**) Analysis of human fibroblast morphology following acute depletion and add-back of membrane cholesterol, assessed by live DIC microscopy. Cell-permeable Hoechst 33342 was included to visualize nuclei (blue). A slice of a Z-stack is shown based on nuclear stain focus. White scale bars indicate 50 μm. (**J**) Cell viability and membrane integrity of human fibroblasts following acute depletion and add-back of membrane cholesterol, as evaluated by exclusion of the cell-impermeable far-red DNA dye DRAQ7. Triton X-100 (Triton; 0.02%) was added as a positive control for permeabilization.

Treating replicating human fibroblasts for three days with kif led to loss of complex N-glycans, evident as increased detection of immature and oligomannose N-glycans by Concanavalin A (ConA) staining (Fig. EV2A). BAG treatment had no effect on ConA staining, but did impair Wheat Germ Agglutinin (WGA) staining, consistent with decreased abundance of GlcNAc and Neu5Ac due to inhibition of O-glycan initiation (Fig. EV2B). These treatments did not affect host cell morphology or viability (Fig. EV2C,D). Kif treatment of host cells significantly inhibited rhoptry discharge, while treatment with BAG had no effect (Fig. 2B). Interestingly, combining BAG and kif led to a moderate rescue of rhoptry discharge compared to kif alone. Since N- and O-glycosylation utilize a shared pool of precursor sugars and enzymes, kif and BAG can alter metabolic flux through these parallel biosynthetic pathways. BAG may therefore promote increased production of N-glycosylation, decreasing the inhibitory impact of kif (Kong and Fujita, 2025; McDonald et al, 2016; Scheper et al, 2023).

Treating dividing human fibroblasts with the UGCG inhibitor elig for five days lowered levels of the glycosphingolipid ganglioside GM1, measured by staining cells with fluorescently conjugated Cholera toxin subunit B (CTB; Fig. EV2E). A complementary treatment with kif led to the expected increase in ConA staining without affecting GM1 levels (Fig. EV2F). Neither kif nor elig altered host cell viability or morphology (Fig. EV2G,H). Elig treatment had a negligible effect on rhoptry discharge, compared to kif treatment for the same time period, further demonstrating the specific importance of N-glycans (Fig. 2C). Because kif prevents new complex N-glycans from forming, its effect depends on membrane protein turnover as fibroblasts grow. As expected, host cells became increasingly refractory to rhoptry discharge and invasion with prolonged kif treatment, plateauing by five days (Fig. 2D). Kif treatment impaired parasite invasion with similar kinetics. Taken together, these results point to N-glycans as the primary surface glycans required for normal rhoptry discharge ahead of invasion.

Host cell cholesterol has previously been found to be important for parasite replication and invasion (Besteiro et al, 2008; Coppens and Joiner, 2003; Fan et al, 2024; Tahara et al, 2016). Depletion of cholesterol from host cells blocks formation of evacuoles, a well-established readout of rhoptry discharge (Tahara et al, 2016; Håkansson et al, 2001). Host cholesterol also accumulates at the site of invasion (Besteiro et al, 2008), consistent with its involvement in invasion. To evaluate the role of cholesterol in rhoptry discharge, we extracted host cell membrane cholesterol using the cyclodextrin MβCD in serum-free media (Mahammad and Parmryd, 2015; Tahara et al, 2016). To exclude pleiotropic effects in cell signaling or viability, we reconstituted host cell plasma membrane cholesterol at varying levels (Fig. 2E). Regardless of treatment, host cells showed consistent morphology and viability (Fig. EV2I,J). Rhoptry discharge was strongly inhibited by cholesterol depletion from host cells and completely rescued at the highest levels of reconstitution (Fig. 2F). Invasion of parasites into host cells acutely depleted of cholesterol was also impaired, with cholesterol reconstitution leading to recovery of parasite invasion, mirroring the trends in rhoptry discharge (Fig. 2G). Together, this shows that in addition to galactose-containing N-glycans, cholesterol is required for rhoptry discharge and parasite invasion.

### Galactose-containing N-glycans are required for rhoptry discharge and invasion

**Figure 3:**
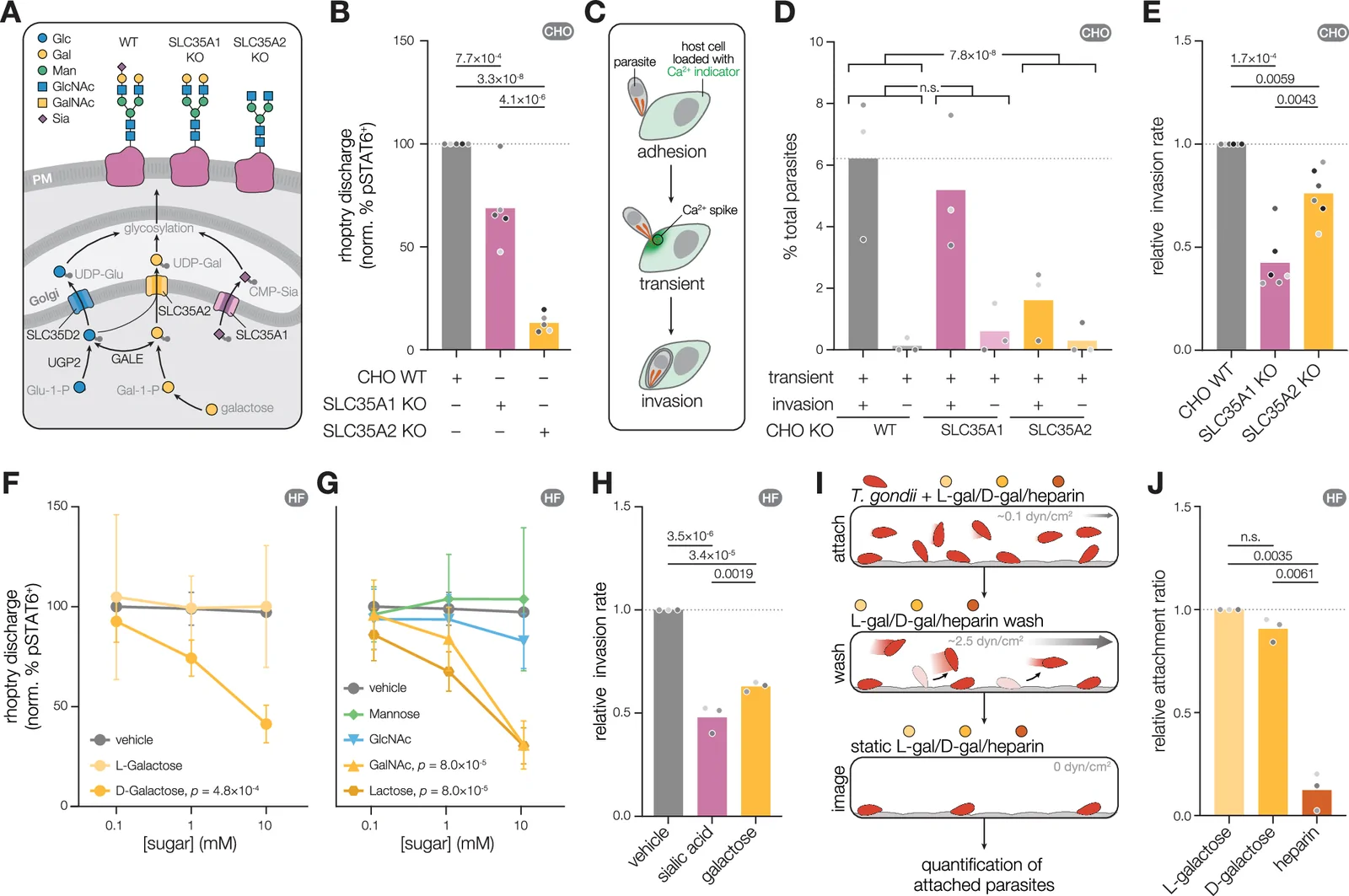
Recognition of host galactosylation on N-glycans is required for rhoptry discharge. (**A**) Simplified schematic of synthesis, transport, and utilization of UDP-Glc, UDP-Gal, and CMP-Sia precursors in mammalian N-glycosylation. (**B**) Rhoptry discharge into CHO cells with mutants disrupting sialic acid (SLC35A1 KO) and galactose (SLC35A2 KO) incorporation into glycosylated molecules. Host cells were challenged with wild-type parasites, and rhoptry discharge was assessed by the relative percent of pSTAT6-positive nuclei. Mean of *n* = 5 biological replicates is plotted; *P* values derived from one-way ANOVA with Tukey’s post hoc test. (**C**) Measurement of host cell transient perforation and parasite invasion. In a microfluidic microscopy slide, parasites adhere to host cells loaded with the Ca^2+^ indicator Cal-520. Upon rhoptry discharge, transient host cell perforation allows Ca^2+^ influx into the host cytosol, increasing Cal-520 fluorescence. Parasites are then individually tracked to identify those that invade subsequently to permeabilize their host cell. (**D**) Perforation and invasion of glycosylation-mutant CHO cells by wild-type parasites. Mean of *n* = 3 biological replicates is plotted; *P* values are derived from Fisher’s exact test. (**E**) Parasite invasion of glycosylation-mutant CHO cells. Host cells were challenged with wild-type dTomato-expressing parasites, and the invasion rate was assessed by counting the number of intracellular parasites as a proportion of host-cell nuclei. Mean of *n* = 3 biological replicates is plotted. *P* values derived from one-way ANOVA with Fisher’s LSD post hoc test. (**F**, **G**) Rhoptry discharge in the presence of varying amounts of galactose stereoisomers (**F**) or galactose-containing glycans (**G**). Rhoptry discharge was assessed by the relative percent of pSTAT6-positive nuclei after challenge with wild-type parasites. Each replicate was normalized to the vehicle control equivalent to the 0.1 mM condition. *P* values for the comparison to the vehicle control at 10 mM concentration are provided when significant, derived from two-way ANOVA testing with multiple comparison FDR correction using the Benjamini, Krieger, and Yekutieli two-stage step-up method. Mean of *n* = 3 biological replicates is plotted with error bars indicating s.d. Data from both panels are analyzed together in the same statistical model. (**H**) Parasite invasion rates into human fibroblasts in the presence of vehicle (PBS), galactose (G) or sialic acid (S). Host cells were challenged with wild-type dTomato-expressing parasites, and invasion rates were assessed by counting the number of intracellular parasites as a proportion of host-cell nuclei. Mean of *n* = 3 biological replicates is plotted; *P* values derived from one-way ANOVA with Fisher’s LSD post-hoc test. (**I**) Parasite attachment in the presence of L-galactose, D-galactose, or heparin sulfate. Attachment is quantified by counting the number of parasites adhered to fixed host cells in the flow chamber following attachment and wash steps. (**J**) Attachment rates of parasites to human fibroblasts in the presence of L-galactose (10 mM), D-galactose (10 mM), or heparin sulfate (10 mg/mL). Mean of *n* = 3 biological replicates is plotted. *P* values derived from one-way ANOVA with Fisher’s LSD post hoc test. Source data are available online for this figure.

**Figure EV3:**
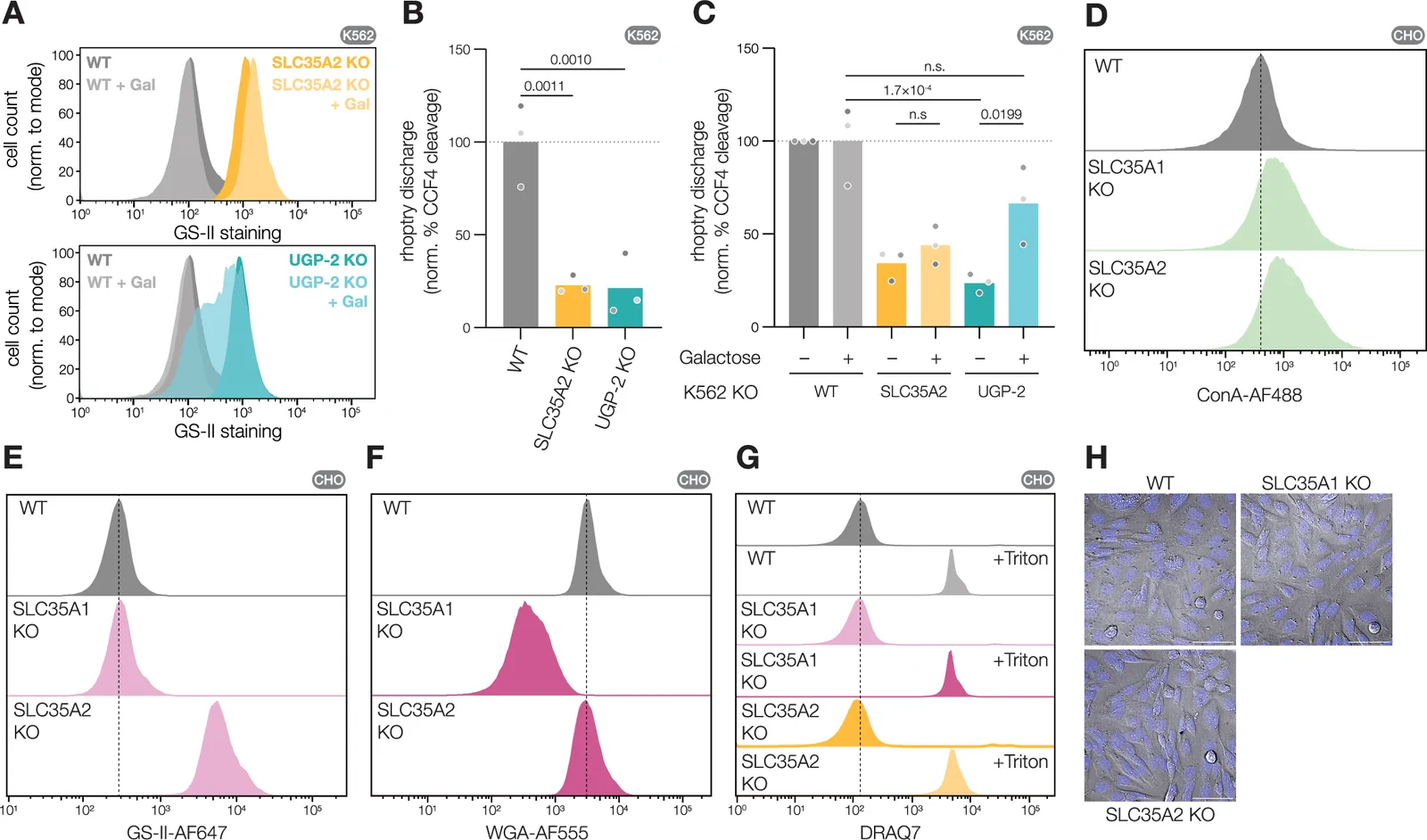
Validation of host cells defective in pathways related to glycosylation. (**A**) Flow cytometry of K562 cell lines in which UGP2 or SLC35A2 has been knocked out (KO). Cells were labeled with GS-II, which recognizes uncapped surface GlcNAc. Galactose supplementation (25 mM, 3 days) rescues surface glycosylation patterns for UGP2 KO but not SLC35A2 KO cells. (**B**) Rhoptry discharge was assessed in the KO cell lines as the percentage of host cells with βla-cleaved CCF4 relative to WT host cells, following challenge with rapamycin-treated DiCre/MyoH cKD/Txf-βla parasites. Mean of *n* = 3 biological replicates is plotted. *P* values derived from one-way ANOVA with Fisher’s LSD post hoc test. (**C**) Rhoptry discharge into host KO cell lines grown in the presence of vehicle or galactose (25 mM, 3 days) was assessed as the percentage of host cells with βla-cleaved CCF4 relative to vehicle-treated WT K562 cells, following challenge with rapamycin-treated DiCre/MyoH cKD/Txf-βla parasites. Mean of *n* = 3 biological replicates is plotted. *P* values derived from one-way ANOVA with Tukey’s post hoc test. (**D**–**F**) Verification of CHO cell mutants' surface glycosylation profiles by lectin-labeling and flow cytometry. CHO cells were stained with the exposed mannose-binding lectin ConA conjugated to Alexa Fluor 488 (ConA-AF488; **D**), with the terminal GlcNac-binding GS-II conjugated to Alexa Fluor 647 (GS-II-647; **E**), and with GlcNAc and Neu5Ac (sialic acid) binding WGA conjugated to Alexa Fluor 555 (WGA-AF555; **F**). (**G**) Cell viability and membrane integrity of wild type, galactosylation-deficient (SLC35A2 KO), and sialylation-deficient (SLC35A1 KO) CHO cell mutants, as evaluated by exclusion of the cell-impermeable far-red DNA dye DRAQ7. Triton X-100 (Triton; 0.02%) was added as a positive control for permeabilization. (**H**) Analysis of wild-type and mutant CHO cell morphology, assessed by live DIC microscopy. Cell-permeable Hoechst 33342 was included to visualize nuclei (blue). A slice of a Z-stack is shown based on nuclear stain focus. White scale bars indicate 50 μm.

N-glycans are built from several monosaccharides with variable linkage conformations and capping residues (Schjoldager et al, 2020; Otaki et al, 2022; Wallace et al, 2024). SLC35A2 and UGP2—required for galactosylation and glucosylation of N-glycans, respectively (Wolfe et al, 2021; Wang et al, 2020; Drabavicius and Daelemans, 2021; Kim et al, 2021)—were among the top four host genes with the strongest effects on rhoptry discharge. SLC35A2 transports UDP-Galactose (UDP-Gal) into the Golgi, where it provides the monosaccharide for growing glycan chains. UGP2 is involved in the synthesis of UDP-Glucose (UDP-Glc; Fig. 3A). UDP-Glc is also the main source of UDP-Gal under standard glucose-rich cell culture conditions through interconversion of the respective precursors by the enzyme GALE (Fig. 3A) (Drabavicius and Daelemans, 2021; Kim et al, 2021; Führing et al, 2015). We generated knockouts of UGP2 and SLC35A2 in human K562 cells and confirmed that both mutant cell lines showed robust staining with GS-II lectin, which binds terminal GlcNAc moieties exposed on glycans lacking capping galactosylation (Fig. EV3A) (Wang et al, 2020). Knockout of either factor significantly impaired rhoptry discharge (Fig. EV3B). As expected, due to GALE-mediated interconversion, growth for three days in the presence of galactose partially restored galactosylation in UGP2 KO but not SLC35A2 KO cells, since cells lacking the transporter are unable to import UDP-Gal into the Golgi (Fig. EV3A). Rhoptry discharge was partially restored by growing UGP2 KO host cells in galactose, but no such rescue was observed for the SLC35A2 KO (Fig. EV3C). These experiments indicate that galactose-containing N-glycans are critical for host cell susceptibility to rhoptry discharge.

To further study the relationship between N-glycan composition and rhoptry discharge, we turned to Chinese Hamster Ovary (CHO) cells, for which characterized mutants are available that disrupt CMP-sialic acid (also known as CMP-Neu5Ac) Golgi transport via SLC35A1 (CHO Lec2) and UDP-Gal Golgi transport via SLC35A2 (CHO Lec8) (Patnaik and Stanley, 2006; Esko et al, 2022). These cell lines had the expected surface glycosylation patterns (Fig. EV3D–F) and displayed consistent viability and morphology (Fig. EV3G,H), providing a set of adherent cell lines in a second species to probe the role of host glycosylation in *T. gondii* rhoptry discharge. Consistent with findings in K562 cells, SLC35A2 disruption in CHO cells reduced rhoptry discharge (Fig. 3B). By contrast, removal of sialic acid by SLC35A1 disruption had only a moderate impact on rhoptry discharge.

As an alternative approach to measuring rhoptry discharge, we examined transient host cell permeabilization during *T. gondii* infection. Recent work has demonstrated that parasites transiently perforate the host cell membrane prior to invasion in a manner that depends on rhoptry discharge factors like the CLAMP complex (Male et al, 2025; Kegawa et al, 2025). To examine if host cell membrane perforation is also dependent on host galactosylation, we measured *Toxoplasma-*induced CHO cell perforation by imaging Ca^2+^ transients in host cells during invasion (Fig. 3C) (Male et al, 2025). Consistent with a crucial role for host galactosylation in the initiation of rhoptry discharge, we observed fewer permeabilization events in SLC35A2 KO cells, and this coincided with fewer invasion events. By contrast, SLC35A1 KO host cells, deficient in sialylation, were perforated and invaded at similar rates to wild-type (Fig. 3D). Host cell perforation requires rhoptry discharge (Male et al, 2025), and likely precedes the protein translocation required for a positive Txf-βla and phospho-STAT6 signal. The reduction in the frequency of perforation from loss of galactose (SLC35A2) is therefore consistent with host galactosylation of N-glycans being required early in the process of rhoptry discharge.

Intriguingly, invasion in the perforation assays differed from previous work, which showed a greater invasion defect when SLC35A1 is knocked out compared to SLC35A2 knockout (Monteiro et al, 1998). However, the perforation assay allows parasites to adhere before a buffer swap removes unattached parasites, synchronizes invasion, and records for a much shorter assay window than conventional invasion assays. Using a standard invasion assay in which parasite attachment is a component of the readout, we confirmed the previously reported trend, showing both SLC35A1 and SLC35A2 knockouts significantly affect invasion, but the SLC35A1 KO has the greater effect (Fig. 3E). Notably, SLC35A1 KO CHO cells have previously been reported to affect *T. gondii* adhesion (Baba et al, 2015), suggesting that sialic acids are important for general attachment and invasion, while efficient rhoptry discharge relies primarily on host galactosylation.

Many pathogens bind host glycans, and their interactions can be competitively disrupted by adding exogenous glycans in culture or in vivo (Bucior et al, 2013; Poole et al, 2018). For example, *T. gondii* adhesion and invasion can be reduced by incubation with heparan and sialic acid (Carruthers et al, 2000; Valleau et al, 2023; Friedrich et al, 2010; Marchant et al, 2012). We tested whether exogenous galactose could competitively disrupt rhoptry discharge. When added together with parasites (i.e., without prior incubation) D-galactose significantly inhibited rhoptry discharge in a dose-dependent manner (Fig. 3F). By contrast, the enantiomer L-galactose had no effect, excluding non-specific effects on the osmolarity or composition of the media. Structurally related sugars like GalNAc and the galactose-containing disaccharide lactose similarly inhibited rhoptry discharge, while GlcNAc and mannose had no effect (Fig. 3G). We confirmed that blocking rhoptry discharge with exogenous galactose had the expected inhibitory effect on parasite invasion, as did free sialic acid (Fig. 3H). These results mirror the effects of SLC35A1 and SLC35A2 KO, suggesting that exogenous galactose and related sugars competitively inhibit parasite binding of galactose-containing host N-glycans.

Because rhoptry discharge and host cell protein delivery occur downstream of attachment, we used a quantitative flow-based adhesion assay to evaluate this initial interaction with host cells. In this assay, parasites are flowed over a formaldehyde-fixed confluent host cell monolayer and allowed to adhere to host cells at a low flow rate. A stringent wash is then used to remove weakly interacting and non-attached parasites (Fig. 3I). Despite its effect on rhoptry discharge and invasion, addition of exogenous D-galactose did not alter parasite attachment to human fibroblasts, relative to its enantiomer L-galactose (Fig. 3J). By contrast, addition of heparin dramatically inhibited parasite attachment, consistent with prior work (Carruthers et al, 2000). Collectively, these results show that galactose-containing N-glycans mediate rhoptry discharge downstream of parasite attachment.

### Host N-glycosylation and cholesterol are required for rhoptry discharge independently of the CLAMP and CRMP complexes

**Figure 4:**
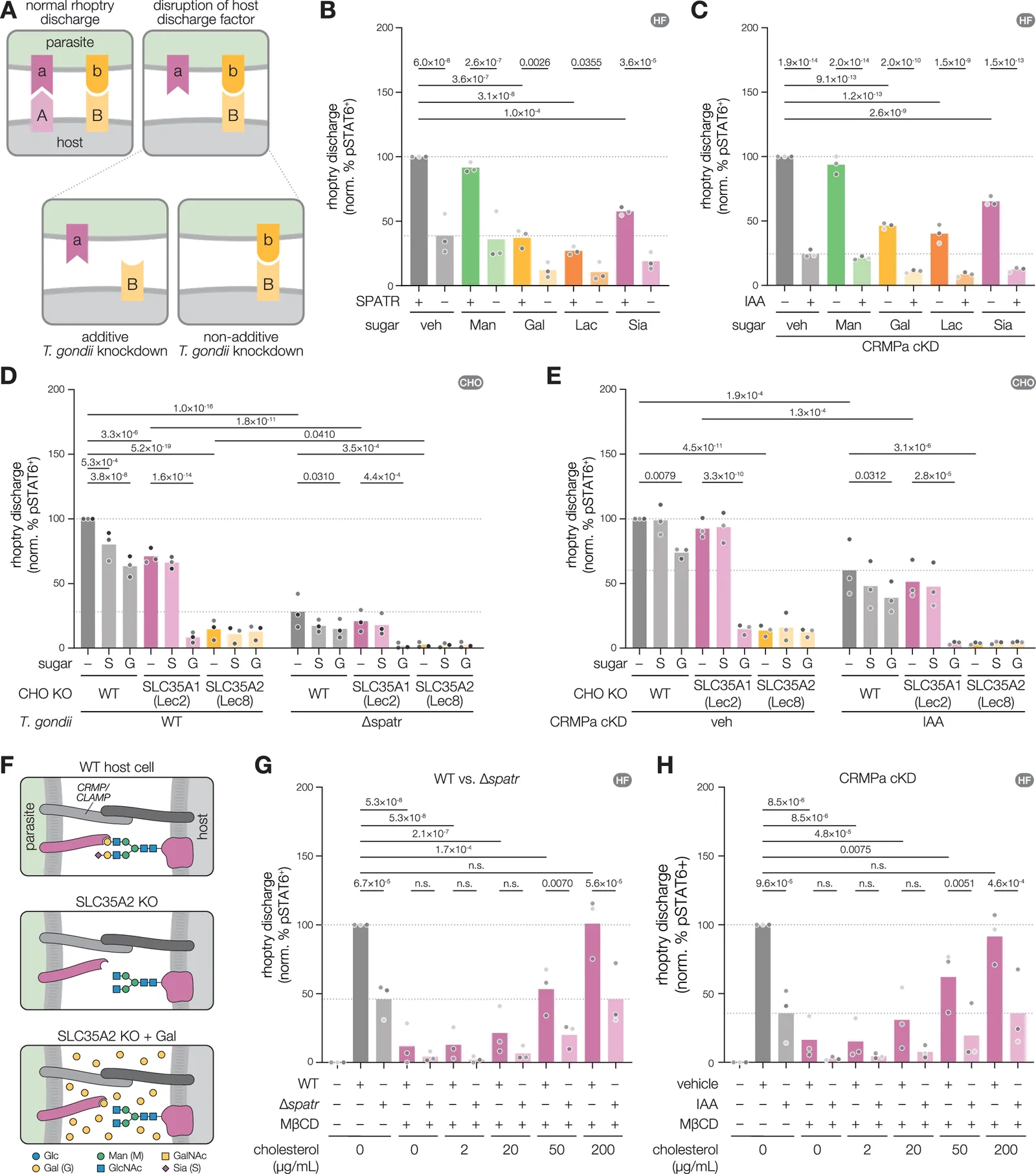
The CRMP and CLAMP complexes are not involved in binding galactosyl N-glycans or cholesterol during rhoptry discharge. (**A**) Scheme to determine whether host and parasite factors participate in common or parallel interaction pathways. An additive effect is expected from host and parasite perturbations disrupting different pathways, whereas non-additive interactions result from them disrupting a common pathway. (**B**, **C**) Individual and combined effects of exogenous sugars and genetic disruption of the CLAMP complex (**B**) or the CRMP complex (**C**). In all, 10 mM of the indicated sugar was added with the parasites (Man mannose, Gal galactose, Lac lactose, Sia sialic acid). Human fibroblasts were incubated with wild-type (SPATR +) or Δ*spatr* parasites (SPATR –) (**B**), or CRMPa cKD parasites grown in the presence of IAA or vehicle control (**C**). Rhoptry discharge was assessed by the relative percent of pSTAT6-positive nuclei. Mean of *n* = 3 biological replicates is plotted. *P* values for each experiment derived from two-way ANOVA with Tukey’s post hoc multiple comparison test. (**D**, **E**) Individual or combined effects of genetic perturbation of host and parasite pathways, together with exogenous sugars. Wild-type CHO cells or mutants in glycosylation pathways (SLC35A1 and SLC35A2 KOs) were challenged with parasites disrupted in CLAMP (**D**) or CRMP (**E**) complexes, as above. 10 mM of each indicated exogenous sugar was added with the parasites (G galactose, S sialic acid). Rhoptry discharge was assessed by the relative percent of pSTAT6-positive nuclei. Mean of *n* = 3 biological replicates is plotted. *P* values are derived from two-way ANOVA testing with multiple comparison FDR correction using the Benjamini, Krieger, and Yekutieli two-stage step-up method. For both (**D**, **E**), data were analyzed as part of the same datasets as Fig. EV4B,C, respectively. (**F**) Model explaining the non-additive effect of combining exogenous galactose and SLC35A2 KO, implying that the effect of host galactosylation is independent of the pathways regulated by CLAMP and CRMP complexes. (**G**, **H**) Rhoptry discharge was measured on cholesterol-depleted or reconstituted human fibroblasts. Rhoptry discharge was measured as the relative percent of pSTAT6-positive nuclei after incubation with Δ*spatr* (–) or wild-type parental (+) parasites (**G**), or a CRMPa conditional knockdown strain treated with vehicle or IAA (**H**). Mean of *n* = 3 biological replicates is plotted; *P* values are derived from two-way ANOVA testing with multiple comparison FDR correction using the Benjamini, Krieger, and Yekutieli two-stage step-up method. Source data are available online for this figure.

The CRMP and CLAMP complexes are conserved among apicomplexans, released from micronemes, and required for rhoptry discharge (Singer et al, 2023; Sparvoli et al, 2022; Valleau et al, 2023). We examined whether host N-glycans or cholesterol mediate the roles of these parasite complexes in rhoptry discharge. We reasoned that interactions between host and parasite factors could be evaluated by examining how parasites deficient in specific rhoptry discharge factors (e.g., CLAMP or CRMP complex knockdowns) respond to the simultaneous disruption of candidate host factors. In this approach, simultaneous inhibition of host and parasite factors on the same pathway should reduce rhoptry discharge to the same extent as inhibiting either component alone, resulting in a non-additive effect. By contrast, if the dually targeted parasite and host factors play independent roles in rhoptry discharge, we should see an additive effect during simultaneous inhibition, as we would be disrupting multiple host-parasite interactions simultaneously (Fig. 4A).

**Figure EV4:**
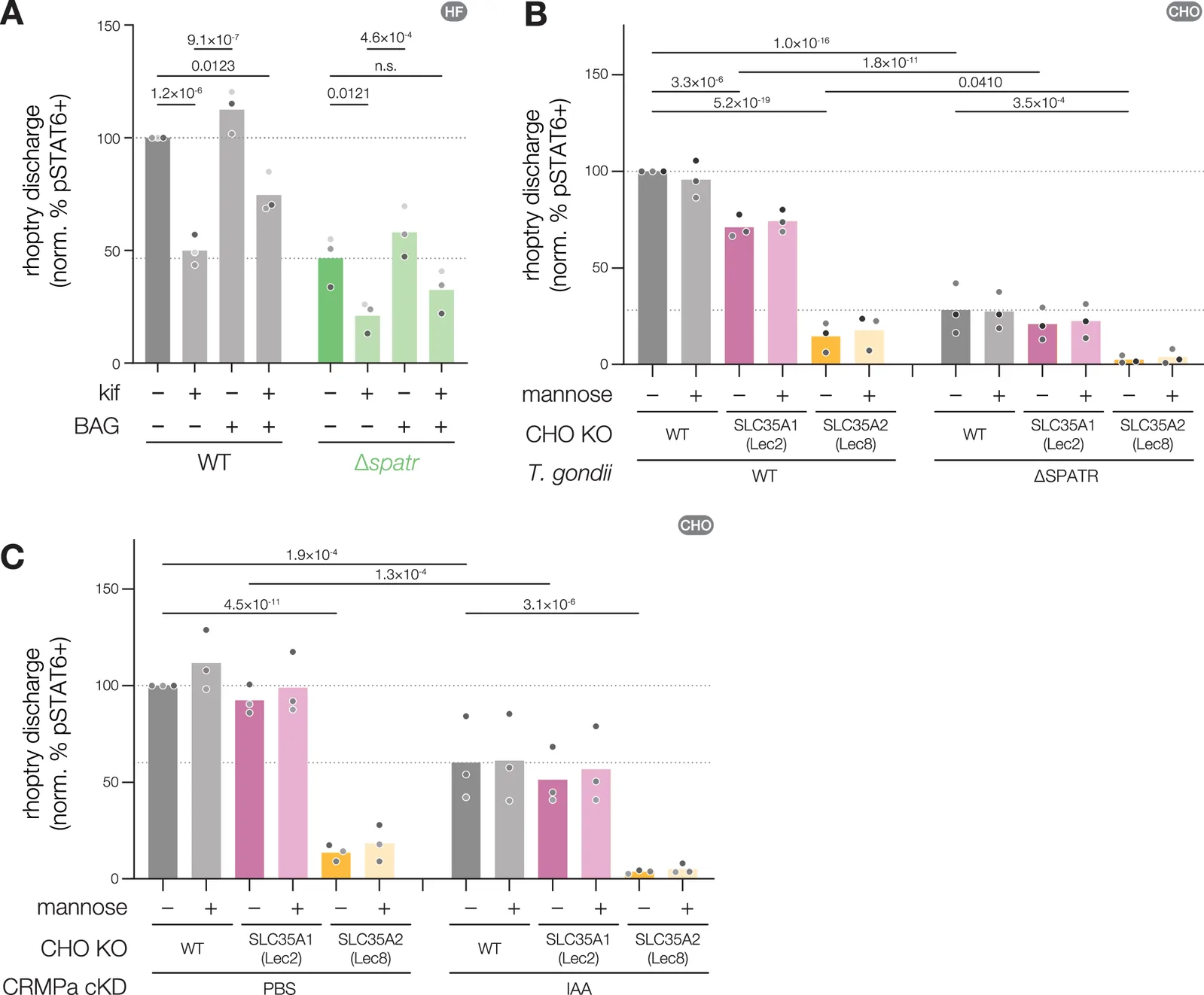
Supporting data demonstrating that CLAMP and CRMP complexes additively interact with galactose and N-glycans. (**A**) The interaction of the CLAMP complex with N-glycans and O-glycans in rhoptry discharge was assessed by treating human fibroblasts for 3 days with kif (10 μM) or BAG (2 mM) then incubating with Δ*spatr* or wild-type parental parasites. Rhoptry discharge was assessed by the relative percent of pSTAT6-positive nuclei. Mean of *n* = 3 biological replicates is plotted. *P* values were derived from two-way ANOVA with Tukey’s post hoc multiple comparison test. Note that the values for the wild-type parasite condition are the same data as those in Fig. 2B. (**B**, **C**) Evaluation of the interaction of CHO galactose (SLC35A2 KO) and sialic acid (SLC35A1 KO) knockouts combined with mannose addition against CLAMP (**A**) or CRMP (**B**) complexes in rhoptry discharge. Host cells were challenged with the indicated CLAMP perturbation (wild-type or Δ*spatr* parasites; **A**) or CRMP perturbation (CRMPa cKD treated with IAA or PBS vehicle; **B**) in the presence of 10 mM of mannose. Rhoptry discharge was assessed by the relative percent of pSTAT6-positive nuclei. Mean of *n* = 3 biological replicates is plotted. *P* values are derived from two-way ANOVA testing with multiple comparison FDR correction using the Benjamini, Krieger, and Yekutieli two-stage step-up method. For both (**B**, **C**), data were analyzed as part of the same datasets as Fig. 4D and E, respectively.

We first examined the interaction between exogenous sugars and the CRMP and CLAMP complexes. To disrupt the CLAMP and CRMP complexes, we used a SPATR knockout (Δ*spatr*) and an auxin-inducible CRMPa knockdown (CRMPa-mAID), respectively (Huynh et al, 2014; Valleau et al, 2023; Sparvoli et al, 2022; Singer et al, 2023). These perturbations result in partial inhibition of rhoptry discharge, allowing for accurate measurements of additivity when coupled with the exogenous sugars. As expected, mannose had no effect on rhoptry discharge. By contrast, exogenous galactose and lactose robustly impaired rhoptry discharge into human fibroblasts; however, this effect was additive with the disruption of either the CLAMP (Δ*spatr*) or CRMP (CRMPa cKD) complexes (Fig. 4B,C). Exogenous sialic acid modestly inhibited rhoptry discharge—consistent with the CHO cell SLC35A1 disruption results—but the effect also appeared additive, and therefore independent, of the CRMP or CLAMP complexes (Fig. 4B,C). A similar additive interaction could also be observed between Δ*spatr* parasites and the global disruption of N-glycosylation with kif (Fig. EV4A).

We examined the additivity between host galactosylation, exogenous sugars, and the parasite complexes by performing pSTAT6 assays in the CHO knockout cells. This confirmed that the impaired rhoptry discharge into SLC35A2 KO cells (lacking surface galactosylation) could not be further inhibited by exogenous galactose, despite being additively inhibited by disruption of the CLAMP or CRMP complexes (Fig. 4D,E). Interestingly, galactose and sialic acid suppressed rhoptry discharge more modestly in CHO cells compared to human fibroblasts, suggesting that the balance of glycan factors influencing rhoptry discharge may differ between cell types or host species. Nevertheless, exogenous galactose strongly blocked rhoptry discharge in SLC35A1 KO cells, whose complex N-glycans predominantly terminate in galactose and lack capping sialic acids (Fig. 4D,E). As expected, sialic acid had no effect on rhoptry discharge into SLC35A1 KO cells. As with human fibroblasts, mannose had no effect on rhoptry discharge into any of the CHO cell lines (Fig. EV4B,C). These results suggest other parasite factors likely recognize galactose-containing N-glycans during rhoptry discharge (Fig. 4F).

We similarly evaluated whether the CRMP and CLAMP complexes interface with host cholesterol. As above, rhoptry discharge was strongly inhibited by MβCD-mediated cholesterol depletion and rescued by reconstitution. However, we observed additive effects between the CRMP and CLAMP complex perturbations and all levels of host membrane cholesterol reduction in human fibroblasts (Fig. 4G,H). These results demonstrate that the CLAMP and CRMP complexes contribute to rhoptry discharge independently of host N-glycosylation or membrane cholesterol.

### The MIC1/4/6 complex interacts with galactose-containing glycans

**Figure 5:**
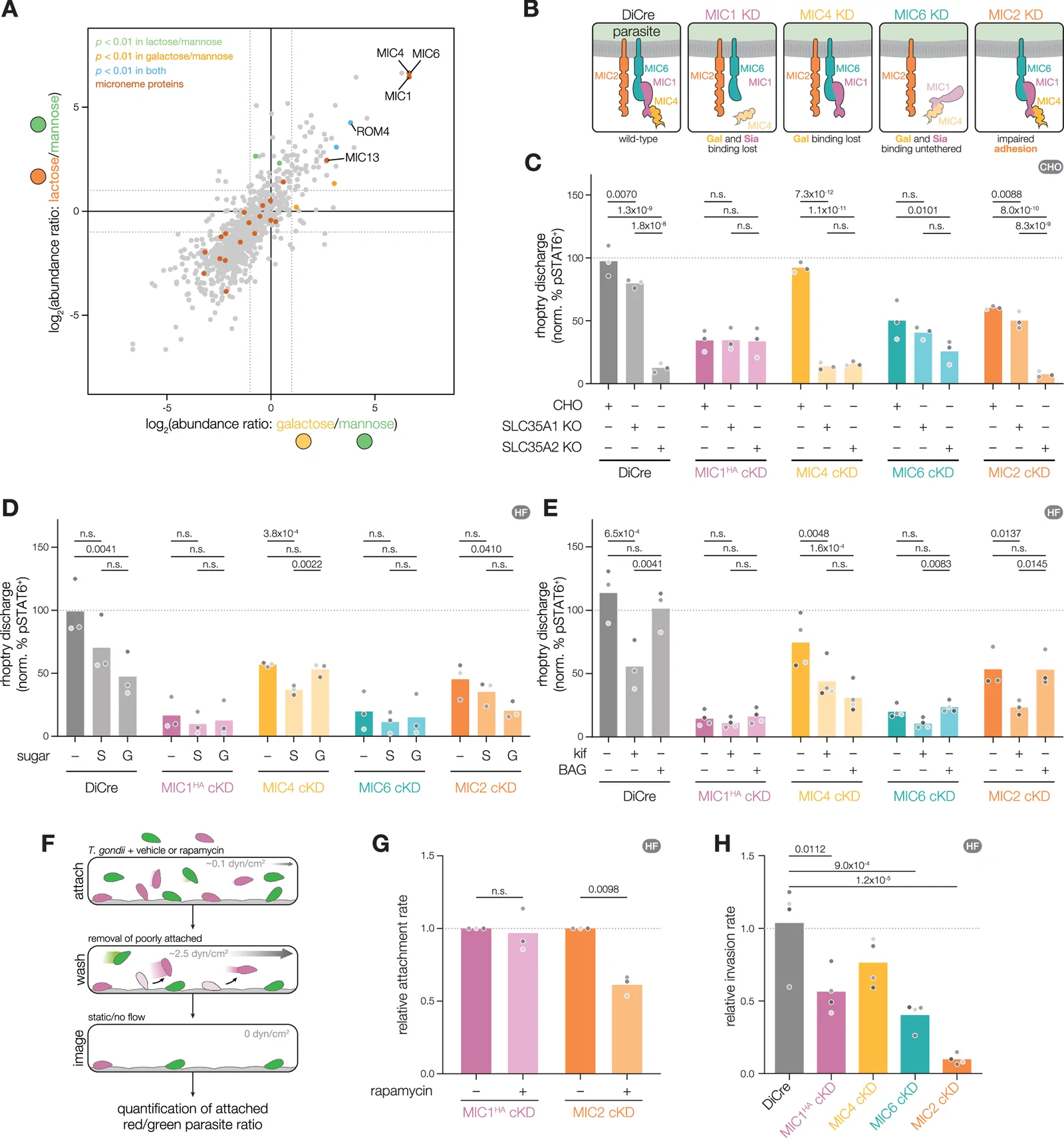
The MIC1/4/6 complex drives interaction with galactose- and sialic acid–containing N-glycans during rhoptry discharge. (**A**) Proteins from *T. gondii* lysates were differentially enriched using galactose-, lactose-, or mannose-coated agarose beads, and their abundance and identity were determined by mass spectrometry. Mannose beads were used as the negative control for background subtraction. Dashed lines indicate a relative twofold enrichment. Mean of *n* = 2 biological replicates is plotted. *P* values are derived from Benjamini–Hochberg adjusted *t* tests and only displayed for proteins with enrichment ratios >2 in the indicated dimension. Known microneme proteins are highlighted (red). (**B**) Models of the expected parasite perturbations following knockdown in the panel of strains used below. For simplicity, this diagram does not display the previously established stoichiometry of the MIC1/4/6 complex (Sawmynaden et al, 2008; Marchant et al, 2012). (**C**) Rhoptry discharge into wild-type or glycosylation-mutant CHO cell lines by the panel of rapamycin-treated wild-type (DiCre) or cKD strains. Rhoptry discharge was assessed by the relative percent of pSTAT6-positive nuclei. Mean of *n* = 3 biological replicates is plotted. *P* values derived from analysis of each parasite strain separately (vehicle and knockdown conditions) using two-way ANOVA with Tukey’s post hoc multiple comparison test. (**D**) Inhibition of rhoptry discharge by exogenous sugars. The panel of rapamycin-treated strains was incubated with human fibroblasts in the presence of vehicle (–) or 10 mM of the indicated sugar (G galactose, S sialic acid). Rhoptry discharge was assessed by the relative percent of pSTAT6-positive nuclei. Mean of *n* = 3 biological replicates is plotted. *P* values derived from analysis of parasite in each strain separately (vehicle and knockdown conditions) using two-way ANOVA with Tukey’s post hoc multiple comparison test. (**E**) Inhibition of rhoptry discharge by inhibition of host N- or O-glycosylation by pretreating human fibroblasts for 4 days with kif (10 μM) or BAG (2 mM), respectively. Rhoptry discharge was assessed by the relative percent of pSTAT6-positive nuclei following incubation of host cells with wild-type (DiCre) or cKD strains. Mean of *n* = 3–4 biological replicates is plotted. *P* values derived from analysis of each parasite strain separately (vehicle and knockdown conditions) using two-way ANOVA with Tukey’s post hoc multiple comparison test. (**F**) Parasite attachment following microneme protein knockdown. For each mNeonGreen-expressing cKD parasite strain, either vehicle or knockdown (rapamycin) condition was stained with CellTracker Red CMTPX, the treatments were combined in equal proportions and flowed onto a formaldehyde-fixed monolayer of human fibroblasts in a microfluidic chamber at ~0.1 dyn/cm^2^ for 12 min. Non-adherent and weakly attached parasites were then removed with a 2-min high-shear wash (~ 2.5 dyn/cm^2^). Relative attachment of knockdown and vehicle-treated parasites was assessed based on the number of remaining parasites of each type. (**G**) Attachment of rapamycin- and vehicle-treated MIC1^HA^ and MIC2 cKD parasite strains. Mean of *n* = 3 biological replicates is plotted. *P* values derived from one-sample *t* tests to the normalized attachment rate of 1. (**H**) Invasion of human fibroblasts by wild-type (DiCre) or cKD strains. Invasion rates were assessed by counting the number of internalized parasites per host cell nucleus. Mean of *n* = 4 biological replicates is plotted. *P* values derived from one-way ANOVA with Tukey’s post hoc multiple comparison test. Source data are available online for this figure.

Our results suggest that the conserved CRMP and CLAMP complexes are not responsible for the recognition of galactose-containing N-glycans on the host cell. Therefore, we sought to identify parasite proteins capable of binding galactose-containing glycans. Inspired by previous approaches used to discover lactose-binding parasite proteins (Pinzan et al, 2015; Lourenço et al, 2001; Sardinha-Silva et al, 2019), we flowed *T. gondii* lysate over agarose beads with conjugated galactose, lactose, or mannose. After stringent washing, we specifically eluted bound proteins by flowing high concentrations (> 100 mM) of the cognate sugars over each resin, and used mass spectrometry to identify bound parasite proteins. As mannose is not involved in rhoptry discharge (Fig. 3G), this condition serves as a negative control. MIC1, MIC4, and MIC6 were specifically enriched by lactose and galactose beads compared to mannose (Fig. 5A; Dataset EV2). This is consistent with previous studies that identified MIC1 and MIC4 by lactose-bead pull-downs and showed they form a heterotrimeric complex with MIC6 (Reiss et al, 2001; Lourenço et al, 2001). MIC4 is known to be a terminal galactose-binding lectin, likely responsible for facilitating binding of the MIC1/4/6 complex to lactose and galactose resins (Marchant et al, 2012). Intriguingly, the next most abundant protein was MIC13, which is a paralog of MIC1 (Friedrich et al, 2010). No other microneme proteins were significantly enriched, suggesting the MIC1/4/6 complex may be the major galactose-interacting microneme complex in *T. gondii*.

### The MIC1/4/6 complex binds host galactosyl N-glycans required for efficient rhoptry discharge

**Figure EV5:**
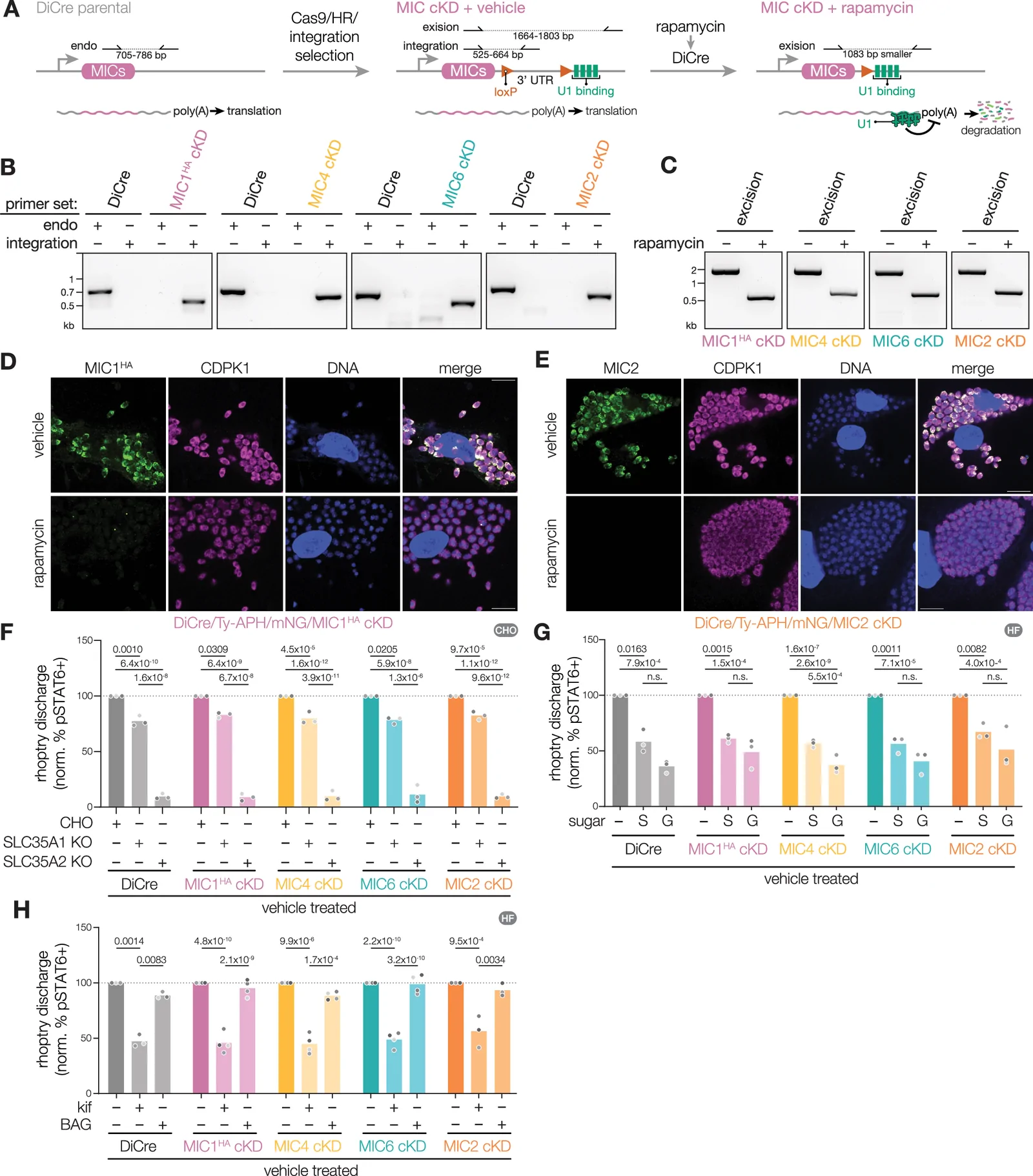
Strain validation and vehicle-treated conditions for MIC1/4/6 and MIC2 interactions with glycans in rhoptry discharge. (**A**) C-terminal tagging and a conditional DiCre/U1 knockdown approach were used for MIC cKD strain construction (MIC1^HA^ cKD, MIC4 cKD, MIC6 cKD, and MIC2 cKD). The endogenous 3′ UTR is replaced with a floxed 3′ UTR ahead of a tandem array of U1 motifs. Rapamycin treatment dimerizes DiCre, leading to 3′ UTR excision, replacement with the U1 array, and U1-mediated mRNA transcript degradation. PCRs used for endogenous loci, construct integration, and DiCre excision validations are indicated. (**B**) Agarose gels of the endogenous (endo) and construct integration PCRs demonstrating correct integration of the floxed 3′ UTRs for each cKD parasite strain. (**C**) Agarose gels demonstrating correct excision of the floxed 3′ UTR and appending of the tandem U1 array upon rapamycin treatment of each cKD parasite strain. (**D**, **E**) Evaluation of MIC1^HA^ (**D**) and MIC2 (**E**) microneme localization following rapamycin treatment of cKD strains. Parasites were treated for 2 h with rapamycin, then allowed to grow intracellularly for 48 h prior to fixation and staining. anti-CDPK1 was used as a *T. gondii* cytosolic control, and Hoechst 33258 was used to stain DNA. (**F**) Challenge of vehicle-treated DiCre, MIC1^HA^, MIC4, MIC6, and MIC2 cKD strains against WT, SLC35A1 KO, and SLC35A2 KO CHO cells. Control pSTAT6 assay conditions for rapamycin-induced knockdowns in Fig. 4C. Mean of *n* = 3 biological replicates is plotted. *P* values derived from analysis of each strain separately (vehicle and knockdown conditions) using two-way ANOVA with Tukey’s post hoc multiple comparison test. (**G**) Challenge of vehicle-treated DiCre, MIC1^HA^, MIC4, MIC6, and MIC2 cKD strains against human fibroblast in the presence of 10 mM of indicated sugar (galactose; G, sialic acid; S, or vehicle; –). Control pSTAT6 assay conditions for rapamycin-induced knockdowns in Fig. 4D. Mean of *n* = 3 biological replicates is plotted. *P* values derived from analysis of each strain separately (vehicle and knockdown conditions) using two-way ANOVA with Tukey’s post hoc multiple comparison test. (**H**) Challenge of vehicle-treated treatment DiCre, MIC1^HA^, MIC4, MIC6, and MIC2 cKD strains against human fibroblasts treated for 4 days with kif (10 μM) or BAG (2 mM). Control pSTAT6 assay conditions for rapamycin-induced knockdowns in Fig. 4E. Mean of *n* = 3 (DiCre and MIC2 cKD) or *n* = 4 (MIC1, MIC4, MIC6 cKDs) biological replicates is plotted. *P* values derived from analysis of each strain separately (vehicle and knockdown conditions) using two-way ANOVA with Tukey’s post hoc multiple comparison test.

Prior work found that MIC1 and MIC4 bind sialic acid and galactose, respectively (Friedrich et al, 2010; Marchant et al, 2012; Zhu et al, 2021), while the transmembrane domain–containing MIC6 anchors the complex to the parasite plasma membrane but lacks glycan-binding domains (Reiss et al, 2001). The MIC1/4/6 complex has been shown to contribute to invasion but not attachment (Cérède et al, 2005), a phenotype consistent with a role in rhoptry discharge (Possenti et al, 2022). To directly examine the role of these microneme proteins in rhoptry discharge, we generated a panel of conditional knockdown (cKD) strains using the U1 system in which the endogenous 3′ UTR of a target gene is replaced with a floxed 3′ UTR followed by four tandemly arrayed U1-binding sites (Pieperhoff et al, 2015). In the DiCre background (Hunt et al, 2019), a 2-h rapamycin treatment leads to excision of the 3′ UTR, blocking proper mRNA processing and expression of the target protein (Fig. EV5A). We generated cKD strains for MIC1, MIC4, and MIC6, as well as the control MIC2, which contributes to attachment but not rhoptry discharge (Huynh and Carruthers, 2006; Harper et al, 2004; Gras et al, 2017) (Fig. 5B). We verified correct integration of the cKD constructs for all strains by PCR (Fig. EV5B), and confirmed DiCre-driven 3′ UTR excision occurs as expected following rapamycin treatment (Fig. EV5C). Epitope tagging was avoided for MIC4 and MIC6 because we suspected it would interfere with their function. However, we included a C-terminal HA tag in the MIC1 cKD and could detect MIC2 based on an available antibody. Robust knockdown of MIC1 and MIC2 could therefore be observed by microscopy upon rapamycin treatment (Fig. EV5D,E).

Using the newly generated strains, we examined the genetic interactions between the disrupted host and parasite pathways. In the absence of rapamycin treatment, all cKD strains showed identical rhoptry discharge profiles across the CHO cell lines tested (Fig. EV5F). However, upon knockdown, we observed clear strain-specific effects. MIC2 knockdown showed additive effects with the loss of host sialylation (SLC35A1 KO) or galactosylation (SLC35A2 KO), indicating that MIC2’s host cell adhesion helps set the overall levels of rhoptry discharge but does not interact directly with these N-glycosylation pathways (Fig. 5C). By contrast, knockdown of MIC1 or MIC6 displayed clearly non-additive effects with the host mutations. Despite MIC4’s role in galactose binding, knockdown had a minimal effect on rhoptry discharge into wild-type CHO cells. However, MIC4 knockdown parasites were uniquely reliant on host sialylation, displaying similar, low-level rhoptry discharge into cell lines lacking SLC35A1 (no sialylation) or SLC35A2 (neither sialylation nor galactosylation). This contrasts with the minor impact of SLC35A1 KO on both wild-type and MIC2-knockdown parasites, implying redundancy between galactose and sialic acid binding. These results also corroborate MIC1/4/6 complex organization (Fig. 5B). For example, MIC1 mediates attachment of MIC4 to the complex, so loss of MIC1 or MIC6 should render parasites unable to bind either sialic acids (via MIC1) or galactose (via MIC4). By contrast, MIC1 and MIC6 still associate when MIC4 is lost (Friedrich et al, 2010; Reiss et al, 2001; Zhu et al, 2021; Saouros et al, 2005), so the complex can lose galactose binding while retaining sialic-acid binding.

We also examined the role of the MIC1/4/6 complex in rhoptry discharge into human fibroblasts, measuring the effect of exogenous sugars on this process. Again, rhoptry discharge was normal across all of the strains prior to rapamycin treatment, and was reduced by exogenous sialic acid or galactose (Fig. EV5G). As with the CHO mutants, MIC2 knockdown reduced rhoptry discharge and was additively inhibited by exogenous galactose (Fig. 5D). By contrast, loss of MIC1 or MIC6 strongly inhibited rhoptry discharge into human fibroblasts, with no additional effect from the addition of sialic acid or galactose. Consistent with the specific interaction between galactose and MIC4, MIC4 knockdown parasites were modestly affected by exogenous sialic acid but not galactose.

Recombinant MIC1 and MIC4 have been shown to bind N-glycans (Sardinha-Silva et al, 2019; Marchant et al, 2012; Friedrich et al, 2010). We examined whether the effect of the MIC1/4/6 complex on rhoptry discharge occurs through host N-glycans. Indeed, kif treatment, which reduces complex N-glycans, had no effect on the already suppressed rhoptry discharge of the MIC1 and MIC6 knockdowns, but had additive effects with knockdown of MIC2 or MIC4 (Figs. 5E and EV5H). These results are consistent with the complete loss of N-glycan binding when MIC1 or MIC6 is lost, but retention of sialic acid binding when MIC4 is disrupted (Fig. 4B). Intriguingly, BAG treatment only inhibited rhoptry discharge when MIC4 had been disrupted, suggesting either secondary binding of O-glycans by the MIC1/6 subcomplex or, more likely, suppression of NeuAcα2-3 linkages—the major MIC1-bound glycans—by the known off-target activity of BAG (Huet et al, 1998; Ulloa et al, 2000).

We further examined the contributions of the MIC1/4/6 complex on attachment and invasion. To sensitively detect the attachment defects from loss of single microneme proteins, we adapted our microfluidic flow-based attachment assay to simultaneously monitor vehicle and rapamycin-treated parasites, distinguished by distinct fluorescent labels (Fig. 5F). MIC2 knockdown resulted in the expected attachment defect to host cells, whereas knockdown of MIC1 had no effect (Fig. 5G). Our results are consistent with prior work suggesting MIC1 knockout parasites display normal attachment (Cérède et al, 2005). Since invasion requires both attachment and rhoptry discharge, we expected loss of both the MIC1/4/6 complex and MIC2 would impact this process. Indeed, loss of the MIC1/4/6 complex or MIC2 significantly reduced parasite invasion (Fig. 5H). Knockdown of MIC1 or MIC6 had a greater impact on invasion compared to knockdown of MIC4, in line with their relative impact on rhoptry discharge. Notably, loss of MIC2 had the largest impact on invasion, suggesting that MIC2 attachment and gliding motility are critical to achieve the high rates of invasion observed in these assays (Huynh and Carruthers, 2006; Harper et al, 2004; Gras et al, 2017). Furthermore, gliding motility—excluded from rhoptry discharge assays based on mycalolide B treatment—may partially compensate for the role of the MIC1/4/6 complex in rhoptry discharge.

Taken together, these results point to the MIC1/4/6 complex as the major mediator of host N-glycan recognition during rhoptry discharge, specifically through the redundant recognition of host sialylation through MIC1 and galactosylation through MIC4.

### The MIC1/4/6 complex drives cholesterol-dependent formation of membrane microdomains

**Figure 6:**
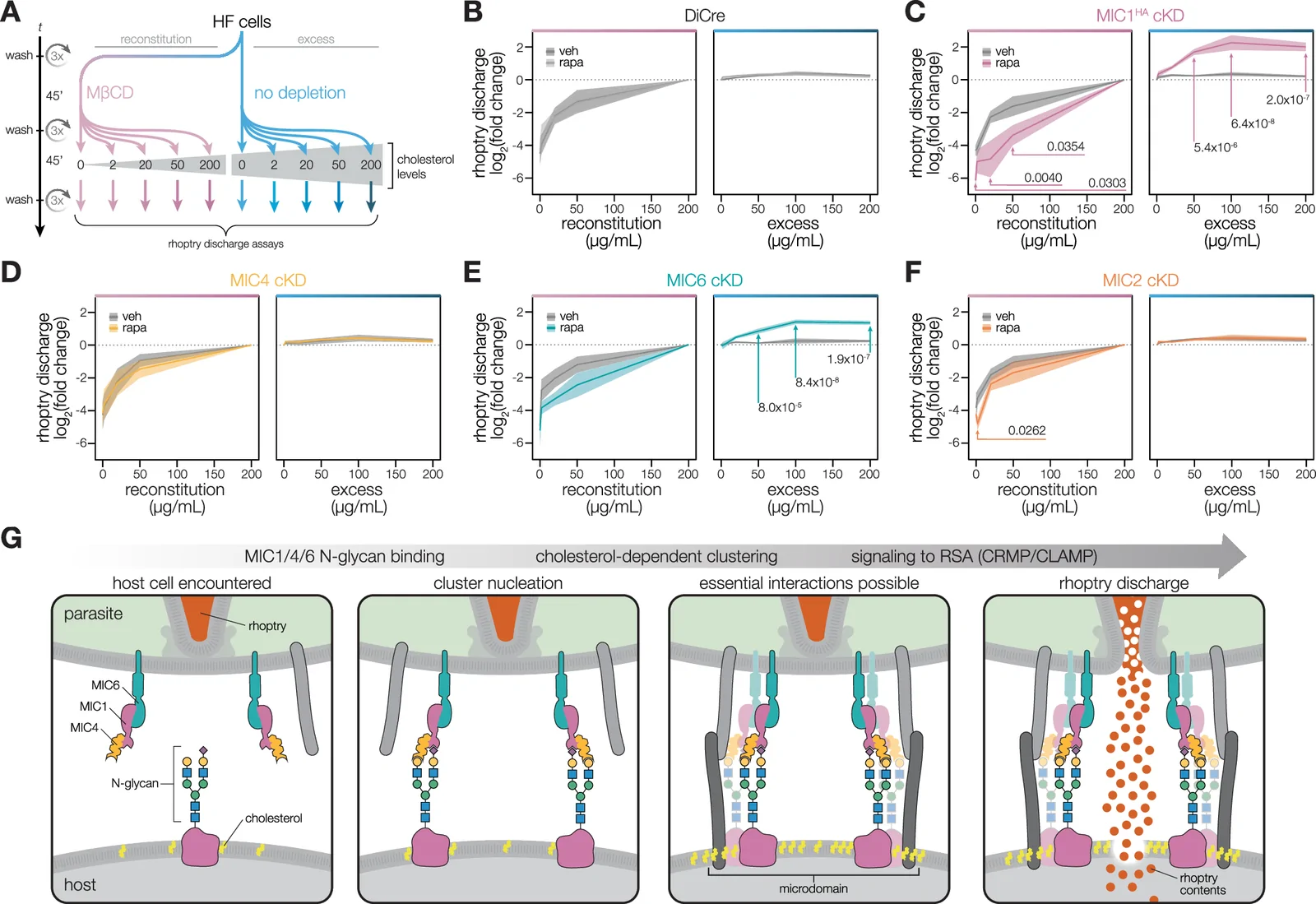
Host cholesterol levels modulate MIC1/4/6 complex activity in rhoptry discharge. (**A**) Human fibroblast cholesterol levels were either decreased to different levels through depletion and reconstitution, or increased from baseline by reconstitution without depletion. Host cells were challenged with parasites, following the various treatments. (**B**–**F**) Rhoptry discharge into cholesterol-depleted or supplemented human fibroblasts was evaluated following incubation with strains pre-treated with vehicle or rapamycin to induce recombination: wild-type, DiCre parental (**B**), MIC1^HA^ (**C**), MIC4 (**D**), MIC6 (**E**), or MIC2 (**F**). Rhoptry discharge was assessed by the relative percent of pSTAT6-positive nuclei, normalized to the highest cholesterol reconstitution level (left panels) or the vehicle-treated condition (right panels). Mean of *n* = 3 biological replicates is plotted with s.e.m. indicated by the shaded area. *P* values derived from analysis of each parasite strain separately using two-way ANOVA with Tukey’s post hoc multiple comparison test. (**G**) Model for the role of host N-glycans, cholesterol, and their coordination of rhoptry discharge into host cells by the MIC1/4/6 microneme complex. Parasite factors involved in other host cell interactions are displayed in gray. For simplicity, this diagram does not display the previously established stoichiometry of the complex (Sawmynaden et al, 2008; Marchant et al, 2012). Source data are available online for this figure.

**Figure EV6:**
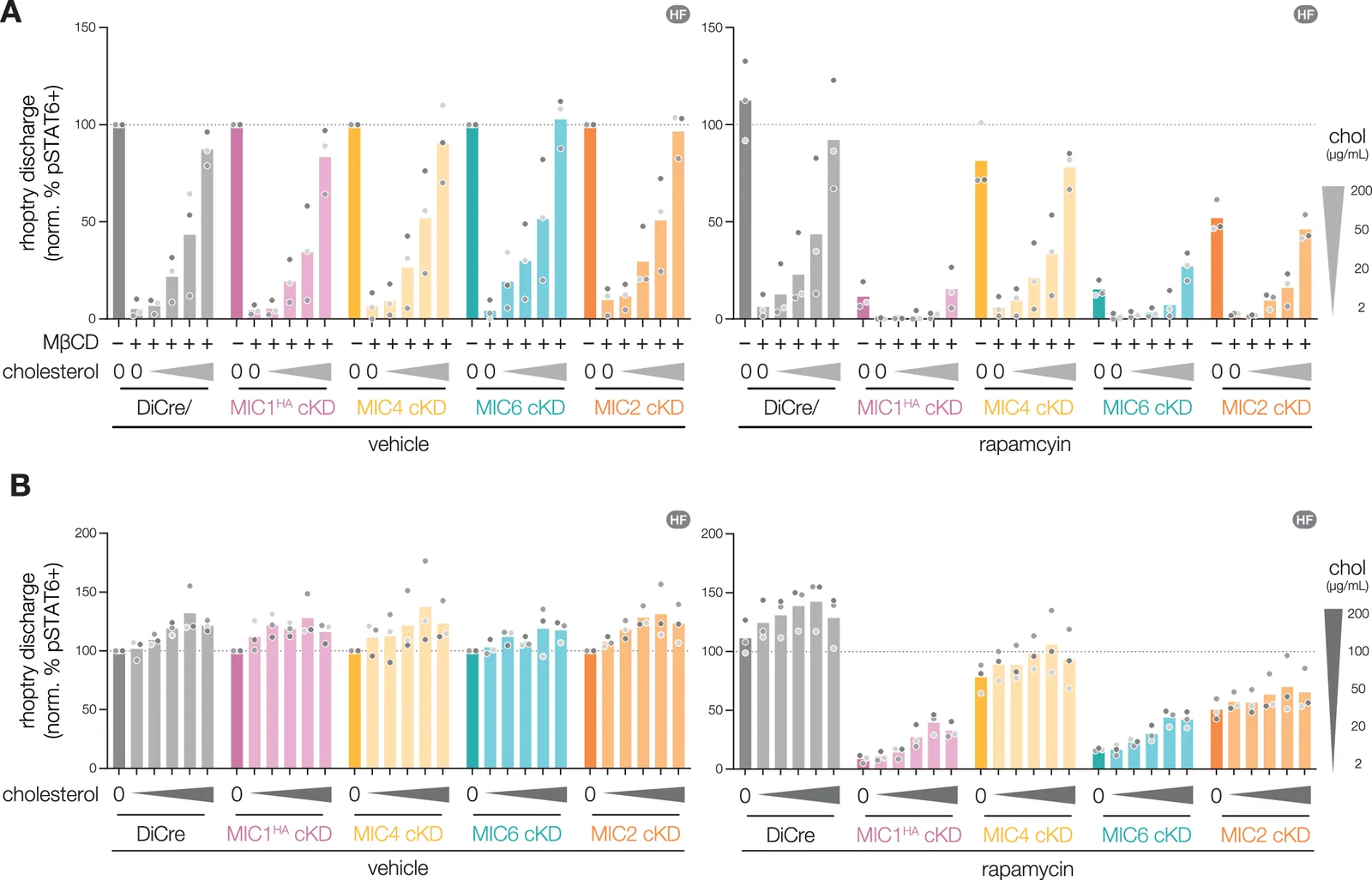
Underlying data for cholesterol level modulation interactions with the MIC1/4/6 complex. (**A**) Non-log-scaled data underlying cholesterol removal and reconstitution experiments in Fig. 6B–F. Cholesterol reconstitution levels are displayed in the legend on the right. (**B**) Non-log-scaled data underlying increased cholesterol experiments (Fig. 6B–F). Excess added cholesterol levels are displayed in the legend on the right.

We considered the relationship between the recognition of host N-glycosylation by the MIC1/4/6 complex and host cholesterol, the second major pathway identified in our screen for rhoptry discharge factors (Fig. 1C). By analogy, clathrin-independent carrier (CLIC) endocytosis (also known as GEEC) relies on galectin-mediated clustering of N-glycosylated proteins into microdomains, which depends on the presence of cholesterol in the plasma membrane (Lakshminarayan et al, 2014; Johannes et al, 2015). We hypothesized that the MIC1/4/6 complex acts analogously to galectins, clustering N-glycosylated proteins in a cholesterol-dependent manner to mediate efficient rhoptry discharge. To test this hypothesis, we measured rhoptry discharge in the various MIC knockdowns under varying levels of host plasma membrane cholesterol. We depleted host cell membrane cholesterol, as we had previously done, by MβCD extraction followed by varying levels of reconstitution. We also increased host cell membrane cholesterol by loading host cells with excess cholesterol (Fig. 6A). Following membrane cholesterol modulation, host cells were challenged with parasites that had been treated with either vehicle or rapamycin to induce knockdown of the various MIC proteins (Figs. 6B–F and EV6A,B). As anticipated from our earlier experiments, lowering host cholesterol decreased rhoptry discharge by the wild-type DiCre strain, while increasing host cholesterol had a negligible effect (Fig. 6B). These trends were unaffected in the parental strain by rapamycin treatment. Similar trends were observed when knocking down MIC2 or MIC4 (Figs. 6D,F and EV6A,B). By contrast, knockdown of MIC1 or MIC6 sensitized parasites to lowered cholesterol levels, whereas increasing host cholesterol significantly enhanced rhoptry discharge by these strains (Figs. 6C,E and EV6A,B). Together, these results suggest the MIC1/4/6 complex is orchestrating clustering of N-glycosylated proteins and cholesterol to mediate host recognition or pore formation for rhoptry discharge (Fig. 6G).

## Discussion

As apicomplexan proteins required for rhoptry discharge have been discovered, the role of the host cell in this process has remained elusive. We performed an unbiased host-directed screen focused on the essential step of rhoptry discharge. Although we cannot fully exclude effects on attachment, our screen uncovered that host galactose-containing N-glycans and cholesterol are required for *T. gondii* rhoptry discharge. Analyzing dual perturbations in the parasite and host, we conclude that these pathways act in parallel to known rhoptry discharge factors, including the CLAMP and CRMP complexes (Sparvoli et al, 2022; Singer et al, 2023; Valleau et al, 2023; Gras et al, 2017). Instead, we find that the previously characterized MIC1/4/6 complex plays a novel role in binding host N-glycans capped with galactose or sialic acid to mediate rhoptry discharge. Based on the observed cholesterol dependency of these interactions, we propose that the MIC1/4/6 complex nucleates the formation of microdomains rich in N-glycans and cholesterol. The induced microdomains are likely required for downstream steps in rhoptry discharge, potentially promoting receptor binding by the CLAMP and CRMP complexes or establishing the mechanical requirements for host cell permeabilization. Our work represents a novel framework through which to understand conserved and divergent aspects of apicomplexan rhoptry discharge.

Rather than a specific protein providing the crucial receptor, our screen found that rhoptry discharge depends on N-glycosylation. We expect there is redundancy in which N-glycosylated host proteins are important for rhoptry discharge, so alternative experimental methods will be needed to identify them. Two of the strongest effects observed in our screen were caused by the disruption of *UGP2* and *SLC35A2*, which are required for the biosynthesis and transport of UGP-galactose for galactosylation of lipids and proteins. Galactosylation was crucial for rhoptry discharge as knockout of *SLC35A2* or *UGP2*, or the exogenous addition of galactose-containing sugars, severely decreased rhoptry discharge. The role of galactosylation could be specifically localized to complex-type N-glycans based on the specific effect of kif treatment. Complex N-glycans are typically capped with either galactose or sialic acid, yet knocking out the sialic acid transporter SLC35A1 only modestly affected rhoptry discharge. The stronger effect of SLC35A2 knockout can be attributed to galactosylation being required for sialic acid to be added to N-glycans. As the MIC1/4/6 complex can redundantly bind both galactose and sialic acid, both interactions must be inhibited to completely disrupt the function of the complex. This dual inhibition is achieved by either genetic removal of galactosylation or by combined disruption of sialylation (SLC35A1 KO) with exogenous galactose treatment. Knockdown of the galactose-binding MIC4 also breaks this redundancy, rendering rhoptry discharge highly dependent on sialylation. Redundant binding of galactose and sialic acid by the MIC1/4/6 complex might contribute to the broad tissue tropism of *T. gondii*, accounting for differences in glycosylation throughout host cell types. Consistent with the hierarchy of events during apicomplexan invasion, all treatments that affect rhoptry discharge also inhibit invasion. Crucially, exogenous galactose and genetic disruption of MIC1 did not affect attachment of parasites to host cells, placing the recognition of host galactose-containing N-glycans by the parasite MIC1/4/6 complex after attachment, most likely at the step of rhoptry discharge.

Our experiments corroborated previous work implicating the MIC1/4/6 complex in glycan binding (Marchant et al, 2012; Lourenço et al, 2001; Friedrich et al, 2010; Sardinha-Silva et al, 2019). MIC1, MIC4, and MIC6 were the most highly enriched microneme proteins on lactose- and galactose-coated beads. Previous studies characterized the sugar-binding profile and assembly of the MIC1/4/6 complex (Marchant et al, 2012; Lourenço et al, 2001; Friedrich et al, 2010; Sardinha-Silva et al, 2019). The MIC1 MAR domain has a clear preference for α2,3-linked sialic acids (Friedrich et al, 2010), while the fifth of the six MIC4 Apple domains binds both β1,3- and β1,4-linked galactose (Marchant et al, 2012). MIC6 lacks lectin domains, but anchors the rest of the complex via its transmembrane helix. The two EGF domains of MIC6 bind MIC1’s galectin-like domain, and MIC1, in turn, tethers MIC4 by binding its first two Apple domains (Reiss et al, 2001; Sawmynaden et al, 2008). We conclude that binding of the complex to galactose and lactose beads occurs through MIC4, with the proposed higher-order assemblies of the complex likely contributing to its affinity (Marchant et al, 2012; Sawmynaden et al, 2008).

Cell surface glycans are hot spots for host-pathogen interactions (Gagneux et al, 2022), and different glycans can dramatically influence susceptibility and tropism. For influenza, the abundance of α2,6 and α2,3-linked sialic acids in airway epithelial cells represents a key determinant of host species tropism, impacting the differential susceptibility of humans and birds to different viruses (Viswanathan et al, 2010; Air, 2014). The glycans bound by *T. gondii* are broadly distributed across mammalian cell types. MIC1 and MIC4 bind N-glycans with α2,3-linked sialic acid and terminal galactose, respectively. Such glycans are found on cells in every tissue, though their relative abundances vary between cell types and species (Ashwood and Cummings, 2026; Otaki et al, 2022; Sardinha-Silva et al, 2019; Marchant et al, 2012; Friedrich et al, 2010), which may explain differences between CHO cells and human fibroblasts in our work. For example, MIC4 disruption has a negligible impact on rhoptry discharge into wild-type CHO cells but reduces rhoptry discharge into human fibroblasts by ~50%. Glycan density may also vary, which may explain the more pronounced effect of exogenous glycans on rhoptry discharge into human fibroblasts. Alternatively, the lack of α2,6-linked sialic acids in CHO cells (Baba et al, 2015), may increase display of α2,3-linked sialic acids that are preferentially bound by MIC1. Analogously, mammalian Galectin-3 binds α2,3-linked sialylated N-glycans to form microdomains—as we have proposed for MIC1/4/6—and its binding is reduced by α2,6-linked sialylation (Hirabayashi et al, 2002; Zhuo and Bellis, 2011; Zhuo et al, 2008). Differences in the ratio of complex- and hybrid-type N-glycans to mannose-type N-glycans may also contribute to cell-type-specific differences in rhoptry discharge and invasion. Interestingly, the malarial parasite *P. falciparum* uses the *Pf*RCR complex to bind α2,6-linked sialic acid N-glycans, which might dictate its narrower tropism compared to *T. gondii*’s (Sauer et al, 2019; Day et al, 2024).

Additional parasite lectins likely exist, since we have not profiled sialic acid-binding or all possible galactose linkages and conformations. However, our genetic interaction studies indicate that MIC1 and MIC4 redundantly control the major interactions with host N-glycans during rhoptry discharge. The general binding of N-glycans by MIC1 and MIC4 may play additional roles during infection, which would act in parallel to the functions in rhoptry discharge presented here. In certain cell types, N-glycan binding by MIC1 and MIC4 activates innate immunity signaling via Toll-like receptors (TLRs) (Sardinha-Silva et al, 2019). While rhoptry discharge may have broader impacts on innate immunity (Sloves et al, 2015), activation can be induced by recombinant MIC1 and MIC4 in the absence of *T. gondii* infection, and loss of MIC1 reduced cytokine production and virulence in mice infected with *T. gondii*. It is unclear whether this response is a specific adaptation or a consequence of N-glycan clustering on immune cells. Indeed, clustering can be a general mechanism for receptor activation (Xie et al, 2025).

Our screen identified cholesterol biosynthesis as a second major host factor involved in rhoptry discharge. Cholesterol is an essential plasma membrane lipid that regulates membrane fluidity, remodeling, and clustering of membrane components (Subczynski et al, 2017). Cholesterol is also required for *T. gondii* invasion (Tahara et al, 2016; Coppens and Joiner, 2003), appearing to be enriched at the invasion site (Besteiro et al, 2008; Coppens and Joiner, 2003). Earlier work also implicated host cholesterol in rhoptry discharge by following the translocation of certain markers by microscopy (Coppens and Joiner, 2003; Tahara et al, 2016). Removal of cholesterol from erythrocytes analogously blocks *P. falciparum* invasion (Ahiya et al, 2022; Maier and van Ooij, 2022). In our study, we modulated host cholesterol levels using MβCD, a cyclodextrin commonly used to extract cholesterol (Zidovetzki and Levitan, 2007; Coppens and Joiner, 2003; Tahara et al, 2016). We controlled for the detrimental effects of cholesterol extraction by using short treatments and, crucially, showing that reconstitution of cholesterol rescued rhoptry discharge and invasion in a dose-dependent manner. Depletion of host cholesterol sensitizes parasites to loss of the MIC1/4/6 complex, whereas excess cholesterol compensates for the loss of MIC1 and MIC6, suggesting that N-glycan binding and cholesterol orchestrate related events.

Cholesterol may play several roles in rhoptry discharge. We propose that MIC1/4/6 participates in microdomain nucleation, analogously to galectin-3 clustering of glycoproteins into cholesterol-rich microdomains that induce membrane bending and clathrin-independent endocytosis (CLIC/GEEC) in mammalian cells (Ayyar et al, 2023; Lakshminarayan et al, 2014). Galectins in CLIC/GEEC can also be inhibited by exogenous lactose or cholesterol depletion (Yang et al, 2023; Candor et al, 2025; Lakshminarayan et al, 2014). Alternatively, concentrated host cholesterol may signal host contact or facilitate the mobility or stability within microdomains of proteins required for rhoptry discharge. Proteins enriched in cholesterol-dependent lipid rafts are generally incorporated into the parasitophorous vacuole, along with cholesterol and the sphingolipid GM1 (Sawmynaden et al, 2008; Coppens and Joiner, 2003; Charron and Sibley, 2004; Shen and Sibley, 2012). Cholesterol also alters the mechanical properties of membranes, which may be required for membrane deformation or translocation of rhoptry proteins. Rhoptry discharge into the extracellular space has not been observed; however, this may be attributed to the sensitivity of current methods. Rhoptry-driven host cell perforation may behave similarly to cytolysin toxins, which use glycan recognition in combination with cholesterol to form pores in the target plasma membrane (Shewell et al, 2020). More precise biophysical methods will be needed to understand cholesterol’s role in rhoptry discharge.

Also unclear is the interplay between clade-specific factors like MIC8 and the MIC1/4/6 complex, and the pan-apicomplexan CRMP and CLAMP complexes. *P. falciparum* depends on clade-specific N-glycan-binding proteins, such as the aforementioned *Pf*RCR, to invade erythrocytes (Day et al, 2024), but additionally utilizes pan-apicomplexan complexes for this process (Valleau et al, 2023; Singer et al, 2023; Sparvoli et al, 2022). Under the proposed model, the microdomains formed by the clade-specific parasite factors may be required for the function of pan-apicomplexan factors or may simply act as parallel requirements for rhoptry discharge. This model is consistent with the additive effects we have documented between perturbations of host N-glycosylation or cholesterol and genetic disruptions affecting the CLAMP or CRMP complexes.

In summary, the MIC1/4/6 microneme complex coordinates host N-glycans and cholesterol in rhoptry discharge and invasion. We propose that these host–parasite interactions form microdomains rich in lipids and proteins, which support efficient host cell interactions by other microneme complexes or provide a mechanical scaffold that facilitates rhoptry discharge and parasite invasion. Future work will be required to understand the functions of these microdomains. The role of these interactions in parasite invasion suggests they could be therapeutic targets. The MIC1/4/6 complex has been tested as a potential vaccine target, and immunization with purified MIC1 and MIC4 produced a protective response against *T. gondii* infection (Lourenço et al, 2006). Additionally, a strain lacking MIC1 and MIC3 was effective as a live-attenuated vaccine in mice (Ismael et al, 2006). Targeting parasite-host glycan interactions with small-molecule inhibitors has recently shown promise in experiments with *P. falciparum* (Day et al, 2024). Beyond its therapeutic potential, this work refines our mechanistic understanding of rhoptry discharge, providing a conceptual framework to evaluate the hierarchy of the other host-parasite interactions required for invasion.

## Methods

**Table:** Reagents and tools table

Reagent/resource	Reference or source	Identifier or catalog number
**Experimental models**		
HFF: human foreskinfibroblast	ATCC	SCRC-1041
K562	Cellosaurus	CVCL_0004
K562 SLC35A2 KO	This study	
K562 UGP2 KO	This study	
CHO Pro-5	Gift from Richard Cummings and Pamela Stanley (Patnaik and Stanley, 2006; Esko et al, 2022)	CVCL_4382
CHO Lec2 [SLC35A1 KO]	Gift from Monty Krieger (Patnaik and Stanley, 2006; Esko et al, 2022)	
CHO Lec8 [SLC35A2 KO]	Gift from Richard Cummings and Pamela Stanley (Patnaik and Stanley, 2006; Esko et al, 2022)	
*Toxoplasma gondii* strain: RHΔ*ku80*Δ*hxgprt* (“wild-type”)	Gift from Vern Carruthers (Huynh et al, 2014)	
*Toxoplasma gondii* strain: RHΔ*ku80*Δ*hxgprt*/Δ*spatr* (“Δ*spatr”)*	Gift from Vern Carruthers (Huynh et al, 2014)	
*Toxoplasma gondii* strain: *RH*Δ*ku80*Δ*hxgprt*/dTomato (alias: TgDV101)	This study	
*Toxoplasma gondii* strain: RHΔ*ku80*Δ*hxgprt*/Δ*spatr*/dTomato (alias: TgDV104, “Δ*spatr”)*	This study	
*Toxoplasma gondii* strain: DiCre/MyoH-U1/Toxofilin-βla	This study	
*Toxoplasma gondii* strain: RH*Δku80*Δ*hxgprt*/DiCre	Hunt et al, 2019	
*Toxoplasma gondii* strain: RHΔ*ku80*Δ*hxgprt*/DiCre/Ty-APH (alias: “DiCre”)	This study	
*Toxoplasma gondii* strain: RHΔ*ku80*Δ*hxgprt*/DiCre/Ty-APH/MIC1^HA^ cKD/ mNeonGreen (alias: “MIC1^HA^ cKD”, TgDV112)	This study	
*Toxoplasma gondii* strain: RHΔ*ku80*Δ*hxgprt*/DiCre/Ty-APH/MIC2 cKD/mNeonGreen (alias: “MIC2 cKD”, TgDV121)	This study	
*Toxoplasma gondii* strain: RHΔ*ku80*Δ*hxgprt*/DiCre/Ty-APH/MIC4 cKD/mNeonGreen (alias: “MIC4 cKD”, TgDV118)	This study	
*Toxoplasma gondii* strain: RHΔ*ku80*Δ*hxgprt*/DiCre/Ty-APH/MIC6 cKD/DHFR (alias: “MIC6 cKD”, TgJL05)	This study	
*Toxoplasma gondii* strain: RHΔ*ku80*Δ*hxgprt*/Tir1/CRMPa-mNeonGreen-mAID (alias: “CRMPa cKD”, TgAL003)	This study (parental Tir1 strain: Brown et al, 2017, Long et al, 2017)	
**Recombinant DNA**		
pX458	Addgene	cat #48138
pDV046_UGP2_3_sgRNA_pX458	This study	
pDV045_SLC35A2_3_sgRNA_pX458	This study	
pDV102_HiT_MIC1_HA-U1_mNG	This study	
pDV104_HiT_MIC4-U1_mNG	This study	
pDV105_HiT_MIC2-U1_mNG	This study	
pAL003_ HiT_CRMPa_V5-TEV-mNeonGreen-mAID-Ty	This study	
pTUB-Toxofilin-β-lactamase-HXGPRT	Koshy et al, 2010, Koshy et al, 2012	
pEC325_gRNA_APH_nTerm	This study	
pJL037_Cas9_218520_MIC6	This study	
**Antibodies**		
Anti-SAG1 (DG-52)	Gift from L. David Sibley (Burg et al, 1988)	
Anti-MIC2	Gift from L. David Sibley (Achbarou et al, 1991)	
Anti-CDPK1	Waldman et al, 2020	
Anti-pSTAT6 (reactive to human cells)	Abcam	Cat #: ab188080
Anti-pSTAT6 (reactive to CHO cells)	Cell Signaling	Cat #: 56554, RRID: AB_2799514
Anti-HA (16B12)	BioLegend	Cat #: 901501, RRID: AB_2565006
Anti-mouse Alexa Fluor 488	Thermo	Cat #: A-11029, RRID: AB_2534088
Anti-mouse Alexa Fluor 594	Thermo	Cat #: A-11005, RRID: AB_141372
Anti-rabbit Alexa Fluor 488	Thermo	Cat #: A-11034, RRID: AB_2576217
Anti-guinea pig Alexa Fluor 488	Thermo	Cat #: A-11073, RRID: AB_2534117
Anti-guinea pig Alexa Fluor 594	Thermo	Cat #: A-11076, RRID: AB_141930
Anti-guinea pig Alexa Fluor 647	Thermo	Cat #: A-21450, RRID: AB_2535867
**Oligonucleotides sequences**		
oDV136_UGP2_3_sgRNA	This study	gaaaggacgaaacaccgAAAGGACGTGTCTTACTCAGgttttagagctagaaatagc
oDV135_SLC35A2_3_sgRNA	This study	gaaaggacgaaacaccgGAGCACGGAGAACAGCGCTGgttttagagctagaaatagc
oEB080_5-ToxofilinBla_F	This study	tgcattcgctccggttttgcacagccgttctgtctcacgagtgtcactgtagcctgccag
oEB081_ 3-ToxofilinBla_R	This study	tgtcggtgggtgccggctcgtctgtcgcaggtggcctgcaCGTCGACTGGAACtaCGGTG
oALH091_DHFR_3’utr_R_NL1	This study	tgtcggtgggtgccggctcgtctgtcgcaggtggcctgcagtgtcactgtagcctgccag
oALH092_TUB1_5’utr_F_NL1	This study	tgcattcgctccggttttgcacagccgttctgtctcacgaCATGCATGTCCCGCGTTCG
oJL095_MIC6_insert_F1	This study	CTCGTTGATTTGGATAGCAAAACATGGGATTAACTGCAGGATAACAGATAACTTCG
oJL096_MIC6_insert_F2	This study	AGTCGAAGTCCTCGTTGATTTGGATAGCAAAACAT
oJL097_MIC6_insert_R	This study	GTCACCTCTCGCATTGCAGCTGATCTGCCAGCGACACCCTTCCTGCAAGTGCATAGAAGG
oEB91_5-APH-N21-Ty	This study	TTTGACAGGTGGGGGAAGCGGGCTCCAGATGGACCaCTTCTGGGATCCGAGGTCCACACG
oEB92_3-APH-N21-Ty	This study	AATAATATCGCTTCCTCCTCCAGCGGTTTTCGCTTTGATGTGATCGAGCGGGTCCTGGTT
oDV073_CDPK3_3’utr_R	This study	GTCGGTCATGTTACGTGGCG
oDV477_U1_Hit_Excision_check_R	This study	acggccagtgaattggagatcgg
oDV0385_MIC1_integration_check_endo_F	This study	CACTAAACTGGATGTTGCTGTAGGCTCG
oDV482_MIC1_3utr_endo_R	This study	GATTCACTACTCAAGAAACATCTGACCGCG
oDV387_MIC2_integrationcheck_endo_F	This study	TGATACTGCTTGGCGCAGCTGG
oDV478_MIC2_3utr_endo_R	This study	GTGTAAACACCGAATAAAGCCGCGC
oDV386_MIC4_integrationcheck_endo_F	This study	CTCAACGCGGAAGCAACTATGGC
oDV479_MIC4_3utr_endo_R	This study	CGAAGCATACATACACGAAGGTGGC
oDV480_MIC6_cds_endo_F	This study	CCATATCACCTGCAAGCGTGTGCC
oDV481_MIC6_3utr_endo_R	This study	acagaagccggcctcgacacag
**Chemicals, enzymes, and other reagents**		
Mycophenolic acid (MPA)	Sigma	M3536
Xanthine (XA)	Sigma	X4002
Pyrimethamine	Sigma	46706
Rapamycin	Sigma	R0395
Hoechst 33258	Santa Cruz	sc-394039
Hoechst 33342	Invitrogen	H3570
indole-3-acetic acid (IAA)	Sigma	I2886
Pluronic F-68	Thermo	24040-032
DRAQ7	BioLegend	424001
FluoroBrite DMEM	Gibco	A1896701
DMEM	Gibco	11965118
Ham’s F-12 Nutrient Mix	Gibco	11765047
RPMI-1640	Gibco	11875085
10X Hanks’ Balanced Salt Solution (HBSS)	Gibco	14185052
Heat-inactivated fetal serum	Sigma	F4135
Newborn calf serum	Sigma	N4762
Gentamicin 1000X	Gibco	15710072
Penicillin–Streptomycin 100X	Gibco	15140122
L-glutamine	Gibco	A291680
LiveBLAzer™ FRET-B/G Loading Kit with CCF4-AM	Thermo	K1095
GS-II lectin from *Griffonia simplicifolia* conjugated to Alexa Fluor 647	Invitrogen	L32451
Wheat Germ Agglutinin (WGA) conjugated to Alexa Fluor 555	Invitrogen	W32464
Concanavalin A (ConA) conjugated to Alexa Fluor 488	Invitrogen	C11252
Cholera toxin subunit B (CTB) conjugated to Alexa Fluor 647	Invitrogen	C34778
Mycalolide B	Fujifilm Wako Pure Chemical Corporation	132-1208
Mycalolide B	Enzo	BML-T123-0020
Heparin sodium salt from porcine intestinal mucosa	Sigma	H4784
D-galactose (D-gal)	Sigma	G5388
L-galactose (L-gal)	AFG Bioscience	C53477
Mannose (Man)	Sigma	M6020
N-acetyl-galactosamine (GalNAc)	Sigma	A2795
N-acetyl-glucosamine (GlcNAc)	Sigma	A3286
N-Acetylneuraminic acid (Neu5Ac, Sia)	Sigma	A2388
Methyl-β-cyclodextrin (MβCD)	Sigma	C4555
Water-soluble cholesterol	Sigma	C4951
Benzyl-α-GalNAc	Cayman Chemical Company	31747-100
kifunensine	Sigma	K1140
eliglustat	Selleck Chemicals	S7852
AZDye 488 NHS Ester	Vector Laboratories	FP-1013-1
AzDye 647 NHS Ester	Vector Laboratories	FP-1121-1
CellTracker Red CMTPX	Invitrogen	C34552
Cal-520 AM	AAT Bioquest	20651
Probenecid, water-soluble	Invitrogen	P36400
Halt protease inhibitor cocktail 100X	Thermo Scientific	87786
Benzonase	Millipore	E1014-25KU
D-mannose agarose	Sigma	M6400
D-galactose agarose	Pierce	PI20372
α-Lactose agarose	Sigma	L7634-1ML
Pierce™ Micro-Spin Columns	Pierce	89879
TCEP	Pierce	PI20490
Chloroacetamide	Thermo	148415000
Trypsin/LysC mix	Thermo	A40007
Hydrophilic Sera-Mag SpeedBead carboxylate-modified [E7] magnetic particles	Cytiva/GE Healthcare	45152105050250
Hydrophobic Sera-Mag SpeedBead carboxylate-modified [E3] magnetic particles	Cytiva/GE Healthcare	65152105050250
**Software**		
FIJI/ImageJ version 1.54 f	NIH (https://imagej.net/ij/)	
GraphPad Prism version 10 & 11	GraphPad Software (http://www.graphpad.com)	
FlowJo version 10.10	BD Biosciences (https://www.flowjo.com)	
R version 4.4.2 & 4.2.1	CRAN (https://cran.r-project.org/)	
MAGeCKFlute	Wang et al, 2019	
Proteome Discoverer version 2.4	Thermo	
Image Lab version 6.1	Bio-Rad	
NIS Elements version 5.42.06	Nikon	
**Other**		
µ-Slide VI 0.4 ibiTreat imaging flow chamber	ibidi	80606
0.8 mm ID Silicone Tubing	ibidi	10841
Luer Connector Male, box of 25	ibidi	10824
Luer Lock Connector Female	ibidi	10825
Y Tube Fitting	ibidi	10827

### Mammalian cell culture and genetic manipulations

Human foreskin fibroblast (HFF-1; ATCC: SCRC-1041, referred to as HFF or HF throughout) cells were expanded in “D10”: DMEM (Gibco, 11965118) with 10% serum (heat-inactivated fetal serum [IFS]; Sigma, F4135-500ML). For maintenance of confluent flasks and *T. gondii* passaging, DMEM with 3% newborn calf serum (Sigma, N4762-500ML) was used in place of fetal serum (D3C). All complete DMEM medium was supplemented with 2 mM L-glutamine (Gibco, A291680) and 10 μg/mL Gentamicin (Gibco, 15710072). K562 cells (Cellosaurus: CVCL_0004) were grown in “R10”: RPMI-1640 medium (Gibco, 11875085) supplemented with 10% IFS (Sigma, F4135-500ML), 2 mM L-glutamine, and 10 μg/mL Gentamicin. CHO cells— Pro-5 (parental; Cellosaurus: CVCL_4382), Lec2 (SLC35A1 KO), Lec8 (SLC35A2 KO)—were all grown in Ham’s F-12 Nutrient Mix (Gibco, 11765047), supplemented with 10% IFS, 2 mM glutamine, and 1:100 Penicillin–Streptomycin (Gibco, 15140-122). All cells were maintained at 37 °C and 5% CO_2_.

To generate UGP2 KO and SLC35A2 KO K562 cells, guides targeting the coding region were cloned into the pX458 vector expressing *Sp*Cas9-T2A-eGFP (Addgene Plasmid #48138) to generate plasmids pDV046_UGP2_3_sgRNA_pX458 and pDV045_SLC35A2_3_sgRNA_pX458. 20 µg of these plasmids was heat-sterilized (65 °C, 15 min) and combined with 4 × 10^6^ K562 cells in 500 μL of serum-free RPMI-1640 and transferred to a 4 mm electroporation cuvette (BTX, 45-0126) on ice. An ECM-830 square wave electroporator (Harvard Instruments, BTX) was used to electroporate the cells (Low Voltage Mode, 250 V, 9 msec pulse length, field strength 625 V/cm). Transfected cells were immediately transferred to complete R10 medium. After 24 h recovery, GFP-expressing cells were sorted into 96-well plates and grown for 12–14 days, then growing clones were transferred to 12-well plates. The lectin GS-II from *Griffonia simplicifolia* conjugated to Alexa Fluor 647 (Invitrogen, L32451), which binds terminal GlcNAc, was used to positively screen clones for successful knockouts of UGP2 and SLC35A2 (Wang et al, 2020; Bojar et al, 2022). Cells were washed once with FACS buffer (PBS with 1% IFS, 1 mM CaCl_2_, 1 mM MgCl_2_, and 1 mM MnCl_2_), stained with 5 μg/mL GS-II in FACS buffer for 15–30 min on ice, then resuspended in lectin-free FACS buffer and analyzed using flow cytometry using a MACSQuant VYB flow cytometer (Miltenyi Biotec).

K562 cells with UGP2 KO (clone 3 C.2) and SLC35A2 KO (clone 2B.1) were grown the same way as parental K562 cells. For galactose recovery experiments, K562 cells were grown in R10 supplemented with 25 mM galactose for 3 days (or a water vehicle control). To assess the change in surface glycosylation patterns in the presence of galactose, GS-II staining and flow cytometry were performed as above at the same time point as the rhoptry discharge assays.

### *T. gondii* genetic manipulation and validation

To generate the DiCre/MyoH-U1/Toxofilin-βla strain, a HXGPRT-Toxofilin-βla repair template was amplified from a previously described vector used to generate transgenic Toxofilin-βla strains (Lodoen et al, 2010) using primers oEB080 and oEB081 containing homology arms for a defined neutral genomic locus (Markus et al, 2019). The repair template was co-transfected with a gRNA/Cas9 containing plasmid targeting the neutral locus for double-stranded break (Markus et al, 2020), then selected for integration with 200 μg mycophenolic acid (MPA) and 50 μg xanthine (XA). After selection, clonal parasites were isolated by limiting dilution.

To generate RH/Δ*ku80*/Δ*hxgprt*/dTomato (TgDV101), primers oALH091 and oALH092 were used to amplify a dTomato with a pTub1 promoter and a DHFR 3′ UTR (from pBM017) with homology arms for the neutral locus. Parasites were transfected with a gRNA/Cas9 containing plasmid targeting the neutral locus, and the next day, single parasites were sorted by FACS into 96-well plates. The dTomato SPATR knockout strain—RH/Δ*ku80*/Δ*hxgprt*/Δ*spatr/*dTomato—was generated in the same way as a previously constructed RH/Δ*ku80*/Δ*hxgprt*/Δ*spatr* strain (Huynh et al, 2014).

The strain background for U1 conditional knockdowns is RH/Δ*ku80*/Δ*hxgprt*/DiCre/Ty-APH, and is referred to as “DiCre” throughout. This strain was generated in the previously characterized stable DiCre background (Hunt et al, 2019) by co-transfecting a PCR-amplified template containing the Ty tag sequence with homology arms to the N-terminus of the microneme surface protein APH alongside a gRNA/Cas9 plasmid with a gRNA targeting the N-terminus of the *APH* coding sequence. A clonal population was isolated via limiting dilution without prior selection. To generate conditional knockdown strains using the U1 system, we used HiT vectors (Smith et al, 2022) that target a construct with the 3’ UTR of CDPK3 between loxP sites, followed by 4xU1 motifs to the C-terminal codon of the target gene (Pieperhoff et al, 2015). In this system, rapamycin addition leads to excision of the CDPK3 3’ UTR and appends the U1 motifs, such that the mRNA is targeted for mislocalization and degradation, leading to protein knockdown. The HiT vectors used for making MIC1^HA^ cKD, MIC4 cKD, and MIC2 cKD strains contained a mNeonGreen fluorescent protein cassette as a selectable marker and were isolated by single parasite FACS sorting. The MIC6 cKD strain was made instead by PCR-amplification of a homologous recombination template containing the same U1 regulatory elements, but instead using a DHFR selectable marker, so pyrimethamine selection and isolation by limiting dilution were used to isolate clonal parasites. For all strains, correct integration of constructs into parasites was validated by PCR (Fig. EV5A,B). Two days after parasites were treated with 50 nM rapamycin for 2 h, correct DiCre-mediated excision of the CDPK3 3’ UTR and appending of the tandem U1 array was also validated by PCR (Fig. EV5A,C). Where possible—for MIC1^HA^ cKD and MIC2 cKD—knockdown was additionally validated by immunofluorescence assays (IFA). For IFAs, parasites were treated with 50 nM rapamycin when added to HFF cells, followed by rapamycin washout 2 h later. Two days later, infected HFF cells were fixed and permeabilized with 100% −20 °C methanol for 8 min, and blocked with blocking buffer (5% IFS and 1% normal goat serum in PBS). Samples were immunostained with blocking buffer containing 1:1000 mouse anti-HA (BioLegend #901501), 1:1000 mouse anti-MIC2 (Achbarou et al, 1991), 1:10,000 guinea pig anti-CDPK1 (Waldman et al, 2020). After 3x PBS washes, samples were stained with anti-mouse Alexa Fluor 488 (A-11029, Thermo), anti-guinea pig Alexa Fluor 647 (A-21450, Thermo), and 1:20,000 Hoechst 33258 (sc-394039, Santa Cruz). Samples were imaged on a Nikon CSU-W1 spinning disk confocal microscope using a ×60 silicone oil objective and processed using Fiji (Schindelin et al, 2012).

To generate the CRMPa cKD strain, a HiT vector (Smith et al, 2022) targeting the C-terminus of CRMPa (TGGT1_261080) with a V5-TEV-mNeonGreen-mAID-Ty tag and the 3’ UTR of CDPK3 was generated. Alongside a Cas9 transient expression vector, this HiT vector was transfected into an RH/Tir1 strain background that heterologously expresses the F-box protein Tir1 such that indole-3-acetic acid (IAA) addition induces the degradation of mAID-tagged proteins (Smith et al, 2022; Brown et al, 2017; Long et al, 2017). The HiT vector contained a DHFR selection cassette and pyrimethamine was used to select for its integration into the CRMPa locus, then correct integration was validated using PCR. For all experiments using this RH/Tir1/CRMPa cKD (CRMPa cKD) strain, freshly lysed out parasites were added to fresh HFF monolayers and allowed to invade for 1 h. In total, 500 µM IAA or a PBS vehicle control was then spiked into the flasks to knock down CRMPa as indicated, and the parasites were grown for 2 more days.

### Host cell rhoptry discharge screen

For the host cell rhoptry discharge screen, K562 cells were maintained in shaking flasks in an Infors HT incubator at 37 °C, 125 RPM, and 8% CO_2_ with an added 0.1% Pluronic F-68 (Thermo) to prevent cell clumping and increase viability during mechanical stress from shaking.

The screen was performed with two host cell library pools, infected 3 weeks apart, with 2 replicates of the rhoptry discharge performed on each host cell pool, resulting in 4 assay replicates. Throughout library generation and selection, host cells were maintained at a targeted minimum of 1000× library coverage.

To generate the host cell library, a pooled human genome-wide optimized 5-guide library (97,888 unique guides) packaged into lentivirus prepared by the Whitehead Genome Technology Core was used to infect K562 host cells (Inglis et al, 2023). Viral transduction was performed by spinfecting 300 million K562 cells in six-well dishes with 5 million K562 cells/well at a multiplicity of infection of 0.5 (titered functional transduction units: host cell) at 1200× *g* for 45 min at 37 °C in the presence of 8 μg/mL polybrene. The next day, the medium was refreshed. Two days after lentivirus infection, cells were resuspended in medium containing 2 μg/mL puromycin (Sigma) to select for guide vector incorporation. Five days after lentiviral infection, puromycin was removed, and cells were counted to ensure library coverage was maintained. On days 13 and 14, 350–450 million host cells were used to perform sorting in the rhoptry discharge assay, and 150 million untreated host cells, divided into aliquots of 50 million cells, were harvested to sequence the library diversity at the time of each assay replicate.

To generate the parasites for each replicate of the rhoptry discharge assay, 2 days prior (i.e., day 11 and 12), 70 15-cm dishes of HFF cells were each infected with 3.5 × 10^7^ DiCre/MyoH-U1/Toxofilin-βla parasites and treated with 50 nM rapamycin in D3C medium. A minimum of 2 h later, rapamycin was washed out by two washes of PBS, incubation in warmed D3C for >5 min, followed by a third PBS wash. Fresh warm D3C was added to each plate, and the plates were returned to the incubator for approximately 42 h. On the day of each rhoptry assay replicate, the plates were scraped, and parasites were released from host cells by syringe-release using 27 G needles. The parasites were filtered through 5-μm pore filters to remove debris and unlysed cells, and pelleted by centrifugation at 1000×* g* for 5 min. All tubes of parasites were pooled and washed in HBSS/HEPES (HBSS with 25 mM HEPES pH 7.4), pelleted again, and resuspended to a concentration of 400 million parasites/mL in HBSS/HEPES.

For each replicate of the rhoptry discharge assay, 350–450 million K562 cells were pelleted in a 500-mL conical tube, washed with HBSS/HEPES to remove serum, and resuspended to a final concentration of 20 million cells/mL. K562 cells were mixed 1:1 with DiCre/MyoH-U1/Toxofilin-βla parasites (400 million/mL) to yield a multiplicity of parasites:host cells of 20:1 in a 50 mL conical tube and placed in a 37 °C water bath. Approximately 8 mL of 6x solution of the CCF4-AM substrate was made up according to the kit guidelines with minor modifications (Thermo, K1095). Briefly, 14.4 μL of solution A (1 mM CCF4-AM) was mixed with 480 μL of solution B (100 mg/mL Pluronic^®^-F127 surfactant in DMSO with 0.1% acetic acid), then 7.51 mL of HBSS/HEPES was added. After 1 h of incubation, the host:parasite mixture was allowed to cool to room temperature, then the 6× CCF4-AM loading solution was added to yield a 1× CCF4-AM concentration. After a 30-min incubation at room temperature with gentle mixing, the cells were strained through a 50-mL conical cell strainer and placed on ice.

The host cells were sorted based on cleavage of the FRET CCF4-AM substrate into a rhoptry discharge positive (“blue”) and rhoptry discharge negative (“green”) population using two BD FACSAria IIIu cell sorters equipped with a 405-nm laser line and 450/40 filter. The cells were sorted using four-way purity into 15-cm conical tubes pre-coated with BSA (coated by overnight incubation with 5% BSA in PBS). We targeted sorting a minimum of 1000x library coverage, or 100 million host cells in each replicate (R1: 90 mil, R2: 140 mil, R3: 140 mil, R4: 165 mil). After sorting, aliquots of 50 million host cells were made, pelleted, and frozen for Illumina workup, quality control, and sequencing alongside the unsorted controls by the Whitehead Institute Functional Genomics Platform and Genome Technology Core on an Illumina NovaSeq.

### Screen analysis

We obtained approximately 320 million reads across 12 sequenced conditions (4 each of Unsorted, rhoptry discharge susceptible [blue], and rhoptry discharge resistant [green]). Data was analyzed using the MAGeCKFlute pipeline (Wang et al, 2019). Briefly, the pipeline was used to map sgRNAs, make count tables, minimize batch effects (using the BatchRemove script), and then the MAGeCK MLE algorithm was used to identify the importance of each knockout to rhoptry discharge. For guides that targeted multiple genes, the counts for those specific guides were duplicated amongst the co-targeted genes prior to batch correction and the MAGeCK MLE analysis. The MAGeCK MLE algorithm compared each sorted population to the unsorted controls and inferred a “beta-score”, where a positive beta-score represents enrichment in the target population. To make ranked gene plots and perform GSEA, we combined the scores from the resistant and susceptible populations into a single score, the “combined rhoptry score” (Wang et al, 2019). GSEA enrichment analysis was performed with RStudio using the fgsea “Fast gene set enrichment analysis” package (Korotkevich et al, 2016), using the Reactome database pathways (Milacic et al, 2024).

### DRAQ7 host cell viability assay

To evaluate host cell viability and membrane integrity for various chemical perturbations of human fibroblasts and CHO cell knockouts, cells were grown and treated in T12.5 cm^2^ flasks for the indicated durations. Host cells were washed with PBS, trypsinized, and collected in 2 × 1.5 mL microcentrifuge tubes per condition. Cells were pelleted by a 5-min, 300× *g* spin, then resuspended in FACS buffer containing a final concentration of 1.62 µM (1 µg/mL) Hoechst 33342 (Invitrogen, H3570) and 0.3 µM DRAQ7 (BioLegend, 424001). For the positive control for permeabilization condition, the buffer additionally contained 0.02% Triton X-100. After a 20-min incubation on ice, cells were spun down again (300×* g*, 5 min), resuspended in 0.5 mL FACS buffer, and passed through a cell strainer. DRAQ7 staining was measured using a MACSQuant VYB flow cytometer (Miltenyi Biotec), and FlowJo (BD Life Sciences) was used for analysis.

### Cell morphology analysis

To evaluate host cell morphology for various chemical perturbations of human fibroblasts and CHO cell knockouts, cells were grown and treated as indicated in microscope-grade 96-well polymer plates (Cellvis, P96-1.5 P). After treatments, cells were washed with FluoroBrite DMEM (Gibco) containing 1% IFS (F1). They were then stained with 1.62 µM (1 µg/mL) Hoechst 33342 in F1 for 10–20 min at 37 °C, then imaged using a Nikon CSU-W1 spinning disk confocal microscope using a ×40 air objective equipped with a DIC module. The images were then processed using Fiji (Schindelin et al, 2012).

### Validation of CHO cell surface glycosylation patterns

To validate the CHO Pro-5 (parental), Lec2 (SLC35A1 KO), and Lec8 (SLC35A2 KO) had the expected glycosylation patterns, CHO cells were trypsinized, washed once with FACS buffer, and stained for 15–30 min on ice with 5 μg/mL GS-II-Alexa Fluor 647 (GS-II-AF647), 1 μg/mL Wheat Germ Agglutinin-Alexa Fluor 555 (WGA-AF555; Thermo, W32464), or 50 μg/mL Concanavalin A-Alexa Fluor 488 (ConA-AF488; Thermo, C11252). After staining, cells were resuspended in lectin-free FACS buffer and analyzed using flow cytometry using a MACSQuant VYB flow cytometer (Miltenyi Biotec). FlowJo (BD Life Sciences) was used for analysis and to generate plots.

### Growth and knockdown of *T. gondii* strains

All *T. gondii* parasites were maintained by passage in HFF cells. Where noted in experiments, “WT” parasites refer to the RH/Δ*hxgprt/*Δ*ku80* background (Huynh et al, 2014). For rhoptry discharge assays with auxin-inducible knockdowns (CRMPa cKD), 2 days prior to the experiment, freshly lysed out parasites were passaged with 2–3 drops into fresh T12.5-cm^2^ flasks and allowed to invade for at least 1 h. The media was then exchanged for D10 containing 500 μM indole-3-acetic acid (IAA) or a vehicle control (PBS). Parasites were then harvested 40-44 h later. For assays using the U1-based transcriptional knockdown system, 2 days prior to the experiment, freshly lysed out parasites were passaged with 2–3 drops into fresh T12.5-cm^2^ flasks containing either 50 nM rapamycin to induce DiCre dimerization or a vehicle control (DMSO). After 2 h, the flasks were washed 3× with PBS to remove rapamycin. Fresh D10 was added, and parasites were harvested 40–44 h later.

### Toxofilin-βla rhoptry discharge assays

Toxofilin-βla secretion assays were done similarly to the screen, with minor modifications. DiCre/MyoH-U1/Toxofilin-βla parasites were prepared as in the screen: treated with rapamycin for 2 h in T12.5-cm^2^ flasks of confluent HFF cells, and harvested 40–44 h later by syringe lysis using a 27 G needle, followed by filtering through 5-μm pore filters to remove host debris. Parasites were isolated by centrifugation at 2000×*g*, resuspended in HBSS/HEPES, and counted. Parasites were then centrifuged again and resuspended to a concentration of 1 × 10^7^ parasites per 50 μL in HBSS/HEPES. To prepare host cells, K562 cells were maintained in T175 flasks such that they would be at a density of 0.5–2 × 10^6^/mL at the time of assay. Their density was assessed using a Countess II Automated Cell Counter (Thermo Fisher), then they were pelleted (300-500 g, 5 min) and resuspended to a density of 5 × 10^5^ K562 cells per 100 μL in HBSS/HEPES. In the wells of a 96-well tissue culture plate, 100 μL of K562 cells were then mixed with 50 μL of parasites (multiplicity of infection = 20), centrifuged for 5 min at 290× *g*, and incubated at 37 °C for 60 min. As in the screen, a 6× solution of CCF4-AM was prepared, but with propidium iodide added as a live/dead counterstain (Sigma, P4170; final concentration of 1 μg/mL). After letting the cells cool to room temperature, 30 μL of 6x CCF4-AM solution was added to each well. Samples were repeatedly pipetted to ensure cells were well mixed with CCF4-AM, then incubated in the dark for at least 30 min. Each condition was assessed using a MACSQuant® VYB flow cytometer (Miltenyi Biotec). As with the screen, the level of rhoptry discharge into host cells was assessed by monitoring the ratio of host cells in the blue fluorescence channel using FlowJo (BD Life Sciences). Only cells that were resistant to propidium iodide staining were used in the analysis. GraphPad Prism was used to perform statistical testing.

### STAT6 phosphorylation rhoptry discharge assays

All STAT6 phosphorylation (pSTAT6) rhoptry discharge assays were performed in black-walled 96-well plates with clear bottoms for imaging. HFF host cells were typically prepared by splitting a T12.5-cm^2^ confluent flask per 96-well plate and grown for 3 days prior to the experiment, such that they were freshly confluent at the time of the assay. CHO cells were prepared by seeding either 10,000 cells per well 3 days prior to the experiment or 25,000 cells per well 2 days prior to the experiment, such that they were >90% confluent at the time of assay.

*T. gondii* parasites grown in HFF cells in T12.5-cm^2^ flasks were typically harvested immediately prior to natural egress by scraping and passing through a 27 G syringe, followed by passing through disposable 5-μm pore size syringe filters to remove host cell debris. The parasites were pelleted (2000×*g*, 5 min), resuspended in 800 μL DMEM with 20 mM HEPES pH 7.4 added (DMEM/HEPES), transferred to 1.5-mL microcentrifuge tubes, and pelleted again. Parasites were resuspended in DMEM/HEPES with 1% IFS and 1.2 μM mycalolide B (Fujifilm Wako Pure Chemical Corporation; 132-12081 or Enzo; BML-T123-0020) in a volume of 100 μL per T12.5-cm^2^ flask of parasites prepared, then incubated at RT for 30–60 min. During this incubation, parasite density was assessed. Parasites were pelleted again and resuspended in 1 mL of D10 with 20 mM HEPES pH 7.4 (D10H) for HFF experiments or Ham’s F12 with 10% IFS and 20 mM HEPES pH 7.4 (F12H) for CHO cell experiments. Parasites were diluted to either 1 × 10^6^ parasites/mL; in the case of sugar or drug treatments during the assay, 2× working stocks 2 × 10^6^ parasites/mL were prepared. Unless otherwise noted, 2 × 10^5^ parasites/ well of a 96-well plate were used for all experiments.

For sugar treatments, 0.5 M stock solutions of L-galactose, D-galactose (Gal), lactose (Lac), mannose (Man), N-acetyl-galactosamine (GalNAc), N-acetyl-glucosamine (GlcNAc), and N-acetylneuraminic acid (Sia) were prepared in PBS. In total, 2× working solutions were prepared in the appropriate complete medium for either HFF cells (D10 + 20 mM HEPES pH 7.4; D10H) or CHO cells (Ham’s F12 + 10% IFS + 20 mM HEPES pH 7.4; F12H). The growing medium of a 96-well plate of HFF cells was replaced with 100 μL of the 2× sugar-containing medium at room temperature, followed immediately by 100 μL of 2× working stocks of indicated parasites. The plate was then centrifuged to ensure host cell contact by parasites (290 g, 5 min), and incubated at 37 °C for 2 h. Unless otherwise noted, the final 1× concentration in all sugar experiments was 10 mM.

For cholesterol depletion and add-back experiments, recently confluent HFF cells were washed 2× with PBS and 1× with DMEM lacking any additives (D0). The media was then replaced with D0 containing 20 mM HEPES pH 7.4 (D0H) and 10 mM methyl-β-cyclodextrin (MβCD), which was prepared by directly solubilizing MβCD powder (Sigma; C4555) in D0H. The cells were incubated at 37 °C for at least 45 min. For cholesterol add-back experiments, water-soluble cholesterol (Sigma; C4951) was dissolved in water at a stock concentration of 10 mg/mL, then the indicated concentration or vehicle control was added to D0H. After MβCD treatment, the HFF cells were washed as before, the indicated concentration of cholesterol was added, and the cells were incubated again at 37 °C for at least 45 min. The cholesterol treatments were washed out with 2× PBS washes and 1× D0 wash, then 200 μL parasites in complete D10H at a concentration of 1 × 10^6^ parasites/mL were added. The plate was centrifuged to facilitate parasite–host cell contact (290× *g*, 5 min), and the plate was incubated at 37 °C for 2 h.

Stocks of inhibitors were all dissolved in DMSO; 200 mM benzyl-α-GalNAc (Cayman Chemical; 31747-100), 10 mM kifunensine (Sigma, K1140), and 10 mM eliglustat (Selleck Chemicals, S7852). For benzyl-α-GalNAc and kifunensine co-treatments, HFF cells were split from 12.5-cm^2^ flasks into 96-well plates (1 flask per plate), and grown for 3 days in the presence of 2 mM BAG and 10 μM kifunensine (unless otherwise noted). For eliglustat and kifunensine co-treatments, HFF cells were split 1:9 and grown for 5 days in the presence of the indicated drug, then seeded into 96-well plates. To confirm the inhibitory effects of eliglustat on sphingolipids, BAG on O-glycans, and kif on N-glycans, after drug treatments, a variety of lectin stains was employed using the same protocol as for CHO cell validations. Concanavalin A conjugated to Alexa Fluor 488 (ConA-AF488) verified the increased abundance of exposed mannose residues following kif treatment. Wheat Germ Agglutinin conjugated to Alexa Fluor 555 (WGA-AF555), which stains GlcNAc and Neu5Ac present in O- and N-glycans, verified the inhibitory effects of BAG, kif, and the combination of the two. Cholera toxin subunit B conjugated to Alexa Fluor 647 (CTB-AF647), which binds the sphingolipid Gm1, verified the inhibitory effect of elig on sphingolipid synthesis. Rhoptry discharge assays were performed on day 7 after drug treatment. To perform rhoptry discharge assays, parasites in D10H at a concentration of 1 × 10^6^ parasites/mL were added to host cells. The plate was centrifuged to facilitate parasite–host cell contact (290× *g*, 5 min), and the plate was incubated at 37 °C for 2 h.

After 2 h at 37 °C to allow for rhoptry discharge and nuclear translocation of phosphorylated STAT6, cells were washed once with PBS, then fixed by the addition of 100% ice-cold methanol for 8 min. Wells were then washed 3× with PBS to remove methanol, then blocked for 10 min in blocking buffer (PBS with 1% goat serum [Gibco; 16210072] and 5% IFS). Samples were stained with 1:10,000 guinea pig anti-*Tg*CDPK1 (Waldman et al, 2020) and 1:1000 rabbit anti-pSTAT6 (HFF staining with Abcam ab188080, CHO staining with Cell Signaling #56554). Primary incubation was for 1 h at RT or overnight at 4 °C. Wells were washed 2× with PBS, then incubated in secondary staining solution (1:1000 anti-rabbit Alexa Fluor 488 [Life Technologies; A-11034], 1:1000 anti-guinea pig Alexa Fluor 594, and 1:20,000 Hoechst 33258 [Santa Cruz Biotechnology; SC-39403]) for 1 h at RT. Wells were washed 3× with PBS, then a final 200 μL PBS was added, and wells were imaged using a Cytation 3 or Cytation 5 Cell Imaging Multi-Mode reader (BioTek) using a ×20 objective with laser autofocus. A custom Python script was used to identify nuclei in the Hoechst channel and extract nuclear pSTAT6 signal while masking out parasites using the anti-*Tg*CDPK1 counterstain. An R analysis script and GraphPad Prism were used to threshold the percentage of pSTAT6-positive nuclei and perform statistical analysis, respectively.

### Invasion assays

All host cell treatments and parasite growth, including knockdown treatments, were performed as for rhoptry discharge assays in microscopy-grade polymer 96-well plates (Cellvis, P96-1.5 P). For all invasion assays with chemical host cell treatments (e.g., cholesterol removal/add-back, exogenous sugars) and CHO cell knockouts, the red-fluorescent wild-type strain RH/Δ*ku80*/Δ*hxgprt*/dTomato (TgDV101) was used. Briefly, following parasite harvest by syringe-release, parasites were washed once with D0, pelleted (2000×*g*, 5 min), and collected in D10, counted, and resuspended to a concentration of 1 × 10^6^ parasites/mL. 200 μL of parasites were then added to each well. In the case of exogenous sugar experiments, 2× solutions of sugar or vehicle in D10 were prepared alongside a 2× parasite stock of 2 × 10^6^ parasites/mL, and 100 μL of sugar and parasite stocks were combined in the appropriate wells. The plates were centrifuged at 300× *g* to induce host cell contact, then incubated in a 37 °C water bath. After 20 min, each well was fixed with 4% formaldehyde, washed 3× with PBS, then blocked (5% IFS and 5% goat serum in PBS). Extracellular parasites were stained with mouse anti-SAG1 DG-52 (Burg et al, 1988) primary, then with anti-mouse Alexa Fluor 488 secondary in the presence of cell-permeable Hoechst 33342.

For cKD strains (MIC1^HA^, MIC4, MIC6, and MIC2) and the parental (DiCre), the same procedure was followed, but following staining of extracellular parasites with anti-SAG1 primary, cells were permeabilized by a 5 min incubation in blocking buffer with 0.25% Triton X-100 added. Wells were washed 3× with PBS, then stained with guinea pig anti-CDPK1 (Waldman et al, 2020). Anti-mouse Alexa Fluor 594 and anti-guinea pig Alexa Fluor 488 secondaries were then added in the presence of cell-impermeable Hoechst 33258.

Following staining, all invasion assays were imaged using a Cytation 5 Cell Imaging Multi-Mode reader (BioTek) with a ×20 objective using laser autofocus. To effectively capture both intracellular and extracellular parasites, 5-layer Z-stacks were collected for at least 9 regions of each well. Max-intensity projections were analyzed for intracellular and extracellular parasites using custom Python and R scripts. Briefly, all parasites were identified by dTomato or anti-CDPK1 signal, and the subset of extracellular parasites was intensified by anti-SAG1 staining. Host cell nuclei were identified by Hoechst signal, and size thresholding removed parasite nuclei. Invasion rate was reported as the number of intracellular parasites per host cell nuclei for experiments with HFF cells and the number of intracellular parasites relative to the summed host cell nuclear area for CHO cells. GraphPad Prism was used for statistical testing and graphing.

### Kifunensine matched the invasion and rhoptry discharge assay

For time-course matched invasion and rhoptry discharge assays with kifunensine treatment, HFF cells were split 1:20 into paired microscopy-grade polymer 96-well plates (Cellvis, P96-1.5 P). Kif (10 μM) was added to one row every other day (to make treatment days 9, 7, 5, 3, and 1). On day 10 when host cells were confluent, paired plates were used for matching STAT6 rhoptry discharge or invasion assays using the same preparation of RH/Δ*ku80*/Δ*hxgprt*/dTomato (TgDV101) parasites. Plates were worked up and analyzed with standard methods. Day 0 (vehicle) treatment was used to normalize each replicate of both experiments to 100%. GraphPad Prism was used for statistical testing and graphing.

### Flow-based adhesion assays

Anti-SAG1 antibody (DG-52) was pre-conjugated using AZDye 488 NHS Ester or AzDye 647 NHS Ester (Vector Laboratories) to make anti-SAG1-488 and anti-SAG1-647. The AZDye 488 reaction was quenched using 100 mM Tris, and unconjugated fluorophore was removed using PD SpinTrap™ G-25 desalting and buffer-exchange columns (28918004, GE Healthcare/Cytiva), and the final concentration was estimated to be >0.8 mg/mL.

HFF cells were grown to confluency in 0.4 mm height IbiTreat µ-Slide VI Luer multichannel slides (ibidi). Immediately prior to the assay, HFFs were fixed with 4% formaldehyde (15 min, 4 °C), washed 3× with PBS, and FluoroBrite DMEM containing 10% IFS and 20 mM HEPES (F10H) was added. For comparison of attachment with L-galactose, D-galactose, and heparin, RH/Δ*ku80*/Δ*hxgprt*/dTomato (TgDV101) *T. gondii* parasites were used. The mNeonGreen-expressing MIC1^HA^ cKD and MIC2 cKD strains were treated with vehicle or rapamycin 2 days prior to the assay for single MIC knockdown comparisons. All parasites were syringe released and filtered with 5-μm pore filters to remove unlysed host cells and debris. Parasites were then centrifuged at 2000×*g* for 5 min, and resuspended in FluoroBrite DMEM (Gibco) containing 20 mM HEPES pH 7.4 (F0H) and a 1:200 dilution of anti-SAG1-488 for TgDV101 and a 1:400 dilution of anti-SAG1-647 for MIC1^HA^ cKD and MIC2 cKD strains. In the case of MIC1^HA^ cKD and MIC2 cKD parasites, vehicle and rapamycin conditions were split into two microcentrifuge tubes. One tube of each condition was additionally incubated with 2 μM CellTracker Red CMTPX (Invitrogen, C34552). Following a 20 min incubation, during which parasites were counted, parasites were pelleted again and resuspended in F10H to a target concentration of 4 × 10^6^ parasites/mL. In the case of MIC1^HA^ cKD and MIC2 cKD parasites, vehicle and rapamycin conditions were mixed in equal parasite proportions to make 2 technical replicates, each one with either the vehicle or rapamycin treatments dyed red with CellTracker Red CMTPX (8 × 10^6^ parasites/mL total).

The Ibidi VI slides containing fixed HFF cells were mounted in a 37 °C box-heated stage of a Nikon CSU-W1 spinning disk confocal microscope equipped with a dual-camera setup for instantaneous red/green imaging and a 40X air objective. Prepared parasites were applied to fixed HFF cells at a flow rate of 75 μL/min (~ 0.1 dyn/cm^2^) using a syringe pump and 0.8 mm ID tubing with Luer connectors (Ibidi, 10841, 10825, and 10824). Tubing passed through a 37 °C heated water bath to ensure parasites were warm upon fixed host cell contact. After 12 min of attachment, a 2-min 2000 μL/min (2.5 dyn/cm^2^) high-speed wash of parasite-free F10H medium was applied by switching to a second syringe pump connected in-line by a Y-tubing connector (Ibidi, 10827). After the wash, pumps were turned off, and 13 Z-stack images (9 slices, 0.6 μm spacing) with 0.5 mm spacing along the center of the flow path were collected. Parasites were counted using Fiji (Schindelin et al, 2012). For each mixture of vehicle and rapamycin parasites, we additionally analyzed the ratio of vehicle to rapamycin parasites by flow cytometry to account for minor deviations from the expected 1:1 ratio and applied the appropriate correction to the raw parasite counts. GraphPad Prism was then used for statistical testing and graphing.

### Host cell perforation, transient, and invasion assays

Live calcium transient/invasion assays were carried out as previously described (Male et al, 2025). Briefly, we used the protocol with Cal-520 AM as the calcium indicator (AAT Bioquest, 20651), inclusion of 1 mM probenecid (Invitrogen, P36400) throughout the protocol, and live cell imaging solution containing 8 mM CaCl2 during live imaging. For each of the three replicate experiments, CHO cell lines (WT, SLC35A1 KO [Lec2], SLC35A2 KO [Lec8]) were each seeded in two chambers of an Ibidi µ-Slide VI 0.4 (Ibidi) in Ham’s F-12 medium with glutamine (Cytiva) containing 100 units/mL penicillin, 100 µg/mL streptomycin, 10% IFS (Life Technologies), and incubated overnight at 37˚C (with 5% CO_2_ and humidity) in preparation for live experiments. As a lower level of invasion was observed in CHO cells relative to HFF cells, 2400 high-quality frames (192 s) were processed from each capture compared to the published protocol (Male et al, 2025). Due to high levels of baseline intracellular calcium activity in the CHO cells, automated identification of calcium transients was confirmed by visualization of each event as a change in fluorescence over time plot.

### Glycan bead affinity purifications

*T. gondii* RH/Δ*ku80*/Δ*hxgprt/*dTomato (TgDV101) parasites were grown in 15-cm plates of confluent HFF cells for 2 days. Parasites were harvested, filtered with 5-μm pore filters to remove host cells and debris, washed 2× with PBS via pelleting (5 min, 2000×*g*) and resuspension. Parasites were pelleted again, the final PBS wash was removed, and the pellet was flash frozen in liquid N_2_. Pellets were lysed on ice by thawing directly into cold SugarAP buffer (PBS with 1 mM CaCl_2_, MgCl_2_, 0.5% Igepal CA-630, 1 mM benzonase [Millipore; E1014-25KU], and 1× protease inhibitor cocktail [Thermo; 87786]). While parasites were lysing, 200 μL bead slurry (100 μL final bed) of D-mannose agarose (Sigma; M6400), D-galactose agarose (Pierce; PI20372), and α-Lactose agarose (Sigma, L7634) were added to spin columns (Pierce; 89879) and prepared by washing with 10 gel-bed volumes (4×250 μL) of SugarAP buffer; 40× *g* spins were used throughout for all steps. The parasite lysate was clarified by centrifugation to remove insoluble debris (4 °C, 11,000× *g*, 10 min), and the soluble lysate flowed over the resins. Resins were then washed for 20 gel-bed volumes (8×250 μL) using SugarAP buffer. Bound proteins were then specifically eluted by 100 mM of the free cognate sugar in SugarAP buffer (i.e., mannose, galactose, lactose), using 7.5 gel-bed volumes (3 × 250 μL).

Eluted proteins were prepared for mass spectrometry by SP3 bead digestion and clean-up as previously described (Hughes et al, 2019; Valleau et al, 2023). Briefly, proteins were reduced with TCEP (Pierce, PI20490) and alkylated with chloroacetamide (Thermo, 148415000), precipitated onto hydrophilic and hydrophobic SP3 beads using ethanol, washed in 80% ethanol, and digested overnight with Trypsin/LysC mix (Thermo, A40007). Eluted peptides were lyophilized and directly injected for DDA mass spectrometry on a Thermo Exploris 480 mass spectrometer and analyzed using Proteome Discoverer 2.4 (Thermo).

## Supplementary information

Peer Review File

Dataset EV1

Source data Fig. 2

Source data Fig. 3

Source data Fig. 4

Source data Fig. 5

Source data Fig. 6

Dataset EV2

Expanded View Figures

## Data Availability

The glycan bead pull-down mass spectrometry data have been deposited to the ProteomeXchange Consortium via the PRIDE (Perez-Riverol et al, 2022) partner repository with the identifier PXD079900. The source data of this paper are collected in the following database record: biostudies:S-SCDT-10_1038-S44318-026-00911-z.
