# Supplementary material for: Clustering of host N-glycans by the microneme MIC1/4/6 complex licenses Toxoplasma rhoptry discharge
Source: EMBO J. 2026 Sep 25;45(20):7070–102. doi: 10.1038/s44318-026-00911-z (PMC13672358; doi:10.1038/s44318-026-00911-z)
Supplement: Supplementary file 1 [file 44318_2026_911_MOESM1_ESM.pdf]

THE EMBO JOURNAL

Clustering of host N-glycans by the microneme MIC1/4/6 complex licenses Toxoplasma rhoptry discharge

Dylan Valleau, Mudita Goyal, Justin Roberts, Frances Male, Gary Ward, and Sebastian Lourido

Corresponding author: Sebastian Lourido (lourido@wi.mit.edu)

Review Timeline:

Submission Date:	4th Dec 25
Editorial Decision:	14th Jan 26
Revision Received:	13th Jul 26
Editorial Decision:	17th Aug 26
Revision Received:	26th Aug 26
Accepted:	31st Aug 26

Editor: Ieva Gailite

Transaction Report:

(Note: With the exception of the correction of typographical or spelling errors that could be a source of ambiguity, letters and reports are not edited. Depending on transfer agreements, referee reports obtained elsewhere may or may not be included in this compilation. Referee reports are anonymous unless the Referee chooses to sign their reports.)

Dear Sebastian,

Thank you for submitting your manuscript for consideration by the EMBO Journal. I sincerely apologise for the protracted assessment process for your manuscript due to delays in report submission. We have now received comments from three reviewers, which are included below for your information.

As you will see, all reviewers are generally positive in their assessment and appreciate the quality of the study and its contribution of the study to the research field. At the same time, reviewers #1 and #2 indicate that revisions would be needed to provide further evidence for the specific disruption of the rhoptry discharge vs adhesion or other invasion steps and to exclude unspecific defects affecting rhoptry discharge upon disruption of N-glycosylation and cholesterol biosynthesis. Furthermore, reviewer #2 requests a clarification of the distinct phenotypic effects of MIC1, MIC4 and MIC6 in the context of their functional interdependence.

Based on the generally positive assessments of reviewers, I would like to invite you to address their comments in a revised manuscript. I think that it would be useful to discuss the revision in more detail via email or phone/videoconferencing - please let me know which option you prefer.

We generally allow three months as standard revision time, which can be extended to six months in the case of major revisions. Should you foresee a problem in meeting this deadline, please let us know in advance to discuss an extension.

As a matter of policy, competing manuscripts published during this period will not negatively impact on our assessment of the conceptual advance presented by your study. However, please contact me as soon as possible upon publication of any related work to discuss the appropriate course of action.

When preparing your letter of response to the referees' comments, please bear in mind that this will form part of the Review Process File and will therefore be available online to the community. For more details on our Transparent Editorial Process, please visit our website: https://link.springer.com/partners/embo-press/editorial-policies. Please also see the attached instructions for further guidelines on preparation of the revised manuscript.

Please feel free to get in touch if have any further questions regarding the revision. Thank you for the opportunity to consider your work for publication, and I look forward to discussing your revision with you.

With best wishes,

Ieva

Ieva Gailite, PhD
Senior Scientific Editor
The EMBO Journal
Meyerhofstrasse 1
D-69117 Heidelberg
Tel: +4962218891309
i.gailite@embojournal.org

Instructions for preparing your revised manuscript:

Read our guidance for manuscript revisions and related editorial policies: https://link.springer.com/journal/44318/submission-guidelines#cms-Revised-submissions

Please make sure you upload a letter of response to the referees' comments together with the revised manuscript.

Please also check that the title and abstract of the manuscript are brief, yet explicit, even to non-specialists.

When assembling figures, please refer to our figure preparation guideline in order to ensure proper formatting and readability in print as well as on screen:
https://media.springernature.com/original/springer-cms/rest/v1/content/27825798/data/v1

At EMBO Press we ask authors to provide source data for the main manuscript figures. You will receive a separate email with instructions for providing source data with your revised manuscript, including how to upload and organize the files.

IMPORTANT: When you send the revision we will require
- a point-by-point response to the referees' comments, with a detailed description of the changes made (as a word file).
- a word file of the manuscript text.
- individual production quality figure files (one file per figure)
- a complete author checklist
- Expanded View files (replacing Supplementary Information)
- a Reagents and Tools Table as part of the Methods section

Please remember: Digital image enhancement is acceptable practice, as long as it accurately represents the original data and conforms to community standards. If a figure has been subjected to significant electronic manipulation, this must be noted in the figure legend or in the 'Materials and Methods' section. The editors reserve the right to request original versions of figures and the original images that were used to assemble the figure.

We realize that it is difficult to revise to a specific deadline. In the interest of protecting the conceptual advance provided by the work, we recommend a revision within 3 months (14th Apr 2026). Please discuss the revision progress ahead of this time with the editor if you require more time to complete the revisions.

Referee #1:

General summary

Rhoptries are apical secretory organelles found in all apicomplexan parasites and are essential for host cell invasion and subversion of host cellular functions. Rhoptry secretion requires prior microneme secretion and contact with host cell factors, which remain poorly defined.

In this study, the authors aimed to identify host factors required for rhoptry discharge in Toxoplasma gondii. Using host-directed genome-wide CRISPR/Cas9 screens combined with a rhoptry discharge reporter, they identify host N-glycosylation and cholesterol as critical determinants of rhoptry secretion. These findings are robustly validated through pharmacological and genetic perturbations and across multiple host cell lines. Furthermore, the authors demonstrate that the MIC1/4/6 micronemal complex mediates interaction with host galactosylated N-glycans, consistent with previous reports on its binding specificity. Overall, this work highlights a key role for host N-glycans and cholesterol in triggering rhoptry discharge and identifies the MIC1/4/6 complex as the parasite ligands linking host glycan recognition to rhoptry secretion.

Although the role of cholesterol and the adhesive properties of the MIC1/4/6 complex have been studied previously, this work provides important mechanistic insight into the overall process. Overall, the study is elegantly conducted, combining multiple complementary approaches and supported by robust controls. The authors deliver a substantial advance in our understanding of rhoptry discharge and the mechanisms underlying this crucial process shared by all apicomplexan parasites.

Major concerns

The study is very well performed, and the data presented are of high caliber, corroborated by the combined use of multiple cell lines as well as complementary pharmacological and genetic approaches. However, there are several points that should be addressed to further strengthen the study:

1. In Figure 2 (panels B, C, D, G, and J), the authors report defects in rhoptry secretion. However, it would be important to also assess parasite invasion under these conditions, as this would provide a more complete characterization of the phenotype and further substantiate the impact of these perturbations on rhoptry secretion and their consequent effects on parasite invasion efficiency.

2. In Figure 2F, the invasion rate of wild-type parasites is reported to be approximately 6%, which appears unusually low. Performing invasion assays in CHO cells under standard conditions would help establish the typical invasion rate in this cell line and allow the reported value to be placed in proper context.

3. In Figure 2H, the authors should include a positive control affecting parasite adherence to validate this assay. For example, a MIC2 knockdown, used later in the study, or treatment with heparan sulphate could serve as appropriate controls.

4. There is an apparent discrepancy regarding the role of sialic acid in rhoptry secretion. Specifically, the results in Figure 2D

and Figures 3B-E suggest that sialic acid has little to no impact, whereas this conclusion appears to be contradicted by the phenotype of MIC4-depleted parasites shown in Figures 4C-E. A clearer explanation for this apparent discrepancy would be necessary, as the claims made at the beginning and end of the manuscript regarding the contribution of sialic acid are somewhat confusing.

Minor concerns

1. In Figure 4, although MIC1/4/6 mutants have been studied previously, it would be important to include invasion assays, similar to those shown in Figure 2, to directly assess the impact of these mutations on parasite invasion efficiency.

2. More generally, for all generated mutants, integration PCRs confirming correct modification of the targeted genetic loci should be provided. In addition, for the conditional knockdown strains, PCR analysis of the locus in the presence and absence of rapamycin should be included to demonstrate that the knockdown system is functioning as intended.

3. In lines 568-569 of the Discussion, the authors state: "we discovered that the MIC1/4/6 microneme complex coordinates host N-glycans and cholesterol to form a microdomain rich in lipids and proteins." While this hypothesis is plausible and supported by previous studies and analogies from mammalian systems, the current manuscript does not present direct experimental evidence of the existence on microdomains to substantiate this claim. The wording should therefore be toned down to better reflect the data presented.

4. The schematic model in Figure 5G does not accurately represent the known stoichiometry of the MIC1/4/6 complex, which has been shown to consist of one MIC6, two MIC1, and two MIC4 subunits (DOI: 10.1038/embor.2008.179).

5. In line 831, the term "sphingolipid GM1 [reference]" is used, but a reference appears to be missing. The authors should add the appropriate citation.

Non-essential suggestion

In lines 506-508, the authors state that MIC1 and MIC4 activate Toll-like receptors to stimulate innate immunity. However, the literature also indicates that rhoptry discharge is a primary mediator of innate immune activation (DOI:10.1093/infdis/jiv250). Since MIC1 and MIC4 influence rhoptry discharge, their effects on innate immunity may be in part indirect. The authors could consider clarifying this point by distinguishing between direct activation of innate immunity by MIC1/MIC4 versus indirect effects via modulation of rhoptry discharge.

Referee #2:

Summary
This manuscript investigates host cell determinants that license rhoptry discharge during Toxoplasma gondii invasion. Using a host-directed genome-wide CRISPR screen combined with extensive genetic, pharmacological, and parasite mutant analyses, the authors identify host N-glycosylation and cholesterol as key pathways required for efficient rhoptry discharge. They further propose that the micronemal MIC1/4/6 complex acts as the parasite factor interfacing with these host pathways by clustering N-glycans and cholesterol into permissive membrane microdomains.
The study is technically strong, the experiments are generally well performed, and the dataset is rich. The conceptual model put forward is interesting and potentially impactful, as it reframes MIC1/4/6 function away from a simple attachment role toward a licensing step for rhoptry secretion. However, several conceptual and interpretational issues remain unresolved, particularly with respect to the specificity of the identified host factors for rhoptry discharge versus upstream invasion steps, and the relationship of the findings to prior work on host glycans and parasite invasion.

Own opinion
I find this study exciting and important for the field of apicomplexan invasion biology. The identification of host N-glycosylation and cholesterol as determinants of rhoptry discharge is novel, and the idea that MIC1/4/6 functions by organizing host membrane microdomains rather than acting solely as a classical adhesin is conceptually appealing. The authors have clearly invested substantial effort into validating their screen hits and integrating host- and parasite-directed genetics.
That said, I believe the manuscript currently overstates the specificity of the identified host factors for rhoptry discharge. In its present form, it remains difficult to fully exclude the possibility that several of the observed phenotypes arise from upstream defects in attachment, apical engagement, or general host cell surface alterations that probabilistically reduce rhoptry discharge. Addressing these points would sharpen and solidify the proposed mechanistic model.

Major concerns
1. Specificity of the screen readout for rhoptry discharge
The logic of the host-directed screen would benefit from clearer justification. Because the CCF4-based readout reports successful rhoptry discharge into host cells, any perturbation that blocks invasion upstream of rhoptry secretion-such as defects

in attachment, apical reorientation, or stable parasite-host engagement-would also score as "green." While the authors argue that the screen is selective for rhoptry discharge, it is not fully clear how broadly upstream invasion defects are excluded, particularly given that many of the identified host genes affect protein trafficking, glycosylation, or lipid metabolism. A clearer discussion of this limitation, together with additional controls, would strengthen the interpretation of the screen. In particular it would be interesting to see what readout is obtained using well described invasion mutants that act upstream of rhoptry discharge.

2. Potential non-specific alterations of the host cell surface

Many of the host pathways identified, notably N-glycosylation and cholesterol biosynthesis, are expected to broadly alter host cell surface composition, membrane organization, and possibly viability. Importantly, loss of N-glycosylation is known to alter plasma membrane organization, protein clustering, and effective membrane tension without overt toxicity. It therefore remains difficult to exclude that the observed rhoptry discharge defects reflect global changes in host cell surface mechanics. Given that the authors themselves discuss membrane mechanics and tension as potential determinants of rhoptry secretion, a more explicit analysis of host cell viability, morphology, and surface integrity would be valuable to rule out non-specific effects.

3. Need to disentangle attachment, invasion, and rhoptry discharge

Although the manuscript includes several parasite mutants as controls, additional benchmarking would strengthen the conclusions. For example, quantitative attachment assays for host cell mutants would be highly informative. Correlating attachment, invasion efficiency, and rhoptry discharge across the same host perturbations would help clarify whether the observed effects are indeed specific to rhoptry discharge or instead reflect a reduced probability of reaching this checkpoint.

4. Interpretation of long-term inhibitor treatments

The inhibitor experiments in Figure 2 are intriguing but raise some concerns because host cells are treated for several days. Quantification of host cell morphology and viability following inhibitor treatment would therefore be helpful. In addition, comparison with more acute perturbations could strengthen the conclusions. Enzymatic approaches such as trypsinization or neuraminidase treatment could provide faster and more selective perturbations.

5. SLC35A2 and SLC35A1 mutants in the context of prior findings

The use of CHO glycosylation mutants is a strength of the study, but the results should be discussed more explicitly in light of previous work. Lec8 (SLC35A2) and Lec2 (SLC35A1) cells were previously examined by Monteiro et al. (1998), who reported that Lec2 cells showed the strongest invasion defect and concluded that host cell sialic acid is important for parasite recognition. In contrast, the present study finds a strong rhoptry discharge defect in SLC35A2 cells and only a modest effect in SLC35A1 cells. While the focus here is rhoptry discharge rather than invasion, both studies rely on similar host glycosylation mutants, and the discrepancy is not addressed. Direct comparison of invasion assays and rhoptry discharge measurements in these mutants would be very informative and would help reconcile these findings.

6. Attachment controls for host glycosylation mutants

The flow-based adhesion assay demonstrating that exogenous galactose does not impair parasite adhesion to human fibroblasts is an important control. However, this does not exclude attachment defects on glycosylation-deficient host cells themselves. Quantifying parasite attachment to SLC35A1- and SLC35A2-deficient host cells would strengthen the conclusion that N-glycans act downstream of initial attachment. Similarly, performing attachment assays with MIC1/4/6 mutant parasites would help exclude the possibility that this complex contributes to both attachment and rhoptry discharge, which could otherwise explain some of the observed phenotypes.

7. Interpretation of MIC1/4/6 genetic interactions

The genetic interaction data involving MIC1, MIC4, and MIC6 raise some unresolved questions. The MIC1/4/6 complex is hierarchically organized: MIC6 functions as a transmembrane escorter required for correct microneme targeting of MIC1 and MIC4, while MIC1 is essential for complex assembly and exit from the early secretory pathway. Accordingly, deletion of either MIC1 or MIC6 prevents proper trafficking of the remaining components to micronemes, effectively abolishing MIC1/4/6 complex function at the parasite surface. In contrast, deletion of MIC4 leaves a MIC1-MIC6 subcomplex intact, with normal trafficking but altered glycan-binding specificity. Given this organization, one would expect MIC6 disruption to phenocopy MIC1 loss more closely than MIC2 disruption. However, as shown in Figure 4C, MIC6 seems to behave differently and more similarly to MIC2, which is unexpected and warrants further clarification. In addition, given that MIC1, MIC4, MIC6, and MIC2 are not essential in vitro and-except for MIC2-only mildly attenuated in vivo, comparison of the conditional knockdowns with available null mutants could help address potential compensatory mechanisms.

Minor issues

1. A brief discussion of potential false negatives in the host-directed screen would help contextualize its sensitivity and specificity.
2. Differences observed between CHO cells and human fibroblasts are interesting but only briefly discussed; additional commentary on cell-type-specific glycosylation differences would be helpful.
3. The use of the term "dominant" for galactosylation may overstate the data given the apparent redundancy with sialic acid recognition; more cautious wording could be considered.
4. Inclusion of explicit host cell viability controls for key inhibitor and knockout conditions would strengthen confidence that the observed phenotypes are not due to toxicity.

Referee #3:

This study addressed a key question in the invasion biology of model apicomplexan parasite Toxoplasma, namely, what host cell factor(s) trigger rhoptry secretion. Rhoptry secretion is a central intermediate step in parasite invasion the follows microneme secretion that mediates host cell attachment and recognition, where rhoptry secretion is essential for initiating invasion and subsequent niche establishment for the parasite in the host. Significantly, it is the first point of delivery of parasite virulence factors into the host cell. While some proteins have been identified in the parasite that are required for rhoptry secretion, little has been know about necessary host factors, or their cognate parasite molecules. Therefore, this study addresses, and provides many important insights to these processes that likely have wide impact on parasite host cell selection and tropism for Toxoplasma and other apicomplexan parasites.

The study has first used genome-wide mutation studies of a model host cell type to test for proteins or pathways that can modulate the success of Toxoplasma rhoptry protein secretion into the host. This broadly identified enrichment for many proteins involved in protein secretion, N-linked glycosylation and sterol synthesis. It would be good if some commentary was offered about which pathways are and are not amenable to gene disruptions in these model host cells, because I wonder if the enrichment metrics are not sensitive to overall tolerance of gene interruption. Nevertheless, thorough follow-up work is performed that further validate the importance of N-glycans and plasma membrane cholesterol levels to rhoptry discharge. These included testing different host cell types, that supported the general conclusions as well as cell-specific differences, independently depleting both select glycoprotein types and cholesterol, and competition with free sugars. The parasite proteins most important in recognising and coordinating the host factors were also elegantly identified and verified, namely the MIC6,1,4 complex. Overall, these studies point to the importance of local cholesterol-enrichment in the host cell for effective rhoptry discharge. Why this is important and for what processes now remains to be determined. But this current work has made a substantial and well-supported contribution to our understandings of this first of the parasite-host interactions used by these parasites.

An outstanding question in the model offered by this report, and I encourage the authors to address (potentially with a figure/schematic), is how the spatial location of the induced cholesterol microdomains is coordinated with the parasite apex. My understanding is that the MIC6,1,4 complex is deployed across the whole of the parasite plasma membrane and so may coordinate N-glycans and cholesterol along their lateral attachment, but rhoptries are discharged apically only. So, how might these two different points of host interaction be reconciled for invasion. While this will likely be inevitably speculative, it would be good to have this model more thoroughly discussed.

If deemed useful, I think the discussion could be condensed where there is quite a bit of repetition of the results. And finally, line 184, stating 'compensation between N and O-glycosylation pathways' is a bit ambiguous and should be further explained. But otherwise, this is an excellent study and I congratulate the authors on their achimenes.

Reviewer comments and responses

Referee #1:

General summary

Rhoptries are apical secretory organelles found in all apicomplexan parasites and are essential for host cell invasion and subversion of host cellular functions. Rhoptry secretion requires prior microneme secretion and contact with host cell factors, which remain poorly defined.

In this study, the authors aimed to identify host factors required for rhoptry discharge in Toxoplasma gondii. Using host-directed genome-wide CRISPR/Cas9 screens combined with a rhoptry discharge reporter, they identify host N-glycosylation and cholesterol as critical determinants of rhoptry secretion. These findings are robustly validated through pharmacological and genetic perturbations and across multiple host cell lines. Furthermore, the authors demonstrate that the MIC1/4/6 micronemal complex mediates interaction with host galactosylated N-glycans, consistent with previous reports on its binding specificity. Overall, this work highlights a key role for host N-glycans and cholesterol in triggering rhoptry discharge and identifies the MIC1/4/6 complex as the parasite ligands linking host glycan recognition to rhoptry secretion.

Although the role of cholesterol and the adhesive properties of the MIC1/4/6 complex have been studied previously, this work provides important mechanistic insight into the overall process. Overall, the study is elegantly conducted, combining multiple complementary approaches and supported by robust controls. The authors deliver a substantial advance in our understanding of rhoptry discharge and the mechanisms underlying this crucial process shared by all apicomplexan parasites.

Major concerns

The study is very well performed, and the data presented are of high caliber, corroborated by the combined use of multiple cell lines as well as complementary pharmacological and genetic approaches. However, there are several points that should be addressed to further strengthen the study:

1. In Figure 2 (panels B, C, D, G, and J), the authors report defects in rhoptry secretion. However, it would be important to also assess parasite invasion under these conditions, as this would provide a more complete characterization of the phenotype and further substantiate the impact of these perturbations on rhoptry secretion and their consequent effects on parasite invasion efficiency.

In the previous version of the manuscript, we focused on rhoptry discharge instead of assessing invasion, since it is generally assumed that perturbations affecting rhoptry discharge also impact invasion (Frénal *et al*, 2017; Cova *et al*, 2022). However, we appreciate the importance of confirming the impact of the studied host perturbations on the overall process of invasion directly.

We have now conducted field-standard immunofluorescent invasion assays (e.g. Cérède *et al*, 2005) for kifunensine-treated host cells (**Figure 2D**), CHO KO cells (**Figure 3E**), exogenously added sialic acid and galactose (**Figure 3H**), and cholesterol removal with add-back (**Figure 2G**), relating to original rhoptry discharge assays in Figure 2 panels B, C, D, G, and J, respectively. As expected, all of the perturbations

that affect rhoptry discharge also had a significant effect on the invasion capability of *T. gondii*, consistent with the essentiality of rhoptry discharge to host cell entry.

2. In Figure 2F, the invasion rate of wild-type parasites is reported to be approximately 6%, which appears unusually low. Performing invasion assays in CHO cells under standard conditions would help establish the typical invasion rate in this cell line and allow the reported value to be placed in proper context.

In the perforation assay, the parasites are allowed to adhere to host cells in a microfluidic chamber in "Endo" buffer, which enables attachment but not invasion. The media is then replaced with live-cell imaging solution, which is a medium permitting invasion, and the parasites are monitored for just over 3 minutes. Two factors contribute to the apparently low level of 6% invasion: (1) the 3-minute window is much shorter than typical invasion assays, which allow invasion to occur over 10–30 minutes; and (2) the switch from Endo buffer is necessary for reproducible and synchronized perforation and invasion but is generally less permissive to invasion than other approaches (Endo *et al*, 1987; Kafsack *et al,* 2003; Male *et al,* 2025). Conventional invasion assays also undergo significantly more washing during immunofluorescence microscopy after fixation, which largely washes away extracellular parasites, so the invasion rate is alternatively calculated as intracellular parasites per host cell, making the two approaches difficult to compare directly. We have also now additionally performed a conventional immunofluorescence inside/outside invasion assay with these cell lines (**Figure 3E**). This approach reveals that both cell lines KOs have an significant impact on invasion rates.

3. In Figure 2H, the authors should include a positive control affecting parasite adherence to validate this assay. For example, a MIC2 knockdown, used later in the study, or treatment with heparan sulphate could serve as appropriate controls.

This is an excellent point and we agree that our earlier attachment assays lacked a true positive control limiting attachment. We have additionally improved and refined our attachment assay, optimizing it such that we can detect an effect from the knockdown of MIC2. MIC2 is the bona fide *T. gondii* TRAP-family orthologue, which is the most well-established family of microneme proteins involved in parasite attachment throughout apicomplexans (Carruthers *et al,* 2000; Gras *et al*, 2017). Using this updated attachment protocol, we demonstrated that MIC2 knockdown quantitatively impacts parasite attachment (while loss of MIC1 has no effect; **Figure 5F-G**). We have similarly updated the attachment assay with exogenous galactose using this improved experimental setup and included heparin sulfate as a positive control (**Figure 3I-J**). To ensure accurate assessment of attachment, we used equimolar addition of the enantiomer L-galactose (which does not affect rhoptry discharge; **Figure 3F**). Consistent with our previous data, D-galactose has no effect while heparin addition clearly inhibits parasite attachment.

4. There is an apparent discrepancy regarding the role of sialic acid in rhoptry secretion. Specifically, the results in Figure 2D and Figures 3B-E suggest that sialic acid has little to no impact, whereas this conclusion appears to be contradicted by the phenotype of MIC4-depleted parasites shown in Figures 4C-E. A clearer explanation for this apparent discrepancy would be necessary, as the claims made at the beginning and end of the manuscript regarding the contribution of sialic acid are somewhat confusing.

Throughout our rhoptry discharge assays sialic acid perturbation consistently has a lesser impact than galactose. An important aspect of this is that for many glycans, including human N-glycans, sialic acid is typically attached to galactose. When N-glycan galactose is removed by SLC35A2 knockout, this additionally leads to loss of N-glycan sialic acids; the new schematic found in **Figure 3A** illustrates this. As noted by the reviewer, the MIC4 cKD experiments provide the key to address this intertwining of galactose and sialic acid binding, particularly **Figure 5C** (previously Figure 4C). When MIC4 is knocked down and galactose binding is consequently disrupted, sialic acid–containing N-glycans play the dominant role. Thus, knockout of SLC35A1—and loss of surface N-glycans that contain sialic acid—has a dramatic impact on rhoptry discharge by MIC4-deficient parasites. We agree that this is a crucial and subtle point, and have attempted to clarify the wording in the results and discussion to more clearly express this key distinction.

Minor concerns

1. In Figure 4, although MIC1/4/6 mutants have been studied previously, it would be important to include invasion assays, similar to those shown in Figure 2, to directly assess the impact of these mutations on parasite invasion efficiency.

We agree that it is useful to benchmark the overall invasion rate for these strains. These results can now be found in **Figure 5H**. The disruption of the MIC1/4/6 complex shows the same general trend on overall invasion rates as for rhoptry discharge assays, with MIC1 and MIC6 loss having a similar significant impact on invasion and MIC4 knockdown having a more modest effect on wild-type host cells.

An interesting note is that the MIC1/4/6 knockdowns have a more moderate effect on invasion than rhoptry discharge. A potential reason for this is that motile parasites (i.e. in invasion assays) have more opportunities to encounter an optimal host cell microsurface to successfully discharge rhoptries and invade. By contrast, parasites are prevented from moving in rhoptry discharge experiments (by treatment with mycalolide B or MyoH knockdown), so those assays measure discharge at the random sites of contact the parasites land upon, making them more dependent on active reshaping of the local host cell membrane by MIC1/4/6. Another important point of contrast is the MIC2 cKD strain, which has an inverse trend compared to the MIC1/4/6 complex: loss of MIC2 has a greater relative impact in invasion assays and attachment assays than rhoptry discharge assays (**Figure 5C-H**). This is consistent with the published role for MIC2, where it mediates overall attachment but not the downstream steps of the invasion process—notably, rhoptry discharge (e.g. Gras *et al.* 2017). Together this also suggests that inhibition of the parasite actomyosin system in our rhoptry discharge assays limits the impact of attachment and motility, as expected. However, it is worth noting the moderate impact of MIC2 loss detected in rhoptry discharge experiments (**Figure 5C-E**) implies sufficient parasite attachment is also beneficial for rhoptry discharge, and highlights the difficulties in fully untangling the different stages of the invasion process. Overall, these invasion results are in line with our model for the rhoptry-specific role of the MIC1/4/6 complex (**Figure 6G**).

2. More generally, for all generated mutants, integration PCRs confirming correct modification of the targeted genetic loci should be provided. In addition, for the conditional knockdown strains, PCR analysis of the locus in the presence and absence of rapamycin should be included to demonstrate that the knockdown system is functioning as intended.

We agree that this is an important validation to include, particularly given the lack of epitope tags or specific antibodies available for MIC4 and MIC6. We have supplied a more comprehensive schematic to illustrate how the strains were made and validated by PCR, as well as for the expected PCR products before and after rapamycin-induced DiCre excision (**Figure EV5A**). Agarose gel images of the corresponding integration and excision analysis PCRs can be found in **Figure EV5B–C**, which demonstrate that the knockdown strains are correctly made and respond as expected to rapamycin treatment.

3. In lines 568-569 of the Discussion, the authors state: "we discovered that the MIC1/4/6 microneme complex coordinates host N-glycans and cholesterol to form a microdomain rich in lipids and proteins." While this hypothesis is plausible and supported by previous studies and analogies from mammalian systems, the current manuscript does not present direct experimental evidence of the existence of microdomains to substantiate this claim. The wording should therefore be toned down to better reflect the data presented.

We are glad the reviewer appreciates that we put forth a plausible model that is supported by the evidence and analogy to other studies, although conclusive direct evidence of these microdomains is still needed. Therefore, we have updated the wording of this statement in the discussion to better reflect the stated model is a possibility which will require future, technically challenging microscopic and model membrane experiments to concretely establish.

4. The schematic model in Figure 5G does not accurately represent the known stoichiometry of the MIC1/4/6 complex, which has been shown to consist of one MIC6, two MIC1, and two MIC4 subunits (DOI: 10.1038/embor.2008.179).

For clarity, we did not include the complex stoichiometry in our diagrams of the MIC1/4/6 complex (**Figures 5B and 6G**). We thought it would be difficult to illustrate the function of individual complex members if the stoichiometry were pictured, and the structure of the complex is not the focus of this work. Nevertheless, to avoid confusion we have noted that, for simplicity, the diagram does not display the expected oligomerization and stoichiometry expected for MIC1/4/6 from prior work.

5. In line 831, the term "sphingolipid GM1 [reference]" is used, but a reference appears to be missing. The authors should add the appropriate citation.

Thank you to the reviewer for catching this oversight, which has now been amended.

Non-essential suggestion

In lines 506-508, the authors state that MIC1 and MIC4 activate Toll-like receptors to stimulate innate immunity. However, the literature also indicates that rhoptry discharge is a primary mediator of innate immune activation (DOI:10.1093/infdis/jiv250). Since MIC1 and MIC4 influence rhoptry discharge, their effects on innate immunity may be in part indirect. The authors could consider clarifying this point by distinguishing between direct activation of innate immunity by MIC1/MIC4 versus indirect effects via modulation of rhoptry discharge.

TLR-mediated innate immune signaling was shown to be activated by the presence of recombinant MIC1 and MIC4 proteins in the absence of parasites (Sardinha-Silva *et al*, 2019). While this separates

MIC1's and MIC4's ability to activate TLRs—and the subsequent TLR-dependent innate immunity signaling—from the act of rhoptry discharge, it is likely both routes of innate-immune activation occur during *in vivo* infection by live parasites. This distinction was not stated clearly in our original text, and we have updated the discussion to better reflect this full context, including a citation for the referenced manuscript.

Referee #2:

Summary

This manuscript investigates host cell determinants that license rhoptry discharge during Toxoplasma gondii invasion. Using a host-directed genome-wide CRISPR screen combined with extensive genetic, pharmacological, and parasite mutant analyses, the authors identify host N-glycosylation and cholesterol as key pathways required for efficient rhoptry discharge. They further propose that the micronemal MIC1/4/6 complex acts as the parasite factor interfacing with these host pathways by clustering N-glycans and cholesterol into permissive membrane microdomains.

The study is technically strong, the experiments are generally well performed, and the dataset is rich. The conceptual model put forward is interesting and potentially impactful, as it reframes MIC1/4/6 function away from a simple attachment role toward a licensing step for rhoptry secretion. However, several conceptual and interpretational issues remain unresolved, particularly with respect to the specificity of the identified host factors for rhoptry discharge versus upstream invasion steps, and the relationship of the findings to prior work on host glycans and parasite invasion.

Own opinion

I find this study exciting and important for the field of apicomplexan invasion biology. The identification of host N-glycosylation and cholesterol as determinants of rhoptry discharge is novel, and the idea that MIC1/4/6 functions by organizing host membrane microdomains rather than acting solely as a classical adhesin is conceptually appealing. The authors have clearly invested substantial effort into validating their screen hits and integrating host- and parasite-directed genetics.

That said, I believe the manuscript currently overstates the specificity of the identified host factors for rhoptry discharge. In its present form, it remains difficult to fully exclude the possibility that several of the observed phenotypes arise from upstream defects in attachment, apical engagement, or general host cell surface alterations that probabilistically reduce rhoptry discharge. Addressing these points would sharpen and solidify the proposed mechanistic model.

Major concerns

1. Specificity of the screen readout for rhoptry discharge

The logic of the host-directed screen would benefit from clearer justification. Because the CCF4-based readout reports successful rhoptry discharge into host cells, any perturbation that blocks invasion upstream of rhoptry secretion-such as defects in attachment, apical reorientation, or stable parasite-host engagement-would also score as "green." While the authors argue that the screen is selective for rhoptry discharge, it is not fully clear how broadly upstream invasion defects are excluded, particularly

given that many of the identified host genes affect protein trafficking, glycosylation, or lipid metabolism. A clearer discussion of this limitation, together with additional controls, would strengthen the interpretation of the screen. In particular it would be interesting to see what readout is obtained using well described invasion mutants that act upstream of rhoptry discharge.

By relying on knockdown of MyoH in our screening strain, and monitoring only rhoptry discharge rather than presence or absence of intracellular parasites, we attempted to remove genes crucial to downstream actomyosin-driven invasion and establishment of the parasitophorous vacuole. To the reviewer's point, there is arguably no way to fully exclude effects from attachment and apical reorientation in such a screen, and our analysis of MIC1/4/6 and MIC2 mutants suggests attachment and rhoptry discharge are intertwined processes (**Figure 5C-H**). Indeed, parasites that discharge rhoptries will be better attached to host cells through the moving junction (Lamarque et al. 2014), which expresses the continuum of adhesive interactions involved in the process of invasion. Additionally, many host genes likely have pleiotropic roles, and could have multiple potential mechanistic impacts affecting our readout of rhoptry discharge. To better communicate these limitations of our screening approach, we have reworded and softened the language used to describe our screen in the results and discussion. These changes should better reflect that despite our attempts to focus the screen on rhoptry discharge, we cannot fully exclude all other aspects of host and pathogen biology, and that the screen must be interpreted within that context. However, while some limitations to our screening approach are present, the function of the screen is to narrow the focus to a potential set of host pathways, and a central goal of our follow-up work (**Figures 2–6**) was to establish if these screen hits are indeed crucial to rhoptry discharge. Especially with the improved attachment assays and invasion assays included in this updated manuscript, it is clear that MIC1/4/6 interactions with N-glycans and cholesterol specifically affect rhoptry discharge in manners that are distinct from the effects of MIC2.

We further agree that it would be interesting to do further screening with strains that have well-described invasion mutants of interest to help disentangle these complex host-parasite interactions. However, performing multiple additional host-directed screens with various parasite mutants or additional controls is an enormous undertaking that is outside of the scope of the current work.

2. Potential non-specific alterations of the host cell surface

Many of the host pathways identified, notably N-glycosylation and cholesterol biosynthesis, are expected to broadly alter host cell surface composition, membrane organization, and possibly viability. Importantly, loss of N-glycosylation is known to alter plasma membrane organization, protein clustering, and effective membrane tension without overt toxicity. It therefore remains difficult to exclude that the observed rhoptry discharge defects reflect global changes in host cell surface mechanics. Given that the authors themselves discuss membrane mechanics and tension as potential determinants of rhoptry secretion, a more explicit analysis of host cell viability, morphology, and surface integrity would be valuable to rule out non-specific effects.

This is an important point, and reflects the difficulty in disentangling the various aspects of the apicomplexan invasion process when they depend on core features of mammalian cell biology. On the topic of membrane tension and mechanics, we think these are likely determinants of efficient rhoptry discharge, but we do not expect to be able to fully resolve those complex questions in the scope of this manuscript.

In this work, we attempted to exclude pleiotropic effects on host biology by using multiple avenues of host perturbation. We used host cell knockouts of sialic acid and galactose transport (SLC35A1 and SLC35A2), and chemical methods targeting specifically N-glycans (i.e. kifunensine). Cholesterol removal and add-back were done using acute, short manipulations. A crucial set of experiments with limited host cell manipulation are those adding exogenous sugars (e.g. **Figure 3F-G, 4B-E, 5D**). In these experiments, host cells have no perturbations prior to addition of parasites, limiting the likelihood of global defects. However, we appreciate that it is important to fully understand the toxicity and specificity of all the host perturbations used to ensure accurate and confident interpretation of the manuscript.

To ensure that our treatments are effective while not being detrimental to host cell viability, we have greatly expanded our phenotypic characterization of host cell knockouts (CHO cells) and chemical treatments (kifunensine, BAG, eliglustat, MβCD & cholesterol reconstitution). This includes an expanded set of host cell-surface glycosylation analysis by fluorescent lectin flow cytometry, validation of viability/membrane permeability by monitoring the ability of cells to exclude the cell-impermeable DNA dye DRAQ7 from the nucleus, and DIC microscopy showing consistent cell morphology (**Figure EV2A-J**, and **Figure EV3D-H**). Together, these validation experiments show that the various chemical and genetic treatments do not affect viability while having the intended effects on host cell glycans.

3. Need to disentangle attachment, invasion, and rhoptry discharge

Although the manuscript includes several parasite mutants as controls, additional benchmarking would strengthen the conclusions. For example, quantitative attachment assays for host cell mutants would be highly informative. Correlating attachment, invasion efficiency, and rhoptry discharge across the same host perturbations would help clarify whether the observed effects are indeed specific to rhoptry discharge or instead reflect a reduced probability of reaching this checkpoint.

We agree with the reviewer on the value of comparing attachment, invasion, and rhoptry discharge, when possible. Invasion assays have now been performed for all key perturbations: kifunensine inhibition of N-glycosylation (**Figure 2D**); cholesterol manipulation (**Figure 2G**); CHO KO cells (**Figure 3E**); inhibition by exogenous sugars (**Figure 3H**); and for all cKD parasite strains (**Figure 5H**). Crucially, for all conditions where we see a rhoptry defect, we also see an invasion defect, consistent with the well established hierarchy of rhoptry discharge preceding invasion. We have additionally updated our attachment assays and quantified the impact of MIC1/4/6 loss compared to MIC2 knockdown (**Figure 5F-G**), as well as investigated the impact of exogenous galactose on attachment with improved controls (**Figure 3I-J**). We believe these additional experiments strongly support our proposed model and confirm the impact of these perturbations on rhoptry discharge specifically.

The most crucial of these experiments are the invasion assays and quantitative attachment assays performed with the MIC1/4/6 and MIC2 cKD strains. These confirmed the previously established overall invasion defect for this complex (**Figure 5H**) (Marchant *et al*, 2012, Friedrich *et al*, 2009, Sawmynaden *et al*, 2008). For MIC2, which is previously established to be important for attachment—and consequently, invasion—we observe knockdown leads to the expected defect in invasion. In our redeveloped quantitative attachment assays (**Figure 5F-G**), MIC2 knockdown also leads to an attachment defect. This supports a model in which the impact of MIC2 on rhoptry secretion and invasion reflects a decreased ability of reaching sufficient attachment, consistent with previous work (e.g. Gras *et al.* 2017). By contrast, for MIC1/4/6 complex disruption via MIC1 knockdown we saw no defect in attachment, as

expected (Cérède et al, 2005). Additionally, loss of MIC1/4/6 has a stronger defect in rhoptry discharge than loss of MIC2, consistent with the role of the MIC1/4/6 complex occurring at the stage of rhoptry discharge. Please note that as these quantitative attachment assays are time consuming and laborious, we were forced to selectively perform them with just the MIC1 cKD, as MIC1 knockdown is well established to disrupt the function of the complex as a whole.

In regards to testing host cell perturbations in attachment, CHO cells do not readily form contact-inhibited confluent monolayers, and their morphology is more rounded, together making flow-based adhesion assays that compare knockout cell lines difficult and extremely variable. Therefore we instead quantified the impact of exogenously added galactose in the media on parasite attachment to HFF cells using our improved attachment assay. In this experiment, the enantiomer L-galactose was used as a negative control, as it has identical viscosity effects on the medium as D-galactose. Heparin sulfate was also added for a positive control that is well established to block attachment (Carruthers *et al*, 2000). This found exogenously added galactose, despite its effects on invasion and rhoptry discharge, does not affect attachment (**Figure 3I-J**).

The pertinent paragraphs of the results and discussion have been updated to reflect the additional experiments. Overall, we feel that these benchmarking attachment and invasion assays clearly support our model where—distinct from the role of MIC2—galactose-containing N-glycans are interacted with via MIC1/4/6 to support rhoptry discharge separately from general host cell attachment.

4. Interpretation of long-term inhibitor treatments

The inhibitor experiments in Figure 2 are intriguing but raise some concerns because host cells are treated for several days. Quantification of host cell morphology and viability following inhibitor treatment would therefore be helpful. In addition, comparison with more acute perturbations could strengthen the conclusions. Enzymatic approaches such as trypsinization or neuraminidase treatment could provide faster and more selective perturbations.

The treatment times and concentrations were titrated and are in line with established use of these reagents in other studies. However, we agree it is important to rule out viability and broad morphology changes as contributors to the observed phenotypes. As outlined above, we have now performed viability analysis and DIC imaging for cell morphology after these treatments. This shows consistent viability and morphology across the extended treatments with BAG, kif, and elig. We additionally performed more detailed cell surface staining to fully demonstrate the effectiveness and specificity of these pharmacological treatments (**Figure EV2A-H**).

We additionally performed a treatment time-course with kif, to address how acute vs extended treatments affect rhoptry discharge and invasion (**Figure 2D**). We find that kif treatment has its maximum effect on invasion and rhoptry discharge between 3 and 5 days, consistent with the timepoints used in our other experiments with BAG and eliglustat. Furthermore, this increase in effect over time supports a model where kif does not have a direct inhibitory effect on rhoptry discharge. Rather, kif blocks synthesis of mature complex- and hybrid-type N-glycans in growing/dividing cells, in turn causing a defect in both rhoptry discharge and parasite invasion. This experiment and the above validations have now been incorporated into the manuscript.

In regards to enzymatic treatment of host cells with reagents like neuraminidase and trypsin, we have attempted this approach previously. However, we found that achieving the penetrance required while retaining cell viability is more difficult and inconsistent than with the pharmacological and genetic approaches presented in this manuscript. This is consistent with the paper mentioned below in comment #5 (Monteiro *et al*, 1998), in which the effect of Lec2 (ΔSLC35A1) CHO cells on parasite invasion had a greater effect than neuraminidase treatment. However, a potential direction for future work could be to combine these enzymatic treatments with specific genetic backgrounds to combinatorally tease apart the order and sufficiency of host interactions. We believe that the set of complementary approaches employed in our current work is sufficient to support our conclusions without the proposed enzymatic treatments; more specifically, we would point that the exogenous sugars provide an acute treatment that shows expected interactions with genetic perturbations in the host and parasite.

5. SLC35A2 and SLC35A1 mutants in the context of prior findings

The use of CHO glycosylation mutants is a strength of the study, but the results should be discussed more explicitly in light of previous work. Lec8 (SLC35A2) and Lec2 (SLC35A1) cells were previously examined by Monteiro et al. (1998), who reported that Lec2 cells showed the strongest invasion defect and concluded that host cell sialic acid is important for parasite recognition. In contrast, the present study finds a strong rhoptry discharge defect in SLC35A2 cells and only a modest effect in SLC35A1 cells. While the focus here is rhoptry discharge rather than invasion, both studies rely on similar host glycosylation mutants, and the discrepancy is not addressed. Direct comparison of invasion assays and rhoptry discharge measurements in these mutants would be very informative and would help reconcile these findings.

This is a very good point and reflects the subtle differences in which these assays are performed. As discussed in response to Referee #1, the host permeabilization assay presented in **Figure 3D–E** is performed in microfluidic chambers where parasites are first allowed to attach under buffer conditions that prevent invasion. Non-attached parasites are then washed away when the buffer is replaced with live-cell imaging solution in which the parasites are able to invade. The start of the experiment is synchronized by this media swap, which allows accurate tracking of perforation and invasion events, reported as the proportion of parasites present. This buffer swap and subsequent removal of non-attached parasites limits the degree to which attachment is measured as a contributor to invasion and perforation. In typical inside/outside invasion assays, such as those used by Monteiro *et al*., or our prior publications (Valleau *et al,* 2023, Sidik *et al.* 2016), equal parasite concentrations are added to host cells, typically allowed to invade for longer periods than in the permeabilization assay (10–30 minutes compared to 3 minutes), and quantified as the proportion of intracellular parasites relative number of host cells.

To confirm the host cells are behaving as expected from the literature, we also performed typical invasion assays. In this experiment, the trends are consistent with Monteiro *et al*., with Lec2 (SLC35A1 KO) showing a greater effect on invasion than Lec8 (SLC35A2) host cells (**Figure 3E**). Together, given the difference in the perforation vs. classical invasion experiments, we believe our results show that sialic acid has multiple roles: participating in attachment as well as in being recognized by MIC1. This is supported by prior reports of Lec2 (SLC35A1 KO) cells having a defect in parasite adhesion (Baba *et al*, 2015). By contrast, galactose is not required for attachment (**Figure 3I-J**), but is important for rhoptry

discharge (**Figure 3F-G**), and invasion (**Figure 3H**; also reported by Friedrich *et al*, 2009). We have updated the text of the Results and Discussion sections to incorporate this new data and added a brief discussion of the distinctions and consequences in experimental setup between the perforation/invasion and standard invasion assays.

6. Attachment controls for host glycosylation mutants

The flow-based adhesion assay demonstrating that exogenous galactose does not impair parasite adhesion to human fibroblasts is an important control. However, this does not exclude attachment defects on glycosylation-deficient host cells themselves. Quantifying parasite attachment to SLC35A1- and SLC35A2-deficient host cells would strengthen the conclusion that N-glycans act downstream of initial attachment. Similarly, performing attachment assays with MIC1/4/6 mutant parasites would help exclude the possibility that this complex contributes to both attachment and rhoptry discharge, which could otherwise explain some of the observed phenotypes.

As discussed above in response to point #3, in our attempts to disentangle attachment, rhoptry discharge, and invasion, we have now performed improved attachment assays with additional controls (**Figure 3I-J**), and compared MIC1 and MIC2 knockdown parasites (**Figure 5F-G**). This showed that addition of galactose does not block attachment, nor are the interactions mediated by the MIC1/4/6 complex. Collectively these results strongly support the conclusion that MIC1/4/6 galactose-dependent interactions are involved in rhoptry discharge specifically. These attachment assays are time-consuming and laborious, and due to their more rounded morphology and their inability to form contact-inhibited monolayers in flow cells, it has not been possible to perform quantitative flow assays comparing CHO cells lines.

7. Interpretation of MIC1/4/6 genetic interactions

The genetic interaction data involving MIC1, MIC4, and MIC6 raise some unresolved questions. The MIC1/4/6 complex is hierarchically organized: MIC6 functions as a transmembrane escorter required for correct microneme targeting of MIC1 and MIC4, while MIC1 is essential for complex assembly and exit from the early secretory pathway. Accordingly, deletion of either MIC1 or MIC6 prevents proper trafficking of the remaining components to micronemes, effectively abolishing MIC1/4/6 complex function at the parasite surface. In contrast, deletion of MIC4 leaves a MIC1-MIC6 subcomplex intact, with normal trafficking but altered glycan-binding specificity. Given this organization, one would expect MIC6 disruption to phenocopy MIC1 loss more closely than MIC2 disruption. However, as shown in Figure 4C, MIC6 seems to behave differently and more similarly to MIC2, which is unexpected and warrants further clarification. In addition, given that MIC1, MIC4, MIC6, and MIC2 are not essential in vitro and-except for MIC2-only mildly attenuated in vivo, comparison of the conditional knockdowns with available null mutants could help address potential compensatory mechanisms.

The reviewer is correct about the assembly of the complex and the formation of the MIC6/MIC1 subcomplex in the absence of MIC4. The comparatively more modest effect of MIC6 knockdown may result from residual MIC6 expression or the potential stability of a MIC1/MIC4 subcomplex capable of partially nucleating microdomains for rhoptry discharge despite the absence of the MIC6 membrane tether. It is important to note that while SLC35A2 KO still suppressed rhoptry discharge of MIC6 and MIC2 knockdown parasites (**Figure 5C**), the relative ratio of the effect is measurably different.

Specifically, MIC6 knockdown parasites discharge rhoptries at a similar magnitude into wildtype and SLC35A2 KO host cells, while MIC2 knockdown parasites were more significantly affected by the loss of SLC35A2, following a similar pattern to WT parasites (DiCre). This pattern of MIC2 rhoptry discharge held true for the CHO KOs, exogenous addition of sialic acid/galactose, and kif treatment (**Figure 5C–E**), which together reveal an additive interaction between MIC2 knockdown and galactose-containing N-glycans, interpreted as perturbations in parallel pathways. By contrast, MIC6 knockdown displayed non-additivity more similar to the phenotype of MIC1 knockdown throughout these assays.

Regarding the null mutants, we felt that using conditional knockdowns would limit the likelihood of selecting for adaptations over time that would confound interpretation of the results. This phenomenon of adaptation is observed for other invasion-related proteins, such as AMA1 knockout, which leads to the upregulation of alternative paralogous AMA/RON2 pairs typically expressed in other parasite life-stages (Lamarque *et al*, 2014). Indeed, we profiled previously generated ΔMIC1 and ΔMIC4 *T. gondii* null mutants (Sardinha-Silva et al, 2019) using quantitative proteomics and found that both knockouts had increased levels of MIC13, a bradyzoite-specific paralogue of MIC1. Thus we felt that conditional knockdown approaches were ideal for this study, additionally allowing us to construct an isogenic panel of mutants that included MIC2 as a control.

Minor issues

1. A brief discussion of potential false negatives in the host-directed screen would help contextualize its sensitivity and specificity.

There are indeed some potential false negatives from this screen, such as dropout of essential genes from the pool prior to the assay, receptor redundancy, and presence of paralogs common to families of surface-exposed proteins. It is also possible that *T. gondii* does not rely on any specific individual factor, making detection difficult in a screening context. In the case of essential genes, we find that there is no correlation between our screen scores and the essentiality, suggesting drop-out prior to data collection is not a major contributor to scores, though it cannot be fully excluded. We have noted this in the Results section describing the screen results and added a plot representing this finding (**Figure EV1B**). We have additionally updated the corresponding Results and Discussion sections to better highlight the potential false-negative of redundancy in expression of extracellular proteins in K562 cells and that *T. gondii* may simply not bind to any one specific protein.

2. Differences observed between CHO cells and human fibroblasts are interesting but only briefly discussed; additional commentary on cell-type-specific glycosylation differences would be helpful.

This is indeed an interesting point of contrast. To our knowledge, these two cell types have not been compared in literature in this regard. In general, comprehensive glycan profiling of cell types lags behind other transcriptomic and proteomic comparative studies, so we are somewhat limited in how we can interpret the differences in CHO and HFF cells. However, there are some potential differences we didn't cover in the original text. CHO cells are reported to lack α2,6-linked sialic acid and instead have a predominance of α2,3-linked glycans (Baba *et al*, 2015; Lee *et al*, 1989), which is the linkage preferentially bound by MIC1. MIC4 knockdown parasites are solely reliant on sialic acid and also discharge rhoptries at a higher relative rate into CHO cells than HFF cells. It is possible that the absence of α2,6-linked sialic acids leads to a higher abundance of α2,3-linked sialic acid and limits *Toxoplasma*'s

dependence on MIC4 to infect “wild-type” CHO cells. Differences in cell type could also stem from overall ratios of complex- and hybrid-type N-glycans to mannose-type N-glycans. We appreciate the reviewer providing us with the opportunity to better contextualize these results and have added these possibilities to the discussion section.

3. The use of the term "dominant" for galactosylation may overstate the data given the apparent redundancy with sialic acid recognition; more cautious wording could be considered.

Thank you for raising this point. Our use of “dominant” was intended to describe how SLC35A2 knockout removes both galactose and sialic acid from N-glycans, since sialic acid attachment requires a preceding galactose moiety. This was unclearly stated, and we have revised this language in paragraph 2 of the discussion to ensure this is precisely conveyed.

4. Inclusion of explicit host cell viability controls for key inhibitor and knockout conditions would strengthen confidence that the observed phenotypes are not due to toxicity.

We have now included host cell viability assays (based on excursion of the DNA-binding dye DRAQ7) for the various host cell conditions tested. Specifically, cholesterol depletion/add-back, kifunensine, BAG, eliglustat, and all used CHO cells (**Figure EV2A-J**, and **Figure EV3D-H**). Crucially, for none of these treatments did we observe a difference in viability compared to controls.

Referee #3:

This study addressed a key question in the invasion biology of model apicomplexan parasite Toxoplasma, namely, what host cell factor(s) trigger rhoptry secretion. Rhoptry secretion is a central intermediate step in parasite invasion the follows microneme secretion that mediates host cell attachment and recognition, where rhoptry secretion is essential for initiating invasion and subsequent niche establishment for the parasite in the host. Significantly, it is the first point of delivery of parasite virulence factors into the host cell. While some proteins have been identified in the parasite that are required for rhoptry secretion, little has been know about necessary host factors, or their cognate parasite molecules. Therefore, this study addresses, and provides many important insights to these processes that likely have wide impact on parasite host cell selection and tropism for Toxoplasma and other apicomplexan parasites.

The study has first used genome-wide mutation studies of a model host cell type to test for proteins or pathways that can modulate the success of Toxoplasma rhoptry protein secretion into the host. This broadly identified enrichment for many proteins involved in protein secretion, N-linked glycosylation and sterol synthesis. It would be good if some commentary was offered about which pathways are and are not amenable to gene disruptions in these model host cells, because I wonder if the enrichment metrics are not sensitive to overall tolerance of gene interruption. Nevertheless, thorough follow-up work is performed that further validate the importance of N-glycans and plasma membrane cholesterol levels to rhoptry discharge. These included testing different host cell types, that supported the general conclusions as well as cell-specific differences, independently depleting both select glycoprotein types and cholesterol, and competition with free sugars. The parasite proteins most important in recognising and coordinating the host factors were also elegantly identified and verified, namely the MIC6,1,4 complex. Overall, these studies point to the importance of local cholesterol-enrichment in the host cell for effective rhoptry discharge. Why this is important and for what processes now remains to be

determined. But this current work has made a substantial and well-supported contribution to our understandings of this first of the parasite-host interactions used by these parasites.

Thank you to the reviewer for their constructive and thorough reading of the manuscript. We have additionally plotted the results of our screen vs. essentiality in K562 cells (**Figure EV1B**), which finds there is minimal correlation between essentiality and the hits in our screen. We have also provided some additional commentary about false negatives that could be present, to better convey the limitations of this approach.

An outstanding question in the model offered by this report, and I encourage the authors to address (potentially with a figure/schematic), is how the spatial location of the induced cholesterol microdomains is coordinated with the parasite apex. My understanding is that the MIC6,1,4 complex is deployed across the whole of the parasite plasma membrane and so may coordinate N-glycans and cholesterol along their lateral attachment, but rhoptries are discharged apically only. So, how might these two different points of host interaction be reconciled for invasion. While this will likely be inevitably speculative, it would be good to have this model more thoroughly discussed.

This is indeed an interesting question that is rather complicated to fully resolve. Microneme proteins, including MIC1/4/6 are concentrated at the apical end of the parasite where they are first secreted. We expect this apically concentrated pool of MIC1/4/6 is involved in engaging host cells for rhoptry discharge. MIC1/4/6 that is translocated beyond the apical end will be less concentrated and eventually shed from the parasite membrane; indeed, we previously found MIC1/4/6 in a proteomic survey of proteins secreted and released from extracellular parasites (Valleau *et al*, 2023). Subsequent microneme secretion events continuously display apically concentrated MIC1/4/6 that can mediate rhoptry discharge. Thus, while MIC1/4/6 can be found along the length of the parasite, we only expect the apical pool of MIC1/4/6 to participate in rhoptry discharge.

If deemed useful, I think the discussion could be condensed where there is quite a bit of repetition of the results. And finally, line 184, stating 'compensation between N and O-glycosylation pathways' is a bit ambiguous and should be further explained. But otherwise, this is an excellent study and I congratulate the authors on their achimenes.

Thank you to the reviewer for their supportive comments. We have modified the wording from line 184 to more precisely state the mechanism of compensation as follows:

> "Since N- and O-glycosylation utilize a shared pool of precursor sugars and enzymes, kif and BAG can alter metabolic flux through these parallel biosynthetic pathways. BAG may therefore promote increased production of N-glycosylation, decreasing the inhibitory impact of kif (Kong & Fujita, 2025; McDonald et al, 2016; Scheper et al, 2023)."

Dear Sebastian,

Thank you for submitting a revised version of your manuscript to The EMBO journal. I sincerely apologise for the protracted assessment process of your manuscript due to summer holidays. Your study has now been seen by two of the original referees, who find that their previous concerns have been sufficiently addressed and now recommend publication of the manuscript.

There now remain only a few editorial points that need to be addressed before I can extend the official acceptance of the manuscript:

1. Please note that it is The EMBO Journal policy for the transcript of the editorial process (containing referee reports and your response letters) to be published as an online supplement to each paper. If you should prefer removal of any referee-only figures included in the point-by-point response(s), e.g. because they may still be used for future publication or because they have been reproduced from published work by others, please do let us know immediately via response email.
More information is available here: https://www.embopress.org/transparent-process#Review_Process
2. Please provide the current email address Mudita Goyal - we received an error message for the provided email address mgoyal02@mit.edu.
3. Please update the order and the headings of the manuscript sections to: Abstract / Introduction / Results / Discussion / Methods / Data Availability / Acknowledgements / Disclosure and Competing Interests Statement / References / Figure Legends / Expanded View Figure Legends.
4. Please remove the dataset legends from the manuscript file and correct the nomenclature in the files of Datasets EV1 and EV2.
5. In the Data Availability section, please add a resolvable URL to the PXD079900 dataset.
6. Our data editors have indicated that the exact p values should be provided in the legends of figures 1C, 2C, D, F, G; 3B, D, G, H; 4B, C, D, E, G, H; 5H, 6C, E; 6C, E; EV4 A-C; EV5F-H.
7. Papers published in The EMBO Journal are accompanied online by a 'Synopsis' to enhance discoverability of the manuscript. It consists of A) a short (1-2 sentences) summary of the findings and their significance, B) 3-4 bullet points highlighting key results (the highlights currently included in the manuscript can be used here) and C) a synopsis image that is 550x300 pixels large (width x height, jpeg or png format). You can either show a model or key data in the synopsis image. Please note that the image size is rather small and that text needs to be readable at the final size.

Please let me know if you have any questions regarding any of these points. You can use the link below to upload the revised files.

Thank you again for giving us the chance to consider your manuscript for The EMBO Journal. I look forward to receiving the final version.

With best wishes,

Ieva

Ieva Gailite, PhD
Senior Scientific Editor
The EMBO Journal
Meyerhofstrasse 1
69117 Heidelberg

We realize that it is difficult to revise to a specific deadline. In the interest of protecting the conceptual advance provided by the work, we recommend a revision within 3 months (15th Nov 2026). Please discuss the revision progress ahead of this time with the editor if you require more time to complete the revisions.

Referee #1:

The revised manuscript has been substantially strengthened and addresses all major and minor points raised by the reviewers. The authors have added important complementary experiments, including invasion assays, improved attachment controls, and genetic validation of mutant strains, which further support the central conclusions of the study. The revised text also

appropriately refines several interpretations and provides a clearer mechanistic model linking host N-glycans, cholesterol, and the MIC1/4/6 complex to rhoptry discharge.

Overall, the work represents a significant advance in our understanding of apicomplexan invasion biology, and I consider the manuscript suitable for acceptance.

Referee #2:

I thank the authors for their careful revision of the manuscript. My major concerns have fully been addressed by combination of new experiments, and more balanced interpretation of the data.

The revised manuscript now includes important additional invasion assays accompanying the rhoptry discharge analyses, and a clearer comparison between attachment, rhoptry discharge, and invasion. In particular, I appreciate the authors' efforts to distinguish the function of the MIC1/4/6 complex from that of MIC2.I have no further major concerns and believe the manuscript represents a significant advance in our understanding of the molecular events governing apicomplexan host cell invasion.

The authors addressed the remaining editorial issues.

Dear Sebastian,

Thank you for addressing the editorial and formatting requests in your manuscript. I am now pleased to inform you that your manuscript has been accepted for publication. Congratulations on a nice study!

Before we forward your manuscript to the publishers, we would like to propose some edits in the manuscript title and synopsis. We have also selected a short blurb that will accompany the title of your manuscript in our online table of contents. Please take a look at the proposed textual changes in the attached text file - any corrections or edits from your side are very welcome.

Your manuscript will be processed for publication by EMBO Press. It will be copy edited and you will receive page proofs prior to publication. Please note that you will be contacted by Springer Nature Author Services to complete licensing and payment information.

You may qualify for financial assistance for your publication charges - either via a Springer Nature fully open access agreement or an EMBO initiative. Check your eligibility: https://link.springer.com/journal/44318/how-to-publish-with-us

Should you be planning a Press Release on your article, please notify the production team via this form in order to coordinate publication and release dates: https://forms.cloud.microsoft/e/eH4A8JVP6g

I look forward to receiving your input on the final text edits soon. Thank you for this interesting contribution to The EMBO Journal!

Best wishes,

Ieva

Ieva Gailite, PhD
Senior Scientific Editor
The EMBO Journal
Meyerhofstrasse 1
69117 Heidelberg

EMBO Press Author Checklist

Corresponding Author Name: Sebastian Lourido	
Journal Submitted to: The EMBO Journal	
Manuscript Number: EMBOJ-2025-123208	

USEFUL LINKS FOR COMPLETING THIS FORM
The EMBO Journal - Author Guidelines
EMBO Reports - Author Guidelines
Molecular Systems Biology - Author Guidelines
EMBO Molecular Medicine - Author Guidelines

Reporting Checklist for Life Science Articles (updated January
This checklist is adapted from Materials Design Analysis Reporting (MDAR) Checklist for Authors. MDAR establishes a minimum set of requirements in transparent reporting in the life sciences (see Statement of Task: 10.31222/osf.io/9sm4x). Please follow the journal's guidelines in preparing your
Please note that a copy of this checklist will be published alongside your article.

Abridged guidelines for figures
1. Data
The data shown in figures should satisfy the following conditions:
- the data were obtained and processed according to the field's best practice and are presented to reflect the results of the experiments in an accurate and unbiased manner.
- ideally, figure panels should include only measurements that are directly comparable to each other and obtained with the same assay.
- plots include clearly labeled error bars for independent experiments and sample sizes. Unless justified, error bars should not be shown for technical
- if n<5, the individual data points from each experiment should be plotted. Any statistical test employed should be justified.
- Source Data should be included to report the data underlying figures according to the guidelines set out in the authorship guidelines on Data

2. Captions
Each figure caption should contain the following information, for each panel where they are relevant:
- a specification of the experimental system investigated (eg cell line, species name).
- the assay(s) and method(s) used to carry out the reported observations and measurements.
- an explicit mention of the biological and chemical entity(ies) that are being measured.
- an explicit mention of the biological and chemical entity(ies) that are altered/varied/perturbed in a controlled manner.
- the exact sample size (n) for each experimental group/condition, given as a number, not a range;
- a description of the sample collection allowing the reader to understand whether the samples represent technical or biological replicates (including how many animals, litters, cultures, etc.).
- a statement of how many times the experiment shown was independently replicated in the laboratory.
- definitions of statistical methods and measures:
  - common tests, such as t-test (please specify whether paired vs. unpaired), simple χ2 tests, Wilcoxon and Mann-Whitney tests, can be unambiguously identified by name only, but more complex techniques should be described in the methods section;
  - are tests one-sided or two-sided?
  - are there adjustments for multiple comparisons?
  - exact statistical test results, e.g., P values = x but not P values < x;
  - definition of 'center values' as median or average;
  - definition of error bars as s.d. or s.e.m.

Please complete ALL of the questions below.
Select "Not Applicable" only when the requested information is not relevant for your study.

Materials

Newly Created Materials	Information included in the manuscript?	In which section is the information available? (Reagents and Tools Table, Materials and Methods, Figures, Data Availability Section)
New materials and reagents need to be available; do any restrictions apply?	Yes	Reagents and Tools Table, Materials and Methods

Antibodies	Information included in the manuscript?	In which section is the information available? (Reagents and Tools Table, Materials and Methods, Figures, Data Availability Section)
For **antibodies** provide the following information:<br>- Commercial antibodies: RRID (if possible) or supplier name, catalogue number and or/clone number<br>- Non-commercial: RRID or citation	Yes	Reagents and Tools Table, Materials and Methods

DNA and RNA sequences	Information included in the manuscript?	In which section is the information available? (Reagents and Tools Table, Materials and Methods, Figures, Data Availability Section)
**Short novel DNA or RNA including primers, probes**: provide the sequences.	Not Applicable	Reagents and Tools Table (inc. sequences), Materials and Methods

Cell materials	Information included in the manuscript?	In which section is the information available? (Reagents and Tools Table, Materials and Methods, Figures, Data Availability Section)
**Cell lines:** Provide species information, strain. Provide accession number in repository **OR** supplier name, catalog number, clone number, and/**OR** RRID.	Yes	Reagents and Tools Table, Materials and Methods
**Primary cultures:** Provide species, strain, sex of origin, genetic modification status.	Yes	Reagents and Tools Table, Materials and Methods
Report if the cell lines were recently **authenticated** (e.g., by STR profiling) and tested for mycoplasma contamination.	Not Applicable	

Experimental animals	Information included in the manuscript?	In which section is the information available? (Reagents and Tools Table, Materials and Methods, Figures, Data Availability Section)
**Laboratory animals or Model organisms:** Provide species, strain, sex, age, genetic modification status. Provide accession number in repository **OR** supplier name, catalog number, clone number, **OR** RRID.	Not Applicable	
**Animal observed in or captured from the field:** Provide species, sex, and age where possible.	Not Applicable	
Please detail **housing and husbandry conditions**.	Not Applicable	

Plants and microbes	Information included in the manuscript?	In which section is the information available? (Reagents and Tools Table, Materials and Methods, Figures, Data Availability Section)
**Plants:** provide species and strain, ecotype and cultivar where relevant, unique accession number if available, and source (including location for collected wild specimens).	Not Applicable	
**Microbes:** provide species and strain, unique accession number if available, and source.	Yes	Reagents and Tools Table, Materials and Methods

Human research participants	Information included in the manuscript?	In which section is the information available? (Reagents and Tools Table, Materials and Methods, Figures, Data Availability Section)
If collected and within the bounds of privacy constraints report on age, sex and gender or ethnicity for all study participants.	Not Applicable	

Core facilities	Information included in the manuscript?	In which section is the information available? (Reagents and Tools Table, Materials and Methods, Figures, Data Availability Section)
If your work benefited from core facilities, was their service mentioned in the acknowledgments section?	Yes	Acknowledgements

Design

Study protocol	Information included in the manuscript?	In which section is the information available? (Reagents and Tools Table, Materials and Methods, Figures, Data Availability Section)
If study protocol has been **pre-registered, provide DOI in the manuscript**. For clinical trials, provide the trial registration number **OR** cite DOI.	Not Applicable	
Report the **clinical trial registration number** (at ClinicalTrials.gov or equivalent), where applicable.	Not Applicable	

Laboratory protocol	Information included in the manuscript?	In which section is the information available? (Reagents and Tools Table, Materials and Methods, Figures, Data Availability Section)
Provide DOI OR other citation details if **external detailed step-by-step protocols** are available.	Not Applicable	

Experimental study design and statistics	Information included in the manuscript?	In which section is the information available? (Reagents and Tools Table, Materials and Methods, Figures, Data Availability Section)
Include a statement about **sample size** estimate even if no statistical methods were used.	Not Applicable	
Were any steps taken to minimize the effects of subjective bias when allocating animals/samples to treatment (e.g. **randomization procedure**)? If yes, have they been described?	Not Applicable	
Include a statement about **blinding** even if no blinding was done.	Not Applicable	
Describe **inclusion/exclusion criteria** if samples or animals were excluded from the analysis. Were the criteria pre-established? If sample or data points were omitted from analysis, report if this was due to attrition or intentional exclusion and provide justification.	Not Applicable	
For every figure, are **statistical tests** justified as appropriate? Do the data meet the assumptions of the tests (e.g., normal distribution)? Describe any methods used to assess it. Is there an estimate of variation within each group of data? Is the variance similar between the groups that are being statistically compared?	Yes	Materials and Methods, Figures, Figure Legends

Sample definition and in-laboratory replication	Information included in the manuscript?	In which section is the information available? (Reagents and Tools Table, Materials and Methods, Figures, Data Availability Section)
In the figure legends: state number of times the experiment was **replicated** in laboratory.	Not Applicable	
In the figure legends: define whether data describe **technical or biological replicates**.	Yes	Materials and Methods

Ethics

Ethics	Information included in the manuscript?	In which section is the information available? (Reagents and Tools Table, Materials and Methods, Figures, Data Availability Section)
Studies involving **human participants**: State details of **authority granting ethics approval** (IRB or equivalent committee(s), provide reference number for approval.	Not Applicable	
Studies involving **human participants**: Include a statement confirming that **informed consent** was obtained from all subjects and that the experiments conformed to the principles set out in the WMA Declaration of Helsinki and the Department of Health and Human Services Belmont Report.	Not Applicable	
Studies involving **human participants: For** publication of **patient photos**, include a statement confirming that consent to publish was obtained.	Not Applicable	
Studies involving experimental **animals**: State details of **authority granting ethics approval** (IRB or equivalent committee(s), provide reference number for approval. Include a statement of compliance with ethical regulations.	Not Applicable	
Studies involving **specimen and field samples:** State if relevant **permits** obtained, provide details of authority approving study; if none were required, explain why.	Not Applicable	

Dual Use Research of Concern (DURC)	Information included in the manuscript?	In which section is the information available? (Reagents and Tools Table, Materials and Methods, Figures, Data Availability Section)
Could your study fall under dual use research restrictions? Please check biosecurity documents and list of **select agents and toxins** (CDC): https://www.selectagents.gov/sat/list.htm	Not Applicable	
If you used a select agent, is the security level of the lab appropriate and reported in the manuscript?	Not Applicable	
If a study is subject to dual use research of concern regulations, is the name of the **authority granting approval and reference number** for the regulatory approval provided in the manuscript?	Not Applicable	

Reporting

The MDAR framework recommends adoption of discipline-specific guidelines, established and endorsed through community initiatives. Journals have their own policy about requiring specific guidelines and recommendations to complement MDAR.

Adherence to community standards	Information included in the manuscript?	In which section is the information available? (Reagents and Tools Table, Materials and Methods, Figures, Data Availability Section)
State if relevant guidelines or checklists (e.g., **ICMJE, MIBBI, ARRIVE, PRISMA**) have been followed or provided.	Not Applicable	
For **tumor marker prognostic studies,** we recommend that you follow the **REMARK** reporting guidelines (see link list at top right). See author guidelines, under 'Reporting Guidelines'. Please confirm you have followed these guidelines.	Not Applicable	
For **phase II and III randomized controlled trials**, please refer to the **CONSORT** flow diagram (see link list at top right) and submit the CONSORT checklist (see link list at top right) with your submission. See author guidelines, under 'Reporting Guidelines'. Please confirm you have submitted this list.	Not Applicable	

Data Availability

Data availability	Information included in the manuscript?	In which section is the information available? (Reagents and Tools Table, Materials and Methods, Figures, Data Availability Section)
Have **primary datasets** been deposited according to the journal's guidelines (see 'Data Deposition' section) and the respective accession numbers provided in the Data Availability Section?	Yes	Data Availability Section (Proteomics Data deposited to PRIDE, project ID: PXD079900). CRISPR Screen Data supplied as Dataset 1.
Were **human clinical and genomic datasets** deposited in a public access-controlled repository in accordance to ethical obligations to the patients and to the applicable consent agreement?	Not Applicable	
Are **computational models** that are central and integral to a study available without restrictions in a machine-readable form? Were the relevant accession numbers or links provided?	Not Applicable	
If publicly available data were reused, provide the respective **data citations in the reference list**.	Not Applicable	
