# Supplementary material for: Clustering of host N-glycans by the microneme MIC1/4/6 complex licenses Toxoplasma rhoptry discharge
Source: EMBO J. 2026 Sep 25;45(20):7070–102. doi: 10.1038/s44318-026-00911-z (PMC13672358; doi:10.1038/s44318-026-00911-z)
Supplement: Supplementary file 2 [file 44318_2026_911_MOESM9_ESM.pdf]

Expanded View Figures

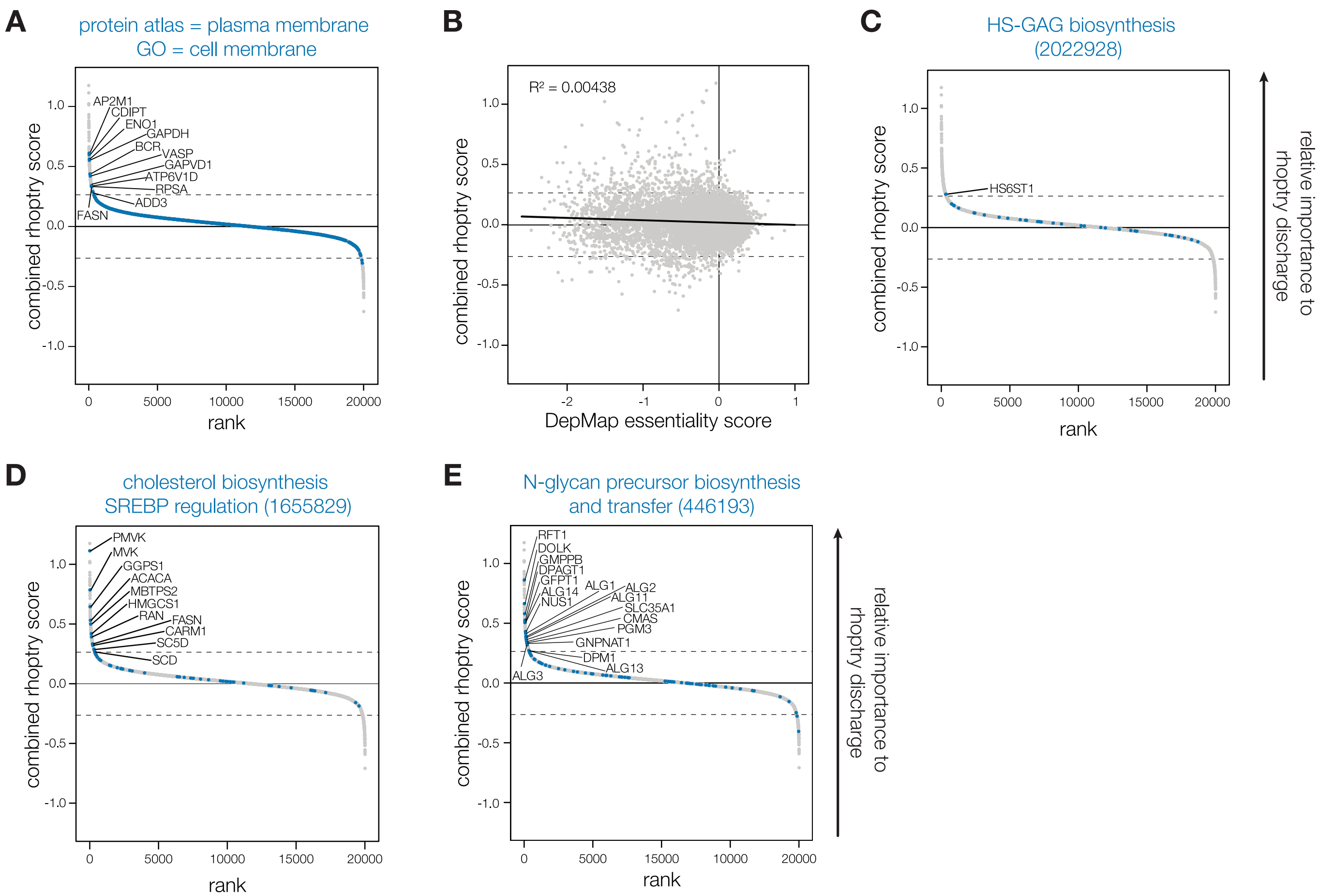

Figure EV1. Involvement of relevant gene categories in rhoptry discharge.

(**A**) Rank-ordered position of genes that are displayed in the plasma membrane or surface exposed as annotated by GO terms and the Human Protein Atlas in the rhoptry discharge screen. Dashed line indicates MLE estimation of s.d. = 3. All genes above this threshold are labeled. (**B**) Comparison of the DepMap K562 gene essentiality score to the combined rhoptry score. Regression analysis reveals nearly zero correlation between essentiality in cell culture and gene score in the rhoptry discharge screen. Dashed lines indicate MLE estimation of s.d. = 3. (**C**) Rank-ordered position of genes in the heparin sulfate GAG biosynthesis pathway in the rhoptry discharge screen. Genes displayed are from the Reactome pathway R-HSA-2022928. The dashed line indicates MLE estimation of s.d. = 3. All genes above this threshold are labeled. (**D**) Rank-ordered position of genes required for regulation of cholesterol biosynthesis in the rhoptry discharge screen. Genes displayed are from the Reactome pathway R-HSA-1655829. The dashed line indicates MLE estimation of s.d. = 3. All genes above this threshold are labeled. (**E**) Rank-ordered position of genes required for N-glycan dolichol precursor biosynthesis and transfer to a nascent protein in the rhoptry discharge screen. Genes displayed are from the Reactome pathway R-HSA-446193. The dashed line indicates MLE estimation of s.d. = 3. All genes above this threshold are labeled.

© The Author(s)

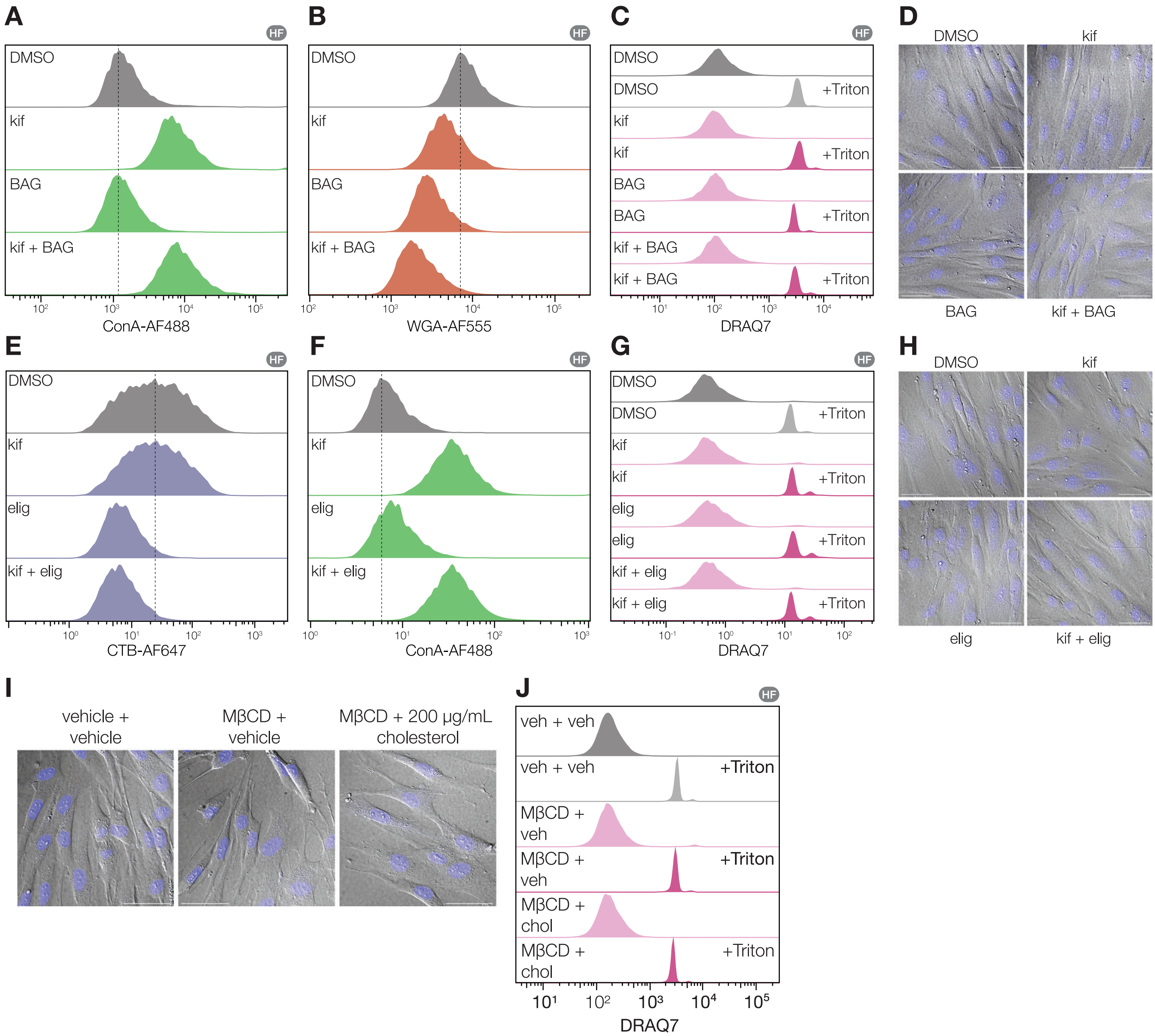

Figure EV2. Host cell drug treatments and knockouts result in expected surface glycosylation patterns.

(**A**, **B**) Flow cytometry analysis of human fibroblast surface glycosylation following a 3-day treatment with vehicle (DMSO), kif (10 µM), BAG (2 mM), or a combination of kif and BAG. Exposed mannose was stained using the lectin ConA conjugated to Alexa Fluor 488 (ConA-AF488; **A**), and exposed GlcNAc and Neu5Ac were stained using WGA conjugated to Alexa Fluor 555 (WGA-555; **B**). (**C**) Cell viability and membrane integrity of human fibroblasts following a 3-day combination of kif and BAG treatments, as evaluated by exclusion of the cell-impermeable far-red DNA dye DRAQ7. Triton X-100 (Triton; 0.02%) was added as a positive control for permeabilization. (**D**) Analysis of normal human fibroblast morphology following 3-day combinations of kif and BAG treatments, assessed by live DIC microscopy. Cell-permeable Hoechst 33342 was included to visualize nuclei (blue). A slice of a Z-stack is shown based on nuclear stain focus. White scale bars indicate 50 µm. (**E**, **F**) Flow cytometry analysis of human fibroblast surface glycosylation following a 5-day treatment with vehicle (DMSO), kif (10 µM), elig (2 µM), or a combination of kif and elig. The plasma membrane glycosphingolipid GM1 was visualized using Alexa Fluor 647-conjugated Cholera toxin B domain (CTBB-AF647; **E**), and exposed mannose was stained using the lectin ConA conjugated to Alexa Fluor 488 (ConA-AF488; **F**). (**G**) Cell viability and membrane integrity of human fibroblasts following a 5-day combination of kif and elig treatments, as evaluated by exclusion of the cell-impermeable far-red DNA dye DRAQ7. Triton X-100 (Triton; 0.02%) was added as a positive control for permeabilization. (**H**) Analysis of normal human fibroblast morphology following 5-day combinations of kif and elig treatments, assessed by live DIC microscopy. Cell-permeable Hoechst 33342 was included to visualize nuclei (blue). A slice of a Z-stack is shown based on nuclear stain focus. White scale bars indicate 50 µm. (**I**) Analysis of human fibroblast morphology following acute depletion and add-back of membrane cholesterol, assessed by live DIC microscopy. Cell-permeable Hoechst 33342 was included to visualize nuclei (blue). A slice of a Z-stack is shown based on nuclear stain focus. White scale bars indicate 50 µm. (**J**) Cell viability and membrane integrity of human fibroblasts following acute depletion and add-back of membrane cholesterol, as evaluated by exclusion of the cell-impermeable far-red DNA dye DRAQ7. Triton X-100 (Triton; 0.02%) was added as a positive control for permeabilization.

© The Author(s)

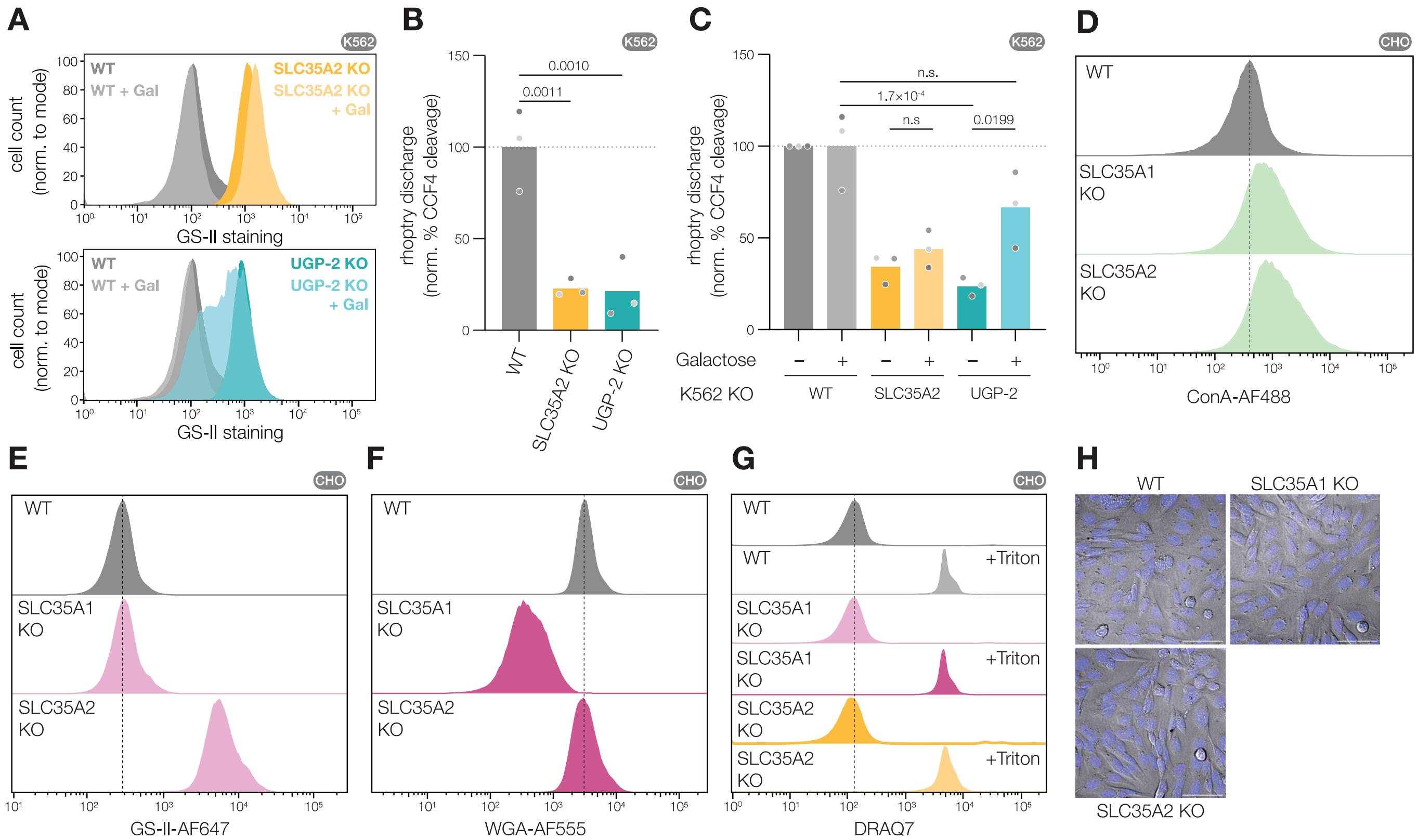

Figure EV3. Validation of host cells defective in pathways related to glycosylation.

(**A**) Flow cytometry of K562 cell lines in which UGP-2 or SLC35A2 has been knocked out (KO). Cells were labeled with GS-II, which recognizes uncapped surface GlcNAc. Galactose supplementation (25 mM, 3 days) rescues surface glycosylation patterns for UGP-2 KO but not SLC35A2 KO cells. (**B**) Rhoptry discharge was assessed in the KO cell lines as the percentage of host cells with βla-cleaved CCF4 relative to WT host cells, following challenge with rapamycin-treated DiCre/MyoH cKD/Txf-βla parasites. Mean of $n = 3$ biological replicates is plotted. *P* values derived from one-way ANOVA with Fisher's LSD post hoc test. (**C**) Rhoptry discharge into host KO cell lines grown in the presence of vehicle or galactose (25 mM, 3 days) was assessed as the percentage of host cells with βla-cleaved CCF4 relative to vehicle-treated WT K562 cells, following challenge with rapamycin-treated DiCre/MyoH cKD/Txf-βla parasites. Mean of $n = 3$ biological replicates is plotted. *P* values derived from one-way ANOVA with Tukey's post hoc test. (**D–F**) Verification of CHO cell mutants' surface glycosylation profiles by lectin-labeling and flow cytometry. CHO cells were stained with the exposed mannose-binding lectin ConA conjugated to Alexa Fluor 488 (ConA-AF488; **D**), with the terminal GlcNac-binding GS-II conjugated to Alexa Fluor 647 (GS-II-647; **E**), and with GlcNAc and Neu5Ac (sialic acid) binding WGA conjugated to Alexa Fluor 555 (WGA-AF555; **F**). (**G**) Cell viability and membrane integrity of wild type, galactosylation-deficient (SLC35A2 KO), and sialylation-deficient (SLC35A1 KO) CHO cell mutants, as evaluated by exclusion of the cell-impermeable far-red DNA dye DRAQ7. Triton X-100 (Triton; 0.02%) was added as a positive control for permeabilization. (**H**) Analysis of wild-type and mutant CHO cell morphology, assessed by live DIC microscopy. Cell-permeable Hoechst 33342 was included to visualize nuclei (blue). A slice of a Z-stack is shown based on nuclear stain focus. White scale bars indicate 50 µm.

© The Author(s)

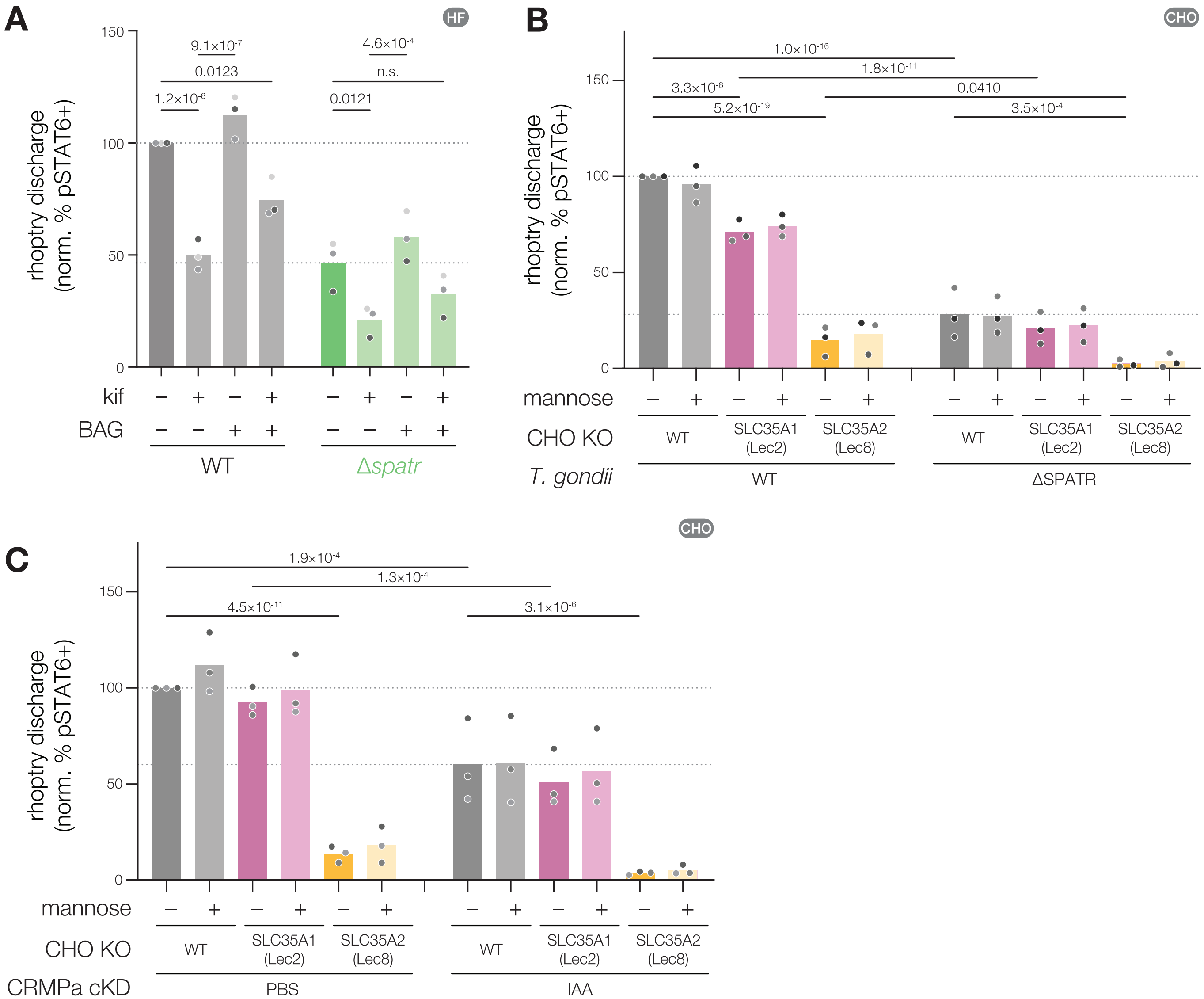

Figure EV4. Supporting data demonstrating that CLAMP and CRMP complexes additively interact with galactose and N-glycans.

(**A**) The interaction of the CLAMP complex with N-glycans and O-glycans in rhoptry discharge was assessed by treating human fibroblasts for 3 days with kif (10 µM) or BAG (2 mM) then incubating with Δ*spatr* or wild-type parental parasites. Rhoptry discharge was assessed by the relative percent of pSTAT6-positive nuclei. Mean of $n = 3$ biological replicates is plotted. *P* values were derived from two-way ANOVA with Tukey's post hoc multiple comparison test. Note that the values for the wild-type parasite condition are the same data as those in Fig. 2B. (**B**, **C**) Evaluation of the interaction of CHO galactose (SLC35A2 KO) and sialic acid (SLC35A1 KO) knockouts combined with mannose addition against CLAMP (**A**) or CRMP (**B**) complexes in rhoptry discharge. Host cells were challenged with the indicated CLAMP perturbation (wild-type or Δ*spatr* parasites; **A**) or CRMP perturbation (CRMPa cKD treated with IAA or PBS vehicle; **B**) in the presence of 10 mM of mannose. Rhoptry discharge was assessed by the relative percent of pSTAT6-positive nuclei. Mean of $n = 3$ biological replicates is plotted. *P* values are derived from two-way ANOVA testing with multiple comparison FDR correction using the Benjamini, Krieger, and Yekutieli two-stage step-up method. For both (**B**, **C**), data were analyzed as part of the same datasets as Fig. 4D and E, respectively.

 © The Author(s)

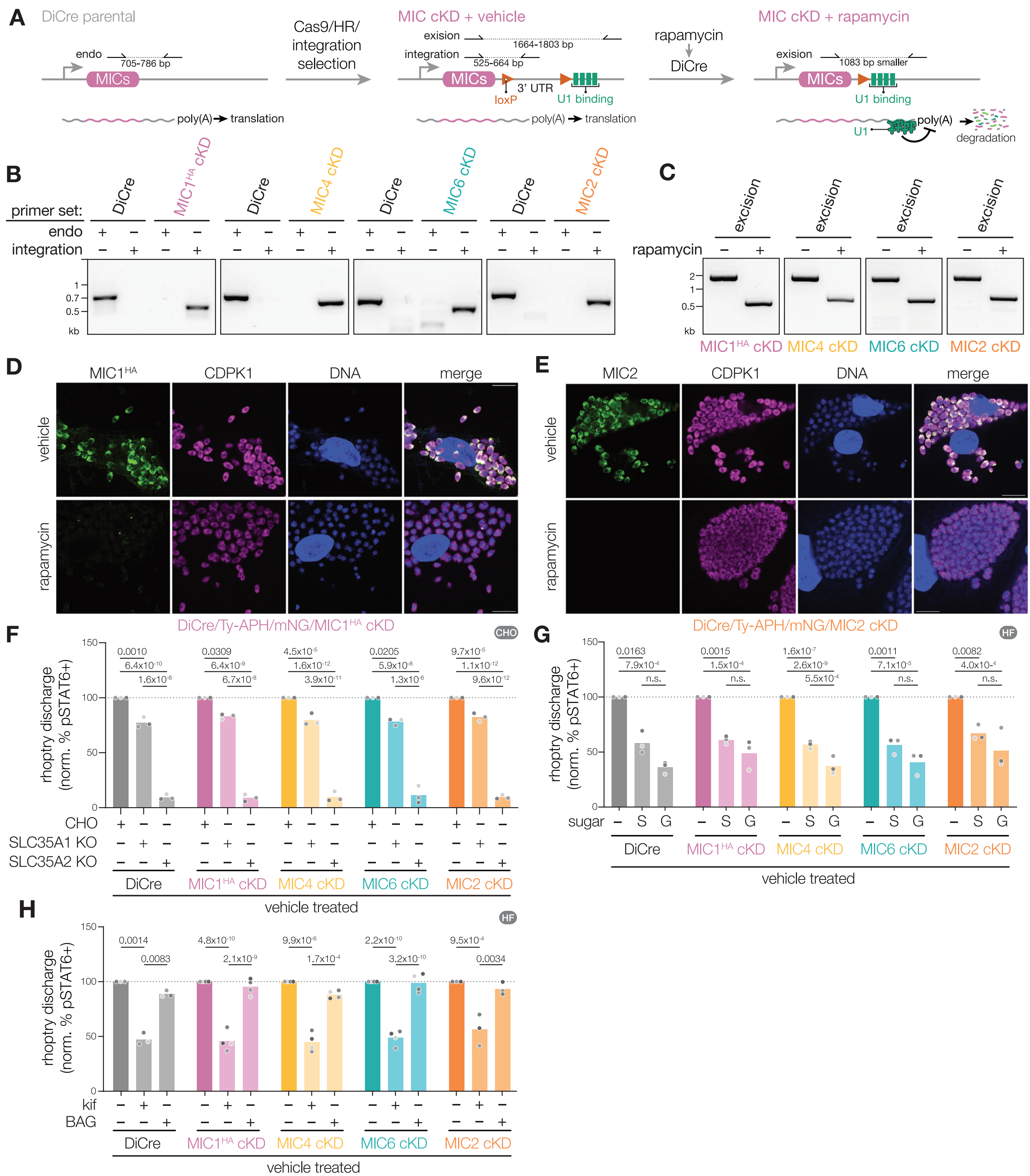
A
DiCre parental
endo
705-786 bp
MICs
poly(A) translation
Cas9/HR/
integration
selection
MIC cKD + vehicle
exision
1664-1803 bp
integration
525-664 bp
MICs
3' UTR
loxP
U1 binding
poly(A) translation
rapamycin
DiCre
MIC cKD + rapamycin
exision
1083 bp smaller
MICs
U1 binding
U1
poly(A)
degradation
B
primer set:
DiCre
MIC1HA cKD
MIC4 cKD
MIC6 cKD
MIC2 cKD
endo
integration
kb
C
excision
rapamycin
MIC1HA cKD
MIC4 cKD
MIC6 cKD
MIC2 cKD
D
MIC1HA
CDPK1
DNA
merge
vehicle
rapamycin
E
MIC2
CDPK1
DNA
merge
vehicle
rapamycin
F
DiCre/Ty-APH/mNG/MIC1HA cKD
CHO
rhoptry discharge
(norm. % pSTAT6+)
0.0010
6.4x10-10
1.6x10-8
0.0309
6.4x10-9
6.7x10-8
4.5x10-5
1.6x10-12
3.9x10-11
0.0205
5.9x10-8
1.3x10-6
9.7x10-5
1.1x10-12
9.6x10-12
CHO
SLC35A1 KO
SLC35A2 KO
DiCre
MIC1HA cKD
MIC4 cKD
MIC6 cKD
MIC2 cKD
vehicle treated
G
DiCre/Ty-APH/mNG/MIC2 cKD
HF
rhoptry discharge
(norm. % pSTAT6+)
0.0163
7.9x10-4
n.s.
0.0015
1.5x10-4
n.s.
1.6x10-7
2.6x10-9
5.5x10-4
0.0011
7.1x10-5
n.s.
0.0082
4.0x10-4
n.s.
sugar
S
G
DiCre
MIC1HA cKD
MIC4 cKD
MIC6 cKD
MIC2 cKD
vehicle treated
H
HF
rhoptry discharge
(norm. % pSTAT6+)
0.0014
0.0083
4.8x10-10
2.1x10-9
9.9x10-6
1.7x10-4
2.2x10-10
3.2x10-10
9.5x10-4
0.0034
kif
BAG
DiCre
MIC1HA cKD
MIC4 cKD
MIC6 cKD
MIC2 cKD
vehicle treated

Figure EV5. Strain validation and vehicle-treated conditions for MIC1/4/6 and MIC2 interactions with glycans in rhoptry discharge.

(**A**) C-terminal tagging and a conditional DiCre/U1 knockdown approach were used for MIC cKD strain construction ($MIC1^{HA}$ cKD, MIC4 cKD, MIC6 cKD, and MIC2 cKD). The endogenous 3′ UTR is replaced with a floxed 3′ UTR ahead of a tandem array of U1 motifs. Rapamycin treatment dimerizes DiCre, leading to 3′ UTR excision, replacement with the U1 array, and U1-mediated mRNA transcript degradation. PCRs used for endogenous loci, construct integration, and DiCre excision validations are indicated. (**B**) Agarose gels of the endogenous (endo) and construct integration PCRs demonstrating correct integration of the floxed 3′ UTRs for each cKD parasite strain. (**C**) Agarose gels demonstrating correct excision of the floxed 3′ UTR and appending of the tandem U1 array upon rapamycin treatment of each cKD parasite strain. (**D**, **E**) Evaluation of $MIC1^{HA}$ (**D**) and MIC2 (**E**) microneme localization following rapamycin treatment of cKD strains. Parasites were treated for 2 h with rapamycin, then allowed to grow intracellularly for 48 h prior to fixation and staining. anti-CDPK1 was used as a *T. gondii* cytosolic control, and Hoechst 33258 was used to stain DNA. (**F**) Challenge of vehicle-treated DiCre, $MIC1^{HA}$, MIC4, MIC6, and MIC2 cKD strains against WT, SLC35A1 KO, and SLC35A2 KO CHO cells. Control pSTAT6 assay conditions for rapamycin-induced knockdowns in Fig. 4C. Mean of $n = 3$ biological replicates is plotted. *P* values derived from analysis of each strain separately (vehicle and knockdown conditions) using two-way ANOVA with Tukey's post hoc multiple comparison test. (**G**) Challenge of vehicle-treated DiCre, $MIC1^{HA}$, MIC4, MIC6, and MIC2 cKD strains against human fibroblast in the presence of 10 mM of indicated sugar (galactose; G, sialic acid; S, or vehicle; –). Control pSTAT6 assay conditions for rapamycin-induced knockdowns in Fig. 4D. Mean of $n = 3$ biological replicates is plotted. *P* values derived from analysis of each strain separately (vehicle and knockdown conditions) using two-way ANOVA with Tukey's post hoc multiple comparison test. (**H**) Challenge of vehicle-treated treatment DiCre, $MIC1^{HA}$, MIC4, MIC6, and MIC2 cKD strains against human fibroblasts treated for 4 days with kif (10 μM) or BAG (2 mM). Control pSTAT6 assay conditions for rapamycin-induced knockdowns in Fig. 4E. Mean of $n = 3$ (DiCre and MIC2 cKD) or $n = 4$ (MIC1, MIC4, MIC6 cKDs) biological replicates is plotted. *P* values derived from analysis of each strain separately (vehicle and knockdown conditions) using two-way ANOVA with Tukey's post hoc multiple comparison test.

 © The Author(s)

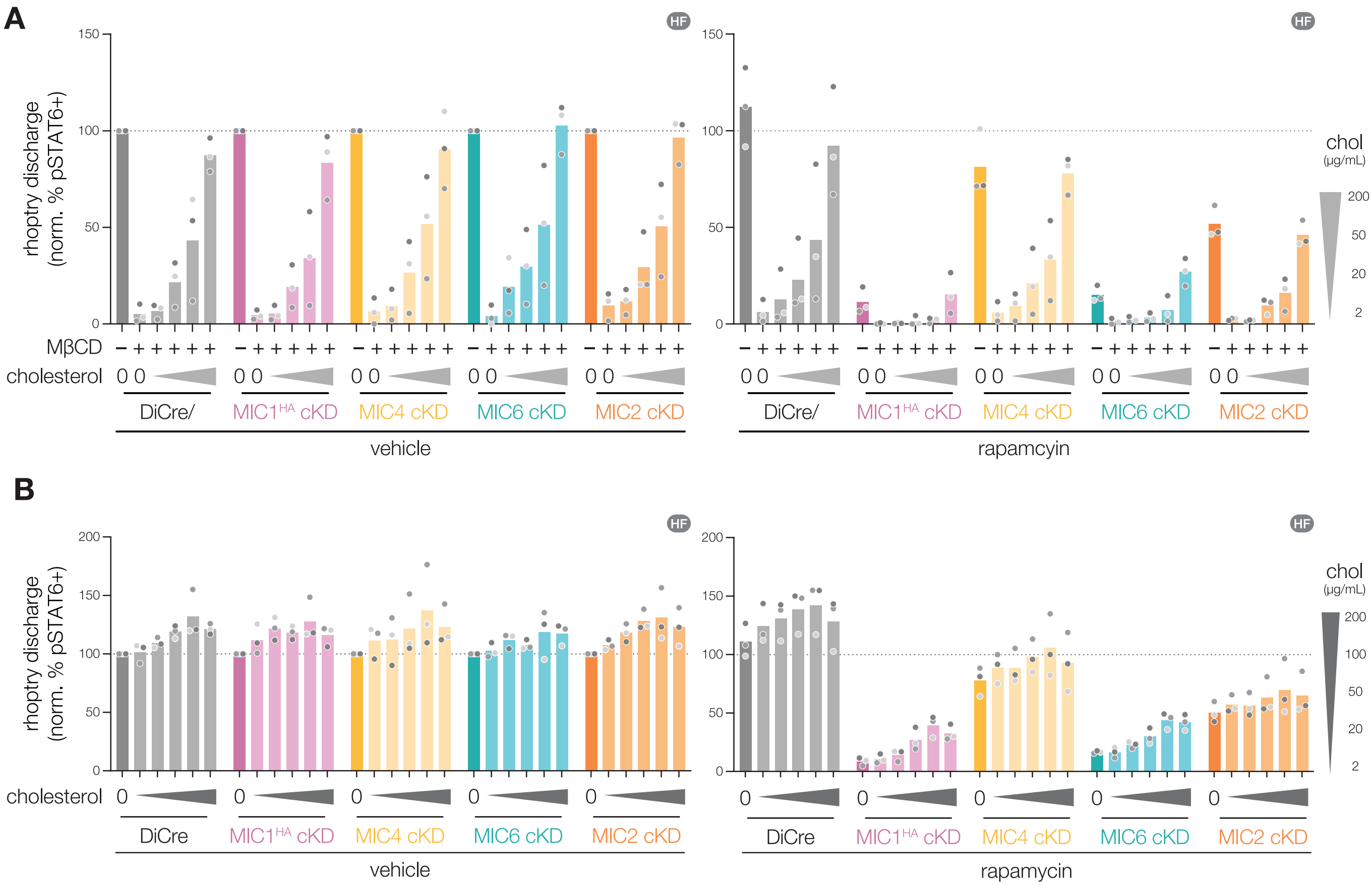

Figure EV6. Underlying data for cholesterol level modulation interactions with the MIC1/4/6 complex.

(**A**) Non-log-scaled data underlying cholesterol removal and reconstitution experiments in Fig. 6B–F. Cholesterol reconstitution levels are displayed in the legend on the right. (**B**) Non-log-scaled data underlying increased cholesterol experiments (Fig. 6B–F). Excess added cholesterol levels are displayed in the legend on the right.

© The Author(s)
